# Unprecedented Selectivity for Arsenic(III) in a Dimercaptosuccinic Acid‐Based Zr‐MOF: The Role of Dangling Ligands

**DOI:** 10.1002/anie.202516822

**Published:** 2025-09-28

**Authors:** Timo M. O. Felder, Till Schertenleib, Beatriz Mouriño, Dragos Stoian, Nazanin Taheri, Laura Piveteau, Wei Shi, Emad Oveisi, Wendy L. Queen

**Affiliations:** ^1^ Laboratory for Functional Inorganic Materials (LFIM), Institut des Sciences et Ingénierie Chimiques École Polytechnique Fédérale de Lausanne (EPFL) Rue de l'Industrie 17 Sion CH‐1950 Switzerland; ^2^ Laboratory of Molecular Simulation (LSMO), Institut des Sciences et Ingénierie Chimiques École Polytechnique Fédérale de Lausanne (EPFL) Rue de l'Industrie 17 Sion CH‐1950 Switzerland; ^3^ Swiss‐Norwegian Beamlines ESRF Grenoble BP 220 France; ^4^ Nuclear Magnetic Resonance Platform (NMRP), Institut des Sciences et Ingénierie Chimiques École Polytechnique Fédérale de Lausanne (EPFL) Lausanne 1015 Switzerland; ^5^ Research Center for Analytical Sciences Department of Chemistry College of Sciences Northeastern University Shenyang 110819 China; ^6^ Interdisciplinary Center for Electron Microscopy (CIME) École Polytechnique Fédérale de Lausanne (EPF SFL) Lausanne 1015 Switzerland

**Keywords:** Arsenic, Chelation, DMSA, Metal–organic framework, Porous adsorbent

## Abstract

While zirconium‐metal–organic frameworks (Zr‐MOFs) show promise for arsenic remediation, their practical implementation faces critical challenges: poor capture of neutral arsenite (H_3_AsO_3_ containing As(III)) and limited selectivity for arsenate (HAsO_4_^2^^−^ and H_2_AsO_4_^−^ containing As(V)) species in the presence of competing ions. Here, we present a transformative solution using dimercaptosuccinic acid (DMSA) ligands to construct two topologically distinct Zr‐MOFs. The Zr‐DMSA framework with fcu topology demonstrates exceptional capture of both arsenic species, even in the presence of phosphates, which normally compete for adsorption sites. Through comprehensive structural analysis combining PDF analysis, XAS, and solid‐state NMR, we uncover the molecular basis for this superior performance. Comparative studies with analogous thiol‐free Zr‐MOFs reveal that DMSA's thiol groups enable strong covalent sulfur‐arsenic interactions. Moreover, DFT calculations and XAS analysis illuminate an unexpected mechanism: dangling or flexible DMSA ligands enhance As(III) chelation through rotational freedom that enables effective As‐S bond formation. The remarkable selectivity for arsenate over phosphate likely stems from arsenate's reduction potential, enabling its conversion to As(III) during thiol chelation. This work not only addresses a pressing environmental challenge but also establishes how particle size effects and/or structural disorder, rather than perfect crystallinity, may dramatically enhance MOF performance for selective guest capture.

## Graphical abstract

Highly defective DMSA‐based zirconium metal–organic framework (Zr‐DMSA‐MOF) removes arsenic from water with higher selectivity over phosphate than conventional Zr‐MOFs. X‐ray absorption spectroscopy, pair distribution function analysis, and solid‐state NMR reveal that, unlike other Zr‐based MOFs where arsenic binds to the clusters, the flexible, rotatable DMSA linkers in Zr‐DMSA‐MOF enable strong, selective arsenic binding sites via chelation.

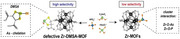

## Introduction

Water scarcity ranks among humanity's most pressing challenges, with over 2 billion people currently living in water‐stressed regions, according to the World Health Organization (WHO).^[^ ^1^ ^]^ This crisis, identified as one of the gravest global threats by the World Economic Forum (2019),^[^ ^2^ ^]^ deepens as climate change and population growth strain our already vulnerable water resources, particularly in developing nations.^[^ ^3^, ^4^ ^]^ Among the various contaminants threatening water safety, arsenic stands out as it was designated by the WHO as one of 10 chemicals of major public health concern, leading to a stringent drinking water limit of 10 ppb.^[^ ^5^ ^]^ At neutral pH, arsenic in natural waters exists primarily as arsenate (As(V))—negatively charged oxyanions HAsO_4_^2^^−^ and H_2_AsO_4_^−^ found in oxygenated surface waters—and the more toxic arsenite (As(III)), present mainly as uncharged H_3_AsO_3_ in reducing groundwater environments.^[^ ^6^, ^7^ ^]^ Though adsorption offers an energy‐efficient removal strategy, the predominance of As(III) in its uncharged molecular form (H_3_AsO_3_) severely limits the function of conventional adsorbents, which largely rely on electrostatic interactions for As(V) extraction. This creates an urgent need for innovative materials capable of capturing this elusive yet dangerous contaminant.

Metal–organic frameworks (MOFs), particularly those based on zirconium, have emerged as promising candidates for arsenate removal from water, demonstrating high adsorption capacities.^[^ ^8^, ^9^, ^10^ ^]^ The remarkable versatility of Zr‐MOFs—spanning diverse topologies and functional groups—has generated extensive research interest.^[^ ^11^, ^12^, ^13^, ^14^, ^15^, ^16^, ^17^, ^18^, ^19^ ^]^ While these materials excel at capturing arsenate, thought to occur through interactions with the oxyphilic Zr‐oxo clusters,^[^ ^20^, ^21^, ^22^ ^]^ they exhibit significantly weaker affinity for arsenite.^[^ ^23^ ^]^ Arsenate uptake is diminished in the presence of commonly found competing species like phosphate.^[^ ^20^, ^21^, ^24^ ^]^ To address this limitation, we drew inspiration from medical chelation therapy, where agents like dimercaptosuccinic acid (DMSA) effectively treat heavy metal poisoning through strong thiol–metal interactions. Among various potential chelators, DMSA stands out for its proven safety profile and ability to form complexes with arsenic that are readily expelled from the body.^[^ ^25^ ^]^ The installation of the chelators in coordination polymers is a promising approach to design selective adsorbents for water remediation. During the preparation of this work, Zhou et al. synthesized a mixed‐ligand Zr‐based MOF incorporating both DMSA and mercaptosuccinic acid (MSA), strategically designed to enhance heavy metal ion sequestration.^[^ ^26^ ^]^ Their findings demonstrate that interactions with Hg(II), Cd(II), and Pb(II) occur predominantly through the thiol (─SH) and carbonyl oxygen (COO) functionalities of the ligand. While not addressed by the authors, we believe the effective chelation of heavy metals by DMSA likely necessitates conformational flexibility afforded by freely rotatable ligands. We postulate that such nonlinking defective ligands play a pivotal role in metal chelation chemistry. Conversely, DMSA ligands that bridge between Zr sites will have less flexibility, potentially impeding their capacity for structural rearrangement during metal extraction within the more ordered MOF framework. This hypothesis finds compelling support in the work of others. For example, Peng et al. demonstrated grafting ethylenediaminetetraacetic acid (EDTA) into MOF‐808(Zr).^[^ ^27^ ^]^ Among EDTA's six potential donor atoms—comprising four oxygen atoms from carboxylate groups and two nitrogen atoms—only a single carboxylate group was observed to coordinate with the Zr cluster in MOF‐808. This anchoring configuration preserves the rotational freedom of the remaining five donor sites, rendering them accessible within the MOF's porous architecture. Such spatial liberty maintains EDTA's chelating capability, facilitating the efficient and simultaneous coordination of up to 22 distinct metal ions within this material. These observations collectively suggest that defective, conformationally flexible ligands constitute a crucial component in such systems.

DMSA can also bind As species. Although the structure of the As‐DMSA complex is not known to date, it is believed that the chelation process involves the formation of covalent bonds between the thiol groups on the DMSA molecule and the arsenic ions, resulting in a stable ring structure.^[^ ^28^, ^29^ ^]^ Leveraging this biochemical insight, we incorporated DMSA as a linker in Zr‐MOFs, synthesizing frameworks with both fcu^[^ ^30^ ^]^ and a bct topology. Zr‐DMSA‐bct is reported here for the first time. As controls, we also prepared analogous frameworks using thiol‐free succinic acid ligand (SUC) that has a similar size as that of the DMSA ligand and thus forms similar MOF structures.^[^ ^31^ ^]^ Beyond simple ligand chemistry, we anticipated that varied MOF topologies—with their distinct pore architectures—may also significantly influence arsenic capture. Surprisingly, X‐ray absorption spectroscopy (XAS), differential pair distribution function (PDF) analysis, and DFT modeling revealed an unexpected mechanism: rotationally mobile DMSA ligands enable enhanced arsenic chelation through As─S bond formation in Zr‐DMSA‐fcu relative to the other MOFs.

Using extended X‐ray absorption fine structure (EXAFS), we found that As‐DMSA chelation complexes are only formed in the material having lower crystallinity and a higher defect content, whereas the highly crystalline framework appears to be too rigid to bind As through chelation. These findings highlight the potential of disordered, low‐crystallinity MOFs constructed from nonrigid, flexible linkers. The dangling ligands improve the material's selectivity toward As(III) and As(V) over phosphate ions, a feature that has not been observed in Zr‐based MOFs until now. Local structural dynamics, rather than long‐range crystallographic order, can positively impact MOF performance in selective ion capture.

## Results and Discussion

### Characterization of Different Zr‐Based Materials

**Figure 1:**
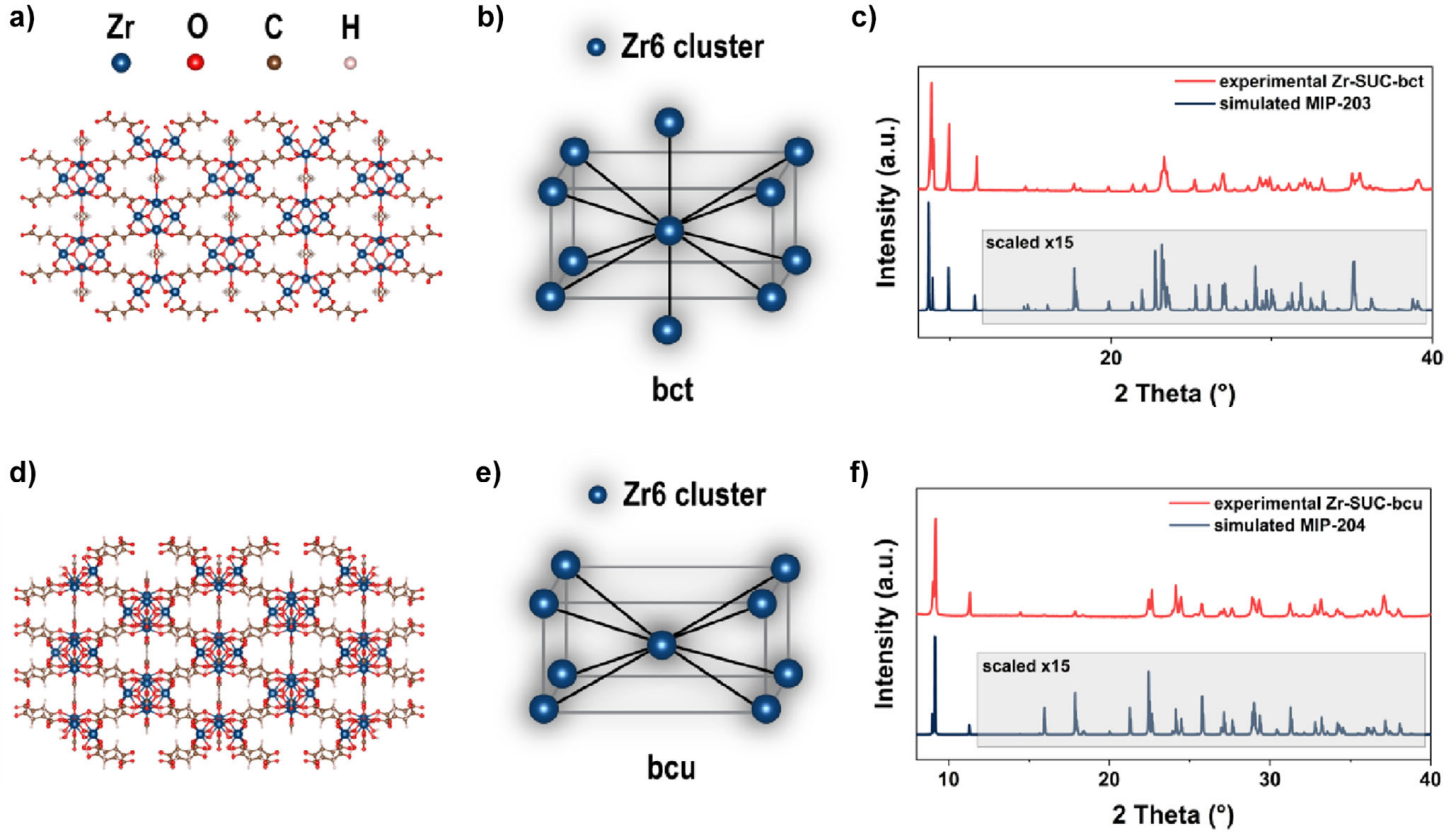
The structure model Zr‐SUC‐bct a) and b) and Zr‐SUC‐bcu d) and e), and comparison of experimentally obtained PXRD patterns with calculated pattern from CIF‐files, 15× scaled starting from 12° 2 theta c) and f).

For this study, four Zr‐based MOFs were synthesized using two different ligands—succinic acid (SUC) and meso‐2,3‐dimercaptosuccinic acid (DMSA). For succinic acid‐based MOFs, there are two reported topologies, including a body‐centered tetragonal (bct) topology, denoted Zr‐SUC‐bct (also known as MIP‐203^[^ ^31^ ^]^), and the body‐centered cubic (bcu) topology, denoted Zr‐SUC‐bcu (also known as MIP‐204^[^ ^31^ ^]^). In the present study, both topologies were obtained and found to be pure via powder X‐ray diffraction (PXRD) (Figure 1), and subsequent Le Bail fitting ensured the correct space group and unit cell parameters (see Supporting Information for details).

**Figure 2:**
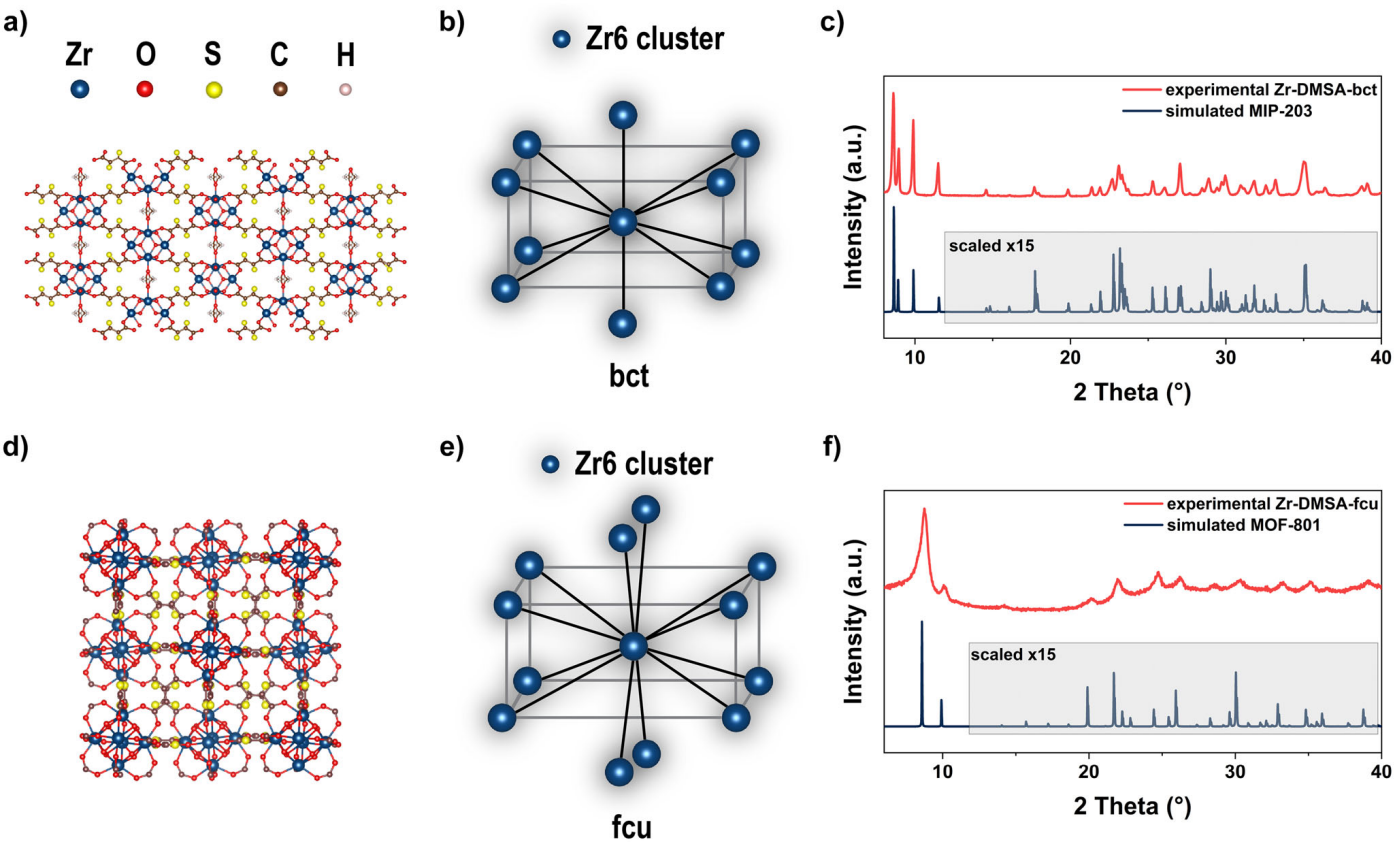
The structure model Zr‐DMSA‐bct a) and b) and Zr‐DMSA‐fcu d) and e), and comparison of experimentally obtained PXRD patterns with calculated patterns from CIF‐files from structurally related MIP‐203 and Zr‐MOF‐801, 15× scaled starting from 12° 2 theta c) and f).

**Figure 3:**
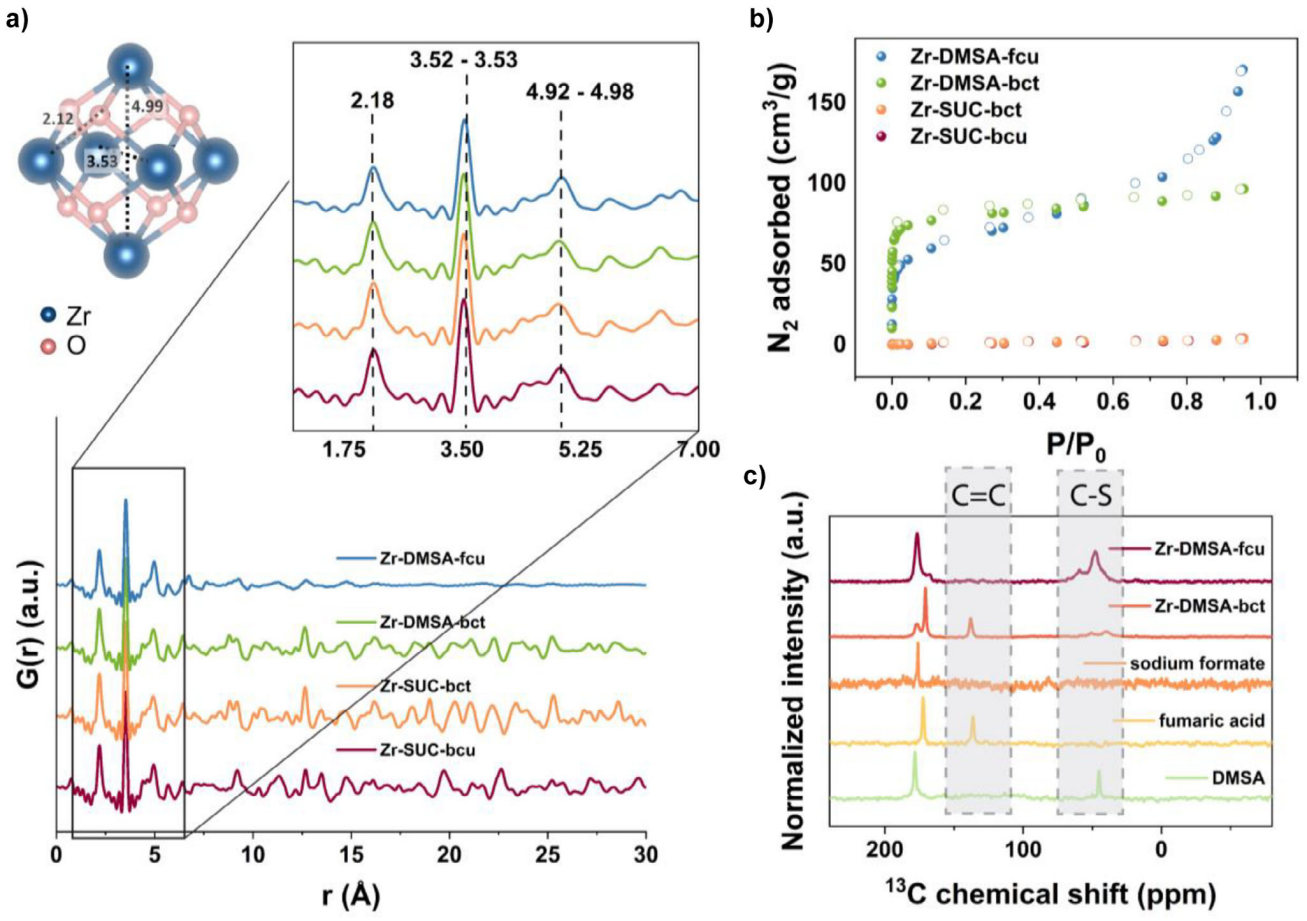
a) Structural representation of Zr_6_‐oxo cluster and pair distribution function of Zr‐SUC‐bcu (red), Zr‐SUC‐bct (orange), Zr‐DMSA‐bct (green), and Zr‐DMSA‐fcu (blue). Zoom‐in for low *r* reveals Zr–O coordination peaks at 2.18 Å and Zr–Zr coordination peaks at 3.52–3.53 and 4.92–4.98 Å, consistent with the expected Zr_6_‐oxo cluster, b) nitrogen adsorption isotherm for all MOFs at 77 K, c) ^13^C multi‐CP (multiple cross polarization) NMR spectra for Zr‐DMSA‐bct and fcu as well as fumaric acid, sodium formate, and DMSA.

For the Zr‐DMSA MOFs, only Zr‐DMSA‐fcu has been reported to date;^[^ ^30^ ^]^ although no single crystal structure of the material is available. Given this, a simulated powder pattern of MOF‐801^[^ ^32^ ^]^ was instead used to assess the purity of Zr‐DMSA‐fcu via PXRD. Importantly, MOF‐801 has the same fcu topology and is constructed by 12‐connected Zr‐oxo clusters interlinked by the fumaric acid ligand, which is comparable in length to DMSA and thus leads to similar MOF structure. Although the peak positions in the PXRD pattern of Zr‐DMSA‐fcu seem to match those of MOF‐801, the crystallinity of Zr‐DMSA‐fcu is quite poor (Figure 2f). Therefore, other characterization methods, such as PDF analysis, were also carried out. The data reveals that Zr‐DMSA‐fcu consists of the expected hexameric Zr‐oxo {Zr_6_O_4_(OH)_4_} clusters (Figure 3). Moreover, despite the absence of sharp Bragg peaks, signals at higher *r* in the PDF can be attributed to inter‐cluster Zr–Zr pairs (Figure S8), confirming that the clusters are interconnected through DMSA ligands. We surmise that the low crystallinity is mainly due to the material's small particle size (<10 nm), the linker's higher flexibility relative to fumaric acid, the thiol functionality, and/or defects that may hinder the long‐range order. Next, Zr‐DMSA‐bct was synthesized using a similar procedure as Zr‐SUC‐bct.^[^ ^31^ ^]^ The PXRD shows that Zr‐DMSA‐bct is pure and highly crystalline (Figure 2c) and Le Bail fitting ensured the space group and unit cell parameters are similar to Zr‐SUC‐bct^[^ ^31^ ^]^ (see Supporting Information for more details).

Scanning transmission electron microscopy (STEM) coupled with energy‐dispersive X‐ray spectroscopy (EDXS) was used to analyze the particle size and element distribution within the samples. We observed much bigger crystals for Zr‐DMSA‐bct (>100 nm, Figure S12) compared to Zr‐DMSA‐fcu (<10 nm, Figure S13). This agrees with the sharper Bragg peaks in the bct‐net compared to the broadened peaks of the fcu‐net (Figure 2). We also assessed the amorphous fraction in the as‐synthesized powders of the two samples; the calculations indicate that relative to the bct analogue (55.1%), the material having the fcu‐net has a much higher amorphous fraction (89.8%) (Figure S5).

Next, quantitative solid‐state nuclear magnetic resonance spectroscopy (ssNMR) data of Zr‐DMSA‐bct was collected to take a closer look at the linker chemistry in the structure. Surprisingly, the spectrum reveals that Zr‐DMSA‐bct comprises a mixture of DMSA and fumaric acid (∼60:40 ratio) linkers (Figure 3c, fitting in Figure S17). Elemental analysis of Zr‐DMSA‐bct also indicates a lower sulfur content, 5.75 ± 0.07 wt.%, than expected for the pure phase having a theoretical composition of (Zr_6_O_4_(OH)_6_(H_2_O)_2_(DMSA)_4_(HCOO)_2_).^[^ ^33^ ^]^ This implies that DMSA becomes partially desulfurized during the MOF synthesis, forming fumaric acid ligands in situ. Similar observations were previously made for MIP‐203‐M, where fumaric acid is formed in situ via dehydration reactions of malic acid.^[^ ^33^ ^]^ In contrast, the sulfur content of Zr‐DMSA‐fcu is 14.77 ± 0.10 wt.%, which is consistent with the high number of missing linkers suggested by thermogravimetric analysis (TGA) (Figure S1). This also aligns with the proposed chemical formula: Zr_6_O_4_(OH)_4_(DMSA)_2.96_(HCOO^−^)_1.09_(OH/Cl)_4.99_(H_2_O)_4.99_ (Figures S2 and S3). Please note, while the number of DMSA and formate ligands could be accurately quantified via NMR,^[^ ^34^ ^]^ a recent publication by Mathew et al. also indicates the possibility of chlorine in the structure, which led to the proposed formula.^[^ ^34^ ^]^ Further, only DMSA was found via ssNMR indicating that the ligand is stable under the conditions used to synthesize Zr‐DMSA‐fcu (Figure 3c fitting in Figure S18). STEM‐EDXS elemental maps also revealed that in both structures, sulfur is distributed homogeneously throughout the particle (Figures S20 and S21). The more crystalline bct‐net could only be obtained using higher temperatures and longer synthesis time than the fcu‐analog, which likely explains why the desulfurization only occurs in the former.

Next, we compared the porosities of the four SUC‐ and DMSA‐based MOFs. Due to the narrow pores upon solvent removal and dehydration, surface areas of the two Zr‐SUC MOFs could not be measured using N_2_ as the probe (Figure 3b).^[^ ^31^ ^]^ Therefore, CO_2_ adsorption isotherms were recorded, showing comparable results to those previously reported with calculated Langmuir surface areas of 160 m^2^ g^−1^ for Zr‐SUC‐bct and 133 m^2^ g^−1^ for bcu (Figures S9–S11 and Table 1). In contrast, the Zr‐DMSA MOFs both had N_2_ accessible pores. Zr‐DMSA‐bct had a type I adsorption isotherm and a Brunauer–Emmett–Teller (BET) surface area of 311 m^2^ g^−1^ (Figure 3b). On the other hand, Zr‐DMSA‐fcu had a mixed type I/II isotherm, likely due to the smaller particle size forming interparticle voids, and a BET area of 208 m^2^ g^−1^ (Figure 3b).

**Table 1:** Overview materials.

Material	*S*_BET_ (m^2^ g^−1^)	*S*_Langmuir_ (m^2^ g^−1^)^a)^	*K*_F_ As(V) (L g^−1^)	*K*_F_ As(III) (L g^−1^)	Qe_500_ As(III) (mg g^−1^)	Qe_500_ As(V) (mg g^−1^)
Zr‐DMSA‐fcu Zr‐DMSA‐bct Zr‐SUC‐bct Zr‐SUC‐bcu	311 208 – –	170 204 160 133	36.82 ± 2.13 6.85 ± 0.72 2.42 ± 0.21 2.38 ± 0.28	45.05 ± 1.39 6.38 ± 0.78 1.04 ± 0.17 1.06 ± 0.06	34.54 ± 6.96 4.95 ± 2.45 0.78 ± 0.69 0.76 ± 0.15	32.47 ± 6.60 5.83 ± 2.14 2.19 ± 0.58 2.58 ± 0.67

^a)^ Measured at 273 K.

Afterward, PDF analysis was used to confirm the presence of {Zr_6_} clusters in all four of the MOFs (Figure 3a), and Fourier‐transform infrared spectroscopy (FT‐IR) was used to further investigate node‐linker connectivity (Figure S6). Metal‐bound carboxylate groups appear with two distinct bands in IR, associated with symmetric and asymmetric stretching versus *v*_s_ (COO^−^) modes. Figure S6 shows that the crystalline 8‐ and 10‐connected structures (Zr‐DMSA‐bct, Zr‐SUC‐bct, and Zr‐SUC‐bcu) have multiple sharp doublets in the expected IR region, indicating several well‐defined binding modes for different ligands on the cluster surface, including DMSA, fumaric acid (for Zr‐DMSA‐bct), SUC, and capping formate ions that originate from the formic acid that is employed as a modulator during MOF synthesis.^[^ ^35^ ^]^ On the other hand, due to structural disorder, Zr‐DMSA‐fcu displays very broad peaks in the fingerprint region, likely due to the overlap of different coordination modes, e.g., *ν*_COO^−^ of the DMSA linker and the *ν*_COO^−^ of the formates. The latter are known to cap clusters that are less than 12‐connected (bct is 10‐connected while bcu is 8‐connnected)^[^ ^32^ ^]^ or compensate for missing linkers in the fcu structure.^[^ ^36^, ^37^, ^38^ ^]^

### Arsenic Adsorption

**Figure 4. a):**
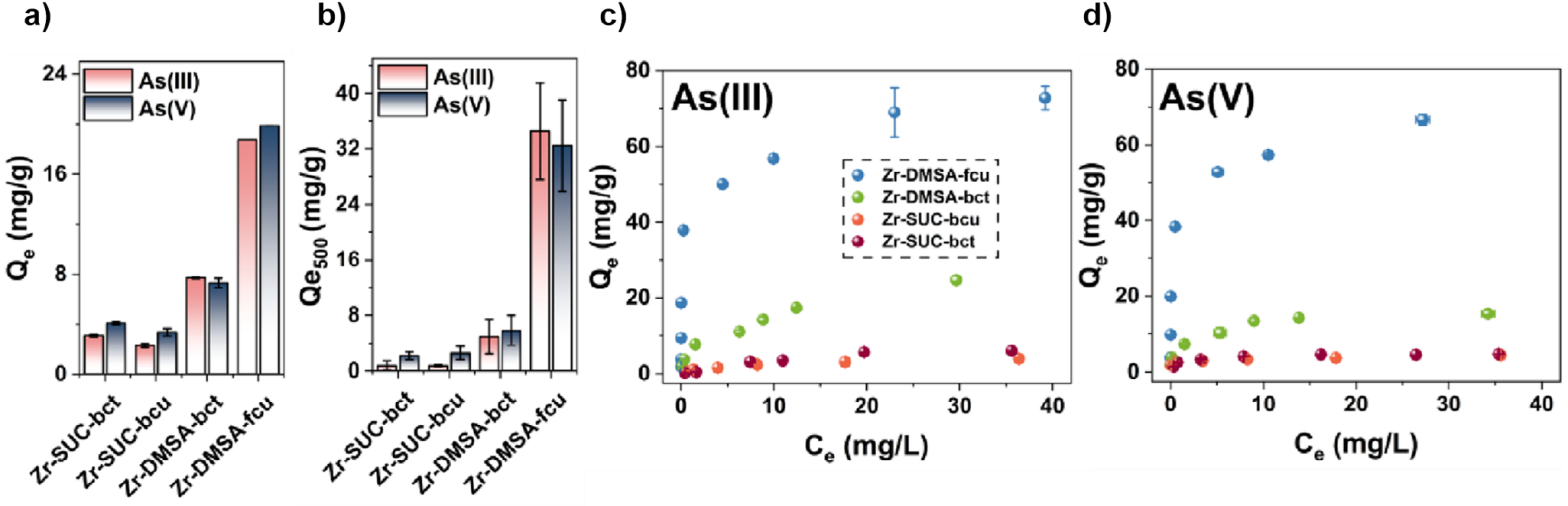
Arsenic batch adsorption experiment of different MOFs; conditions: initial concentration of As(III) (10 ppm), volume (10 mL), adsorbent dosage (0.5 g L^−1^), adsorption time (24 h), and pH (7). b) Calculated Qe from Freundlich fit of the adsorption isotherms at equilibrium concentration of 500 ppb, c) As(III), and d) As(V) adsorption isotherm for Zr‐DMSA‐fcu (blue), Zr‐DMSA‐bct (green), Zr‐SUC‐bcu (orange), and Zr‐SUC‐bct (red); conditions: initial concentration of As(III) (1–80 ppm), volume (10 mL), adsorbent dosage (0.5 g L^−1^), adsorption time (24 h), and pH (7).

Next, we investigated the role of different ligands and framework topologies on the removal efficiency of As(III) (H_3_AsO_3_) and As(V) (HAsO_4_^2^^−^/H_2_AsO_4_^−^). For this, the uptake of arsenate and arsenite was separately assessed for all four MOFs: Zr‐SUC‐bct, Zr‐SUC‐bcu, Zr‐DMSA‐bct, and Zr‐DMSA‐fcu (Figure 4). Although the thiol‐containing structures have a higher uptake for both As(III) and As(V) species, the improvement in the uptake was far more pronounced for Zr‐DMSA‐fcu compared to Zr‐DMSA‐bct. For example, starting from a 10 ppm As solution, the As(V) capacity of Zr‐DMSA‐fcu was improved by factors of 4.88, 5.9, and 2.7 when compared to Zr‐SUC‐bct, Zr‐SUC‐bcu, and Zr‐DMSA‐bct, respectively (Figure 4a). Moreover, the As(III) capacity of Zr‐DMSA‐fcu was enhanced by factors of 6.0, 8.1, and 2.4 for Zr‐SUC‐bct, Zr‐SUC‐bcu, and Zr‐DMSA‐bct, respectively. The materials’ differences are even more pronounced in the arsenic adsorption isotherm presented in Figure 4c,d. The steep slope of the adsorption isotherm for Zr‐DMSA‐fcu in the low‐concentration regime (less than 1 ppm), along with the high *K*_F_​ value obtained from the Freundlich model fit, indicates strong binding between arsenic and the framework (Figure 4c,d and Table 1). The isotherms and fitted parameters for all materials are shown in Figures S22 and S23 and Tables S3 and S4. To compare the materials performance at more relevant conditions for water purification, arsenic uptake of all materials was calculated using Freundlich fit at an equilibrium concentration of 500 ppb (Qe_500_). The Qe_500_ values presented in Figure 4b and Table 1 are given with an 80% confidence interval (more information in Supporting Information). Notably, at these equilibrium concentrations, the higher uptake of Zr‐DMSA‐fcu is even more pronounced (∼7 times higher than Zr‐DMSA‐bct).

### Mechanistic Insight

Arsenic adsorption studies reveal a distinct performance hierarchy among the four Zr‐MOFs examined: Zr‐DMSA‐fcu >> Zr‐DMSA‐bct > Zr‐SUC‐bct ≈ Zr‐SUC‐bcu in the low concentration regime—the range most pertinent to water purification applications. Understanding these performance differences required systematic examination of multiple structural and chemical parameters. For this, surface charge analysis provided initial mechanistic insight. Zeta potential measurements revealed significantly more negative surface charges for Zr‐SUC MOFs (bcu: −31.13 ± 0.61 mV, bct: −25.15 ± 1.32 mV) compared to their Zr‐DMSA counterparts (bct: −7.04 ± 0.92 mV, fcu: −4.35 ± 0.72 mV). While this electrostatic difference could partially explain the reduced adsorption of negatively charged As(V) oxyanions by Zr‐SUC frameworks, it does not tell the complete story.

Previous investigations by Audu et al. established that Zr‐oxo clusters strongly bind As(V) species but exhibit low affinity for As(III).^[^ ^23^ ^]^ Intriguingly, Zr‐SUC‐bct and Zr‐SUC‐bcu demonstrate only modest arsenic capture capacities, with As(III) uptake comparable to that of As(V). Infrared spectroscopic analysis and TGA confirm the presence of both SUC ligands and formic acid modulators coordinated to the metal clusters in these frameworks (Figures 1 and S6), while TGA indicates minimal missing‐linker defects (Figure S1). This indicates that the Zr‐oxo clusters in Zr‐SUC‐bct and Zr‐SUC‐bcu are largely saturated with carboxylate groups—a structural feature that significantly limits arsenic adsorption capabilities compared to more defective Zr‐MOF architectures reported in the literature.^[^ ^22^, ^23^ ^]^ Furthermore, considering reported pore size of these materials (∼4.5 A)^[^ ^31^ ^]^ arsenic diffusion into the pores may be somewhat limited.

We hypothesized that incorporating DMSA as a framework linker would promote As binding through thiol–arsenic interactions, providing superior performance compared to the thiol‐free Zr‐SUC frameworks. While both DMSA‐containing MOFs showed improved arsenic uptake, the adsorption isotherms for Zr‐DMSA‐bct revealed only a modest increase in As(III) (and As(V)) capture compared to Zr‐SUC frameworks (Figure 4). TGA data indicates slightly higher concentrations of missing‐linker defects in this material, which could account for this improvement (Figure S1). However, the absence of a sharp upward slope in the adsorption isotherm at low concentrations (Figure 4c,d) suggests limited arsenic affinity in Zr‐DMSA‐bct.

**Figure 5:**
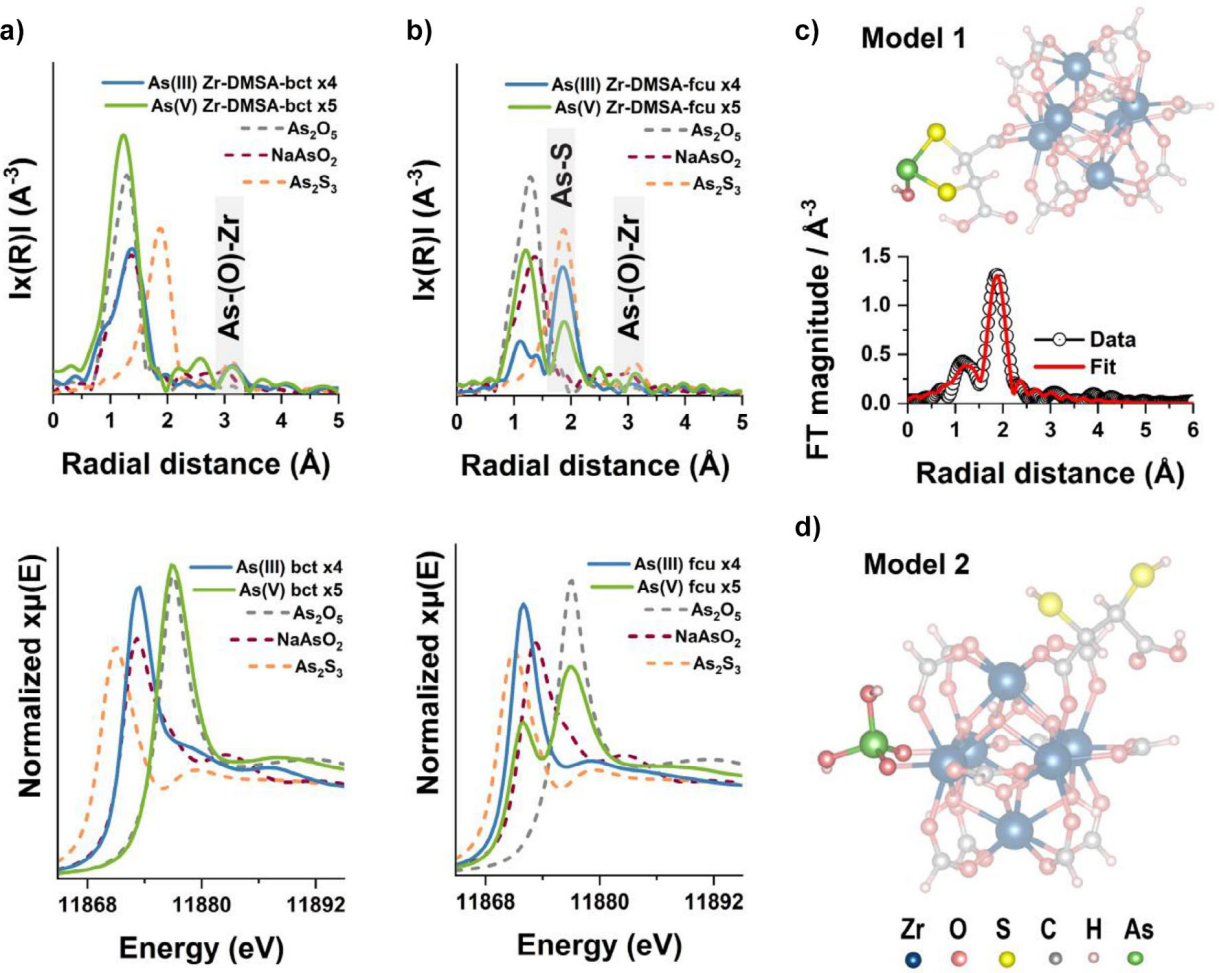
Structural investigation of arsenic adsorption via XAS for Zr‐DMSA‐bct (bct) and Zr‐DMSA‐fcu (fcu). a and b) Arsenic K‐edge EXAFS (top) and XANES (bottom) of As(V) (green) and As(III) (blue) loaded samples, including reference standards for As(V)‐oxide (grey) and As(III)‐oxide (red) and As(III)‐sulfide standards (orange). c) DFT‐optimized structural model 1 for As‐ligand interaction, including a fitting to the EXAFS data of As(III)‐loaded Zr‐DMSA‐fcu. d) DFT‐optimized structural model 2 for As‐cluster interaction.

In striking contrast, Zr‐DMSA‐fcu demonstrates dramatically higher uptake for both arsenic species compared to all other frameworks studied. While TGA data confirms substantially greater defect concentration in this material (Figure S1), which could explain its enhanced As(V) uptake, we investigated whether additional binding mechanisms might contribute to its exceptional performance. To address this question, we conducted comprehensive structural analyses using extended X‐ray absorption fine structure and X‐ray absorption near edge structure (XANES) on the two DMSA MOFs. XANES and EXAFS data were collected at the As edge for Zr‐DMSA‐bct and reference standards representing As(III)–O (NaAsO_2_), As(V)–O (As_2_O_5_), and As(III)–S (As_2_S_3_) interactions (Figure 5). Comparative analysis revealed that both arsenic species in Zr‐DMSA‐bct show adsorption edges and bond distances matching the oxide standards with no indication of As–S interactions. Moreover, EXAFS signals observed at distances greater than 3 Å (for As–O–Zr interactions) likely indicate adsorption primarily through the Zr‐oxo cluster (Figure 5a).^[^ ^20^, ^21^ ^]^ These findings confirm that DMSA does not function as an adsorption site in the bct structure, suggesting that missing‐linker defects are instead the likely culprit for its modestly improved arsenic uptake. Conversely, EXAFS data for As(III)‐loaded Zr‐DMSA‐fcu show two peaks at 1.22 and 1.87 Å (not phase corrected) corresponding to bond distances of approximately 1.78 and 2.28 Å obtained from fitting (Figure 5b and Table S22). While the former corresponds to As–O interactions (similar to As_2_O_5_ and NaAsO_2_ references), the latter matches As(III)–S interactions as evidenced by the As_2_S_3_ reference. Notably, the As–S signal appears more intense than the As–O peak, suggesting higher coordination multiplicity. Additional evidence of the unique As–S interactions in Zr‐DMSA‐fcu was obtained through differential PDF analysis of As(III)‐loaded sample, which again revealed distinct signals corresponding to As─S bond distances. In contrast, these As–S features were absent in the thiol‐free Zr‐based MOF (Figure S34).

We propose that the exclusive presence of As–S interactions in Zr‐DMSA‐fcu results from a combination of structural factors that enable conformational flexibility of DMSA ligands. In the highly crystalline Zr‐DMSA‐bct framework, DMSA ligands are rigidly constrained between Zr‐oxo clusters, positioning the thiol groups at the C2 and C3 stereocenters on opposite sides of the molecule and preventing effective arsenic chelation (Figure S31). In striking contrast, Zr‐DMSA‐fcu exhibits several features that may collectively promote ligand mobility: high structural disorder (89.81% amorphous content, Figure S5), substantial defect concentrations (Figures S1–S3), and critically, very small particle sizes (∼10 nm versus >100 nm for Zr‐DMSA‐bct, Figures S12 and S13). The small particle size is particularly significant as it creates an exceptionally high surface‐to‐bulk ratio, potentially leading to abundant surface‐terminated DMSA ligands with enhanced conformational freedom. Zhang et al. previously demonstrated that dangling ligands frequently occur as surface‐terminating groups, supporting this mechanism as a likely dominant contributor to enhanced arsenic uptake.^[^ ^39^ ^]^

To further validate this mechanism, we developed a structural model wherein only one carboxylate group of DMSA coordinates to a Zr‐oxo cluster, enabling the partially coordinated linker to rotate and chelate arsenic through both thiol groups (Figure 5c, Model 1). This DFT‐optimized model provides excellent agreement with our experimental EXAFS data (Table S22), confirming that conformationally flexible DMSA ligands facilitate the exceptional As(III) binding observed in Zr‐DMSA‐fcu. The absence of such flexibility in the larger, more crystalline Zr‐DMSA‐bct particles—likely due to their lower surface‐to‐bulk ratio and higher structural rigidity—explains their inability to achieve arsenic chelation despite containing similar DMSA functionality.

Surprisingly, XANES analysis of As(V)‐loaded Zr‐DMSA‐fcu reveals a mixture of both As(V) and As(III) species, indicating a reduction process (Figure 5b, bottom). We find that approx. 25% of the As(V) was reduced to As(III) (XANES fitting in Supporting Information, Figure S35). Correspondingly, the EXAFS data also shows a small peak at 1.87 Å matching As(III)‐S bonds (Figure 5b, top). Importantly, the As–O peak at around 1.19 Å shows a higher coordination multiplicity and we find a peak at distances above 3 Å, owed to As–O–Zr interactions. These findings suggest two distinct binding modes in As(V)‐loaded Zr‐DMSA‐fcu: As(III) interactions with the DMSA ligand (Model 1) and As(V) interactions with the Zr‐oxo cluster (Figure 5d, Model 2). DFT‐optimized models for both binding scenarios are presented in Figure 5c,d. Based on these findings, we investigated the properties and performance of Zr‐DMSA‐fcu in different competing conditions to better understand the benefits of DMSA's chelation and reductive properties.

### As Uptake in Zr‐DMSA‐fcu

**Figure 6:**
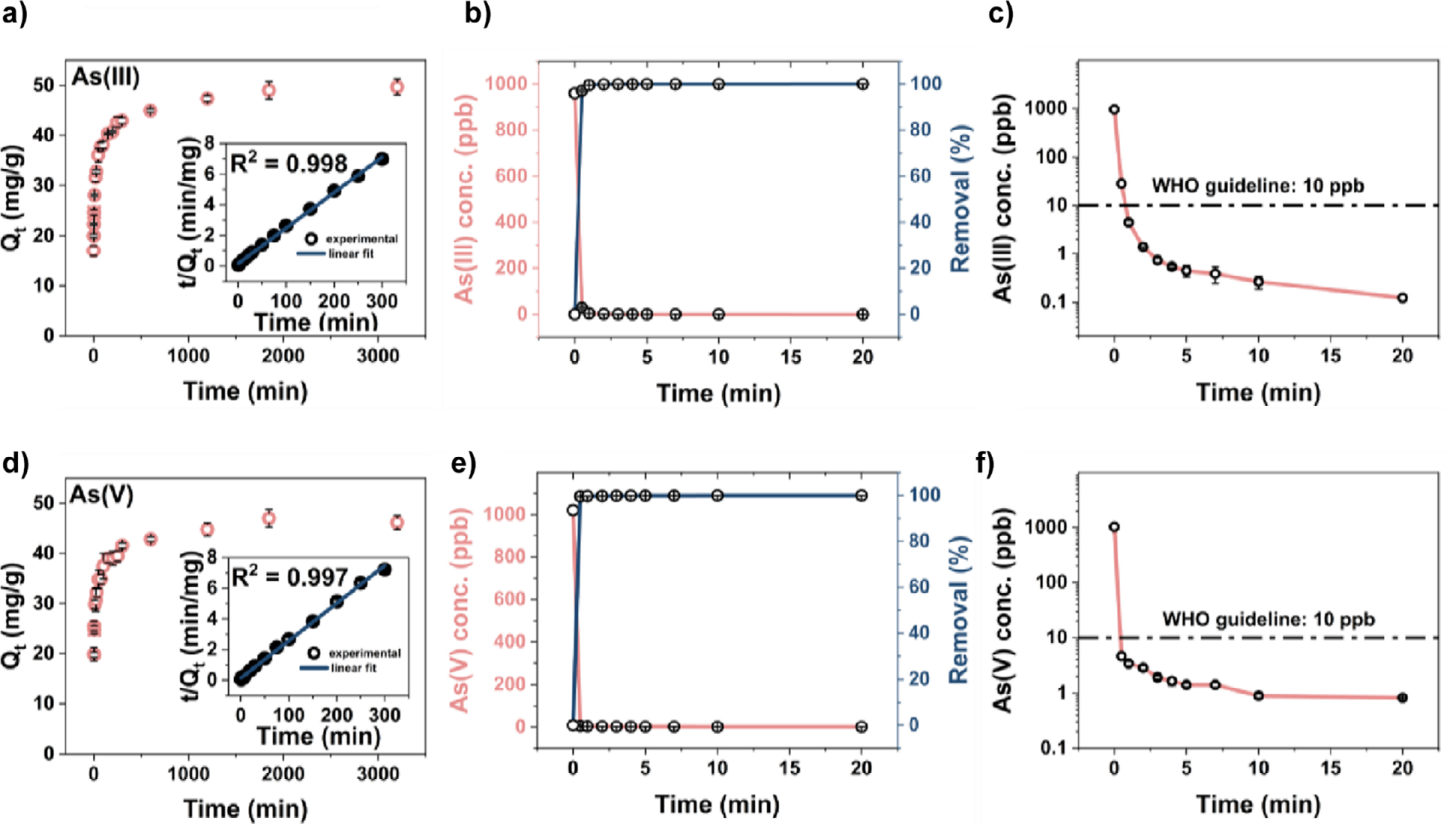
Adsorption kinetics in a 10 ppm arsenic solution with adsorbate concentration of 0.1 g L^−1^, inset shows fitted PSO model a) As(III) d) As(V). Adsorption of b and c) As(III) and e and f) As(V) from a 1 ppm solution with adsorbate concentration of 1 g L^−1^.

Insight into the speed of the As extraction was also investigated for the best‐performing material Zr‐DMSA‐fcu (Figure 6a). The experimental data was fit using the pseudo‐second‐order (PSO) model, and the resulting kinetic parameters are presented in Table S13. Figure 6a and d illustrate the fitted PSO model curves, demonstrating the agreement between the model and the experimental data. For both As species, the Zr‐DMSA‐fcu exhibited a rapid removal kinetics, characterized by removal rates of *k*_2_ = 3.0 × 10^−3^ and 3.3 × 10^−3^ g mg^−1^ min^−1^ for As(III) and As(V), respectively. In addition, an experiment conducted at a low initial concentration of 1 ppm demonstrated that the Zr‐DMSA‐fcu is capable of reducing the arsenic concentration below the World Health Organization (WHO) recommended guideline of 10 ppb in less than 30 s and 1 min for As(III) and As(V), respectively. This indicates rapid and efficient arsenic removal, highlighting its potential for water treatment applications.

**Figure 7:**
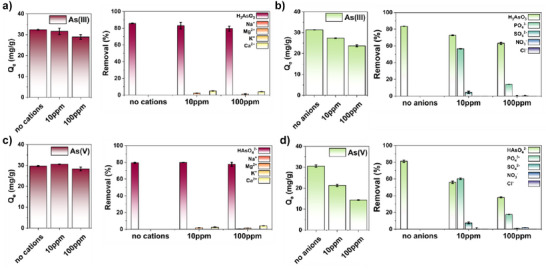
Competitive batch adsorption experiments for Zr‐DMSA‐fcu in aqueous solutions containing 1:1 (10 ppm) and 1:10 (100 ppm) arsenic:competing ion concentrations; a) As(III) and cations, b) As(III) and anions, c) As(V) and cations, and d) As(V) and anions. Conditions: initial arsenic concentration 10 ppm, pH = 7, adsorption time 24 h, adsorption dosage 0.25 g L^−1^.

Due to the similarities between anions like phosphate and arsenate, achieving selectivity for either of these oxyanions over the other poses significant challenges.^[^ ^20^, ^21^, ^24^ ^]^ Conversely, Zr‐MOFs usually demonstrate unaffected performance in the presence of other anions, namely Cl^−^, SO_4_^2−^, and NO_3_^−^, as well as various cations (e.g., Na^+^, Ca^2+^, K^+^, Mg^2+^)—ions that are commonly abundant in natural water streams.^[^ ^20^, ^21^, ^24^ ^]^ In the case of Zr‐DMSA‐fcu—adsorption experiments, carried out in solutions containing several commonly encountered cations at concentrations up to 10 times greater than arsenic, indicate that the adsorption of As(III) and As(V) is minimally influenced (Figure 7a and c). On the contrary, adsorption experiments carried out in the presence of a host of different anions (Cl^−^, NO_3_^−^, SO_4_^2−^, and HPO_4_^2−^) having concentrations up to 10 times greater than arsenic indicate a visible reduction in the adsorption of both As(III) and As(V), with As(V) experiencing a more pronounced impact. This is not surprising and stems from the co‐adsorption of HPO_4_^2−^ (Figure 7b and d). Previous studies have shown that, like arsenate,^[^ ^20^, ^21^ ^]^ phosphate binds to the Zr_6_ clusters^[^ ^40^, ^41^ ^]^ competing for adsorption sites with arsenate thereby lowering the As adsorption capacities.^[^ ^20^, ^21^, ^24^ ^]^ Interestingly, despite a 10‐fold excess of phosphates over arsenate, the arsenate remains at over 50% of its original capacity, likely indicating that there are adsorption sites present for which phosphate groups do not compete. These noncompetitive adsorption sites are likely thiol groups on DMSA ligand. Previous studies have shown that DMSA and other thiol‐containing molecules like glutathione (GSH) can reduce As(V) to As(III) and subsequently bind it.^[^ ^42^, ^43^ ^]^ As mentioned above, while As(III) mostly binds to the DMSA ligands, As(V) binds to the cluster and the DMSA ligand, where it becomes reduced and chelated. Overall, this explains the material's greater arsenite:phosphate selectivity compared to its arsenate:phosphate selectivity.

**Figure 8:**
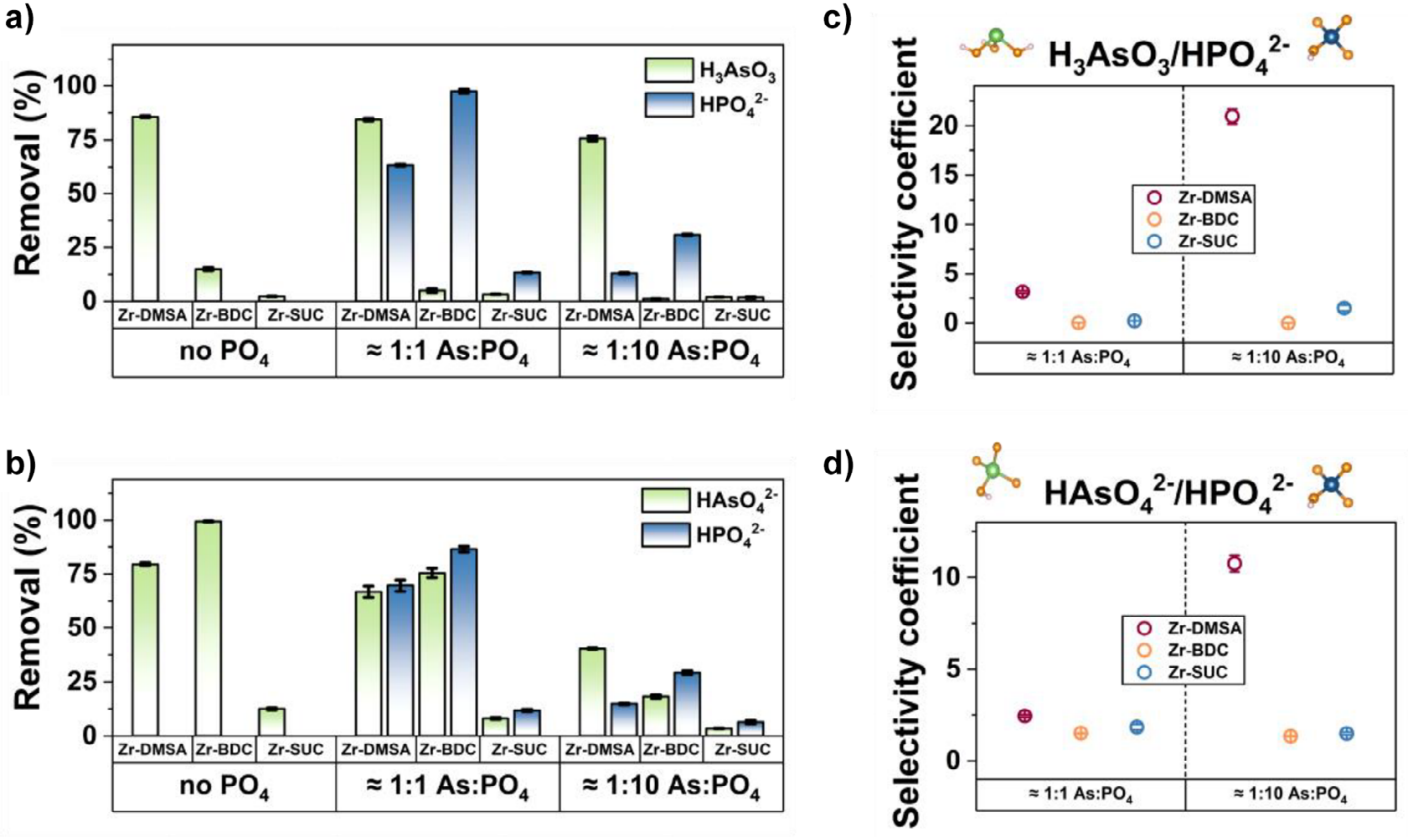
Competitive arsenic/phosphate batch adsorption experiments for Zr‐DMSA, Zr‐BDC, and Zr‐SUC‐bct in aqueous solution containing 1:1 and 1:10 arsenic:phosphate concentration; a) As(III) and b) As(V). Conditions: initial arsenic concentration 10 ppm, pH = 7, adsorption time 24 h, adsorption dosage 0.25 g L^−1^. c) calculated selectivity coefficient for presented batch adsorption experiments for As(III) and d) for As(V).

To further investigate this, binary selectivity experiments focusing on the two arsenic species (As(III) and As(V)) and phosphate were conducted. We also compared the thiol‐modified material, Zr‐DMSA‐fcu, with non‐thiol‐modified counterparts Zr‐SUC‐bct and benchmarked the results against Zr‐BDC (UiO‐66) a MOF that is well‐established for As removal in the literature (Figure 8).

Notably, the preference for thiol‐containing material Zr‐DMSA‐fcu exhibited remarkably enhanced selectivity for both As(III) and As(V) in comparison to the non‐thiol‐containing Zr‐BDC and Zr‐SUC (refer to Figure 8c,d). In particular, when subjected to a 1:10 As:HPO_4_^2−^ ratio, the adsorption efficiency of As(III) only decreased by 20% for Zr‐DMSA‐fcu, whereas Zr‐BDC experienced a substantial 90% reduction (Figure 8a). This is also reflected in the calculated selectivity coefficient (*S*_As/P_) of 20.96 ± 0.76 for Zr‐DMSA compared to 0.028 ± 0.001 for Zr‐BDC (Figure 8c and Table S16). Additionally, in the case of As(V), the Zr‐DMSA‐fcu adsorption performance exhibited a notable reduction of 51%, while Zr‐BDC was impacted to a much greater extent, with an 80% decrease (Figure 8b). By evaluating the selectivity coefficients from the 1:10 (As:HPO_4_^2−^) experiment, it becomes evident that the selectivity is substantially higher for Zr‐DMSA‐fcu (10.76 ± 0.45) compared to both Zr‐BDC (1.50 ± 0.01) and Zr‐SUC‐bct (1.48 ± 0.07) (Figure 8d and Table S17). We presume that the higher selectivity for arsenite (As(III)) can be attributed to the interaction with the introduced thiol groups in the material. Additionally, the higher selectivity of arsenate (As(V)) can be attributed to the lower redox potential of phosphate compared to arsenate, which allows for its reduction and subsequent chelation (Table S19).

Lastly, Zr‐DMSA‐fcu's performance was tested in realistic conditions with high quantities of competitive species. For this, Rhone river water was collected and subsequently spiked with either 1 ppm of As(III) or As(V). The batch adsorption measurements demonstrate that Zr‐DMSA‐fcu is able to reduce the concentration of both arsenic species well below the WHO guideline of 10 ppb in this highly complex water matrix (Figures S36 and S37).^[^ ^5^ ^]^

## Conclusion

In this work, we synthesized four different Zr‐based MOFs in an effort to systematically investigate the effects of linker chemistry and framework topology on arsenic removal: two frameworks containing succinic acid linkers (without thiols) with bcu and bct topologies, and two featuring DMSA linkers (containing thiols) with bct and fcu topologies. Arsenic uptake studies revealed that thiol‐containing frameworks consistently outperformed their thiol‐free analogs for both As(III) and As(V) removal, with Zr‐DMSA‐fcu demonstrating exceptional performance that far exceeded all other materials tested.

Comprehensive structural analysis using EXAFS and PDF techniques revealed that the superior performance of Zr‐DMSA‐fcu stems from conformationally flexible DMSA ligands that enable arsenic chelation through As─S bond formation. The small particle size (∼10 nm) and/or high degree of structural disorder in this material create abundant surface‐terminated dangling ligands with the rotational freedom necessary for effective As‐DMSA complex formation. In stark contrast, Zr‐DMSA‐bct exhibited significantly lower arsenic uptake despite containing DMSA functionality. This reduced performance was attributed to several factors: partial ligand desulfurization during synthesis (resulting in mixed DMSA/fumaric acid composition), larger particle size (>100 nm), and higher crystallinity—all contributing to a more rigid framework structure that prevents the ligand mobility required for arsenic chelation, as confirmed by XANES and EXAFS analysis.

Selectivity tests revealed that Zr‐DMSA‐fcu is highly selective for both As(V) and As(III) over numerous competing ions, with its selectivity over phosphates being particularly important. In particular, arsenate and phosphate are difficult to separate due to their similar chemical properties in water. Remarkably, XANES and EXAFS analyses demonstrated that As(V) undergoes reduction during chelation, enabling Zr‐DMSA‐fcu to selectively capture arsenate. This selectivity thus likely stems from phosphate's inability to undergo reduction, preventing its chelation by DMSA ligands—a discovery that presents a novel reductive pathway for arsenate/phosphate separation. The selectivity toward As(III) is even more pronounced since arsenite binds exclusively to DMSA, avoiding competition with phosphate for adsorption sites on the zirconium clusters. In conclusion, Zr‐DMSA‐fcu uniquely removes both arsenic species from water, even in the presence of excess phosphate. These findings contribute valuable insights toward designing advanced adsorbents that can selectively eliminate toxic species from drinking water.

## Author Contributions

T.M.O. Felder and W.L. Queen designed the work. T.M.O. Felder synthesized the materials and performed adsorption experiments and characterization. W. Shi help with adsorption experiments. T. Felder, N. Taheri, and T. Schertenleib performed total scattering experiments. Data analysis was done by T.M.O. Felder in which he was supported by T. Schertenleib, mostly in analysis of the total scattering data. NMR data was measured and analyzed by L. Piveteau. B. Mourino was leading the DFT calculations. D. Stoian analyzed the XAS data and performed EXAFS analyses. E. Oveisi performed TEM characterization. W.L. Queen coordinated and supervised the entire work. All authors discussed, read and commented on the manuscript.

## Conflict of Interests

The authors declare no conflict of interest.

## Supporting information



Supporting Information

## Data Availability

The data that support the findings of this study are openly available in Zenodo at https://doi.org/10.5281/zenodo.15489891, reference number 15489891.
