# Supplementary material for: Unprecedented Selectivity for Arsenic(III) in a Dimercaptosuccinic Acid‐Based Zr‐MOF: The Role of Dangling Ligands
Source: Angew Chem Int Ed Engl. 2025 Sep 28;64(48):e202516822. doi: 10.1002/anie.202516822 (PMC12643329; doi:10.1002/anie.202516822)
Supplement: Supplementary file 1 — Supporting Information [file ANIE-64-e202516822-s001.pdf]

Supporting Information

Unprecedented Selectivity for Arsenic(III) in a Dimercaptosuccinic Acid Based Zr-MOF: The Role of Dangling Ligands

Timo M. O. Felder[1], *Till Schertenleib*[1], *Beatriz Mouriño*[2], *Dragos Stoian*[3], *Nazanin Taheri*[1], *Laura Piveteau*[4], *Wei Shi*[5], *Emad Oveisi*[6], *Wendy L. Queen*[1]*

[1]Laboratory for Functional Inorganic Materials (LFIM), Institut des Sciences et Ingénierie Chimiques, École Polytechnique Fédérale de Lausanne (EPFL), Rue de l'Industrie 17, CH-1950 Sion, Switzerland

[2]Laboratory of Molecular Simulation (LSMO), Institut des Sciences et Ingénierie Chimiques, École Polytechnique Fédérale de Lausanne (EPFL), Rue de l'Industrie 17, CH-1950 Sion, Switzerland

[3]Swiss-Norwegian Beamlines, ESRF, BP 220, Grenoble, France

[4]Nuclear Magnetic Resonance Platform (NMRP), Institut des Sciences et Ingénierie Chimiques, École Polytechnique Fédérale de Lausanne (EPFL), 1015 Lausanne, Switzerland

[5]Research Center for Analytical Sciences, Department of Chemistry, College of Sciences, Northeastern University, Shenyang 110819, China

[6]Interdisciplinary Center for Electron Microscopy (CIME), École Polytechnique Federale de Lausanne (EPF SFL), 1015 Lausanne, Switzerland

Correspondence: wendy.queen@epfl.ch

Table of Contents

Materials and Methods 4
Chemicals 4
Synthesis of Materials 4
Characterization 5
Arsenic adsorption experiment 8
Equation arsenic adsorption experiments 9
Thermogravimetric analysis 12
TGA for Zr-SUC-bct, Zr-SUC-bcu, Zr-DMSA-bct and Zr-DMSA-fcu 12
Linker calculation for Zr-DMSA-fcu 14
Crystal Fraction Calculations 17
IR analysis 18
N_2 adsorption isotherms and BET calculation for Zr-DMSA MOFs using BETSI[1] 19
PDF analysis of Zr-DMSA-fcu 20
CO_2 adsorption isotherm of all Zr-based MOFs 21
Langmuir fits for all Zr-based MOFs at 273.15 K 23
TEM analysis of Zr-DMSA-fcu and Zr-DMSA-bct 24
Elemental Analysis 24
Quantitative ssNMR analysis 25
SsNMR of Linker 25
SsNMR of Zr-DMSA-bct and Zr-DMSA-fcu 27
STEM-EDXS analysis 30
Arsenic adsorption isotherms 32
Calculation Qe at 500ppb 35
Kinetic parameters 38
Pore size of Zr-SUC-bct/bcu 38
Zeta-potential measurement 39
Phosphate selectivity 40
Le Bail fitting for MOFs 42
Le bail fit Zr-DMSA-bct 42
Le bail fit Zr-SUC-bct 43
Le Bail fit Zr-DMSA-fcu 43
Le bail fit Zr-SUC-bcu 44
First-Principles Density Functional Theory (DFT) Calculations 45
Arsenic adsorption model 45
Arsenic chelation in rigid structure 46
Arsenic adsorption - additional considerations 47

calculated PDF and differential PDF of adsorption model 1 47
calculated PDF and differential PDF of adsorption model 2 48
Differential PDF of As(III) Zr-DMSA-fcu and UiO-66 49
X-ray absorption spectroscopy (XAS) analysis 50
XANES fitting for Zr-DMSA-fcu 50
Extended X-ray absorption fine structure (EXAFS) fitting for Zr-DMSA-fcu 52
River water experiment 53
Reproducibility of Zr-DMSA 54

Materials and Methods

Chemicals

All chemicals were used without further purification.

Synthesis of Materials

Synthesis of Zr-SUC-bct (MIP-203)

Zr-SUC-bct was synthesized following literature procedure.[1] In short, $ZrCl_4$ (233 mg, 1 mmol) and succinic acid (472 mg, 4 mmol) were added into a Teflon reactor (23 mL) followed by adding formic acid (4 mL). The resulting mixture was stirred at room temperature for 20 minutes. Then, the Teflon reactor was sealed in an autoclave and heated at 120 °C for 72 hours. After cooling down to room temperature, the crude product was centrifuged, washed with water (40 mL) and EtOH (2 x 40 mL) and dried under high vacuum overnight.

Synthesis of Zr-SUC-bcu (MIP-204)

Zr-SUC-bcu was synthesized following literature procedure.[1] In short, $ZrCl_4$ (466 mg, 2 mmol) and succinic acid (472 mg, 4 mmol) were added into a Teflon reactor (23 mL) followed by adding formic acid (4 mL). The resulting mixture was stirred at room temperature for 20 minutes. Then, the Teflon reactor was sealed in an autoclave and heated at 120 °C for 72 hours. After cooling down to room temperature, the crude product was centrifuged, washed with water (40 mL) and EtOH (3 x 40 mL) and dried under high vacuum overnight.

Synthesis of Zr-DMSA-bct

Zr-DMSA-bct, first time reported here, was synthesized inspired by MIP-203 synthesis procedure[1]; In short, $ZrCl_4$ (233 mg, 1 mmol) and meso-2,3-dimercaptosuccinic acid (729 mg, 4 mmol) were added into a Teflon reactor (23 mL) followed by adding formic acid (8 mL). The resulting mixture was stirred at room temperature for 20 minutes. Then, the Teflon reactor was sealed in an autoclave and heated at 120 °C for 72 hours. After cooling down to room temperature, the autoclave was decanted and the crude product was centrifuged, washed with

extensively with EtOH (6 x 40 mL), and dried under high vacuum overnight. Note, that during the reaction sulfuric vapors may be produced. Only open Teflon reactor in ventilated fume hood.

Synthesis of Zr-DMSA-fcu

Zr-DMSA-fcu was synthesized adapting literature procedure.[2] $ZrCl_4$ (1165 mg, 5.0 mmol), meso-2,3-dimercaptosuccinic acid (910 mg, 5 mmol), and 340 μL of FA were dissolved in 10 mL of water. After ultrasonic treatment for 1 min, the mixture was kept in an oven at 95 °C for 12 h. The solid was then washed with H_2O (2 x 40 mL) and ethanol (6 x 40 mL). Finally, the sample was freeze dried.

Synthesis of Zr-BDC (UiO-66)

Zr-BDC was synthesized adapting literature procedure. In a 1L cap-screw vessel, $ZrCl_4$ (2400 mg, 10.3 mmol) was dissolved in DMF (180 mL) followed by addition of 18 mL of HCl (37%). Separately, terephthalic acid (2209 mg, 13.3 mmol) was dissolved in DMF (360 mL), sonicated and added to the mixture. The mixture was additionally sonicated for 15 minutes followed by heating at 80°C for 24 hours. After cooling down to r.t., the crude product was placed in 50 mL centrifugation vessels, washed with DMF (3 × 40 mL) and solvent exchanged with EtOH (3 × 40 mL). Finally, the sample was freeze dried under high vacuum overnight.

Characterization

Powder X-ray diffraction (PXRD)

PXRD was collected on either lab-based Bruker D8 Discover instrument or with synchrotron X-ray source. Lab based PXRD was measured on a Bruker D8 Discover system with Cu Kα source (1.54056 Å) at 40 kV and 40 mA. The primary optics slit and secondary optics slit were set to 12 mm and 9 mm respectively with a NiO filter. The scanning range was set from 2-80°. Synchrotron based PXRD was measured at wavelength of 0.244860 Å with PILATUS3 X CdTe 2M detector. Simulated powder patterns and structures were generated using vesta software.[3] Le bail fits were done using Topas software. CIF file used for MIP-203[4], MOF801: COD:

4121459[5], MIP-204 the simulated CIF file was obtained from the authors of *Computational Structure Determination of Novel Metal-Organic Frameworks*[4].

Nitrogen adsorption measurements

Nitrogen adsorption measurements were done on a Belsorp Max-II instrument at 77K. The samples were activated at 125°C prior to the measurement. The specific surface area was calculated by using BETSI software.[6]

Fourier-transform infrared spectroscopy (FTIR)

FTIR spectra were collected using a PerkinElmer Frontier MIR/FIR spectrometer. Samples were pressed on a diamond window and spectra were recorded between 4000 and 400 cm^{-1} at a resolution of 2 cm^{-1}.

Thermogravimetric analysis (TGA)

TGA curve was obtained using a TA Q-Series TGA Q500. The balance flow rate was at 15 mL/min with nitrogen. The thermal stability profile was measured in 40 mL/min air flow and a ramp of 5°C per minutes up to 800°C.

MAS ssNNR

^{13}C solid-state NMR spectra were recorded on a 11.7 T (500 MHz) Bruker spectrometer equipped with an AvIII HD console and a 3.2 mm triple-channel HXY CPMAS probe. Samples were packed into 3.2 mm zirconia rotors under ambient conditions and spun at 20 kHz spinning speed using nitrogen gas. Multiple cross polarization (multiCP) experiments[7] consisted of 8 CP blocks, with each 1 ms contact time and a linearly varying amplitude from 90-100%. The depolarization period lasted 2.6 s and recycle delays were set to 1.3 x T_1 of protons. Up to 2048 transients were cumulated for each spectrum. ^{13}C chemical shifts were referenced relative to tetramethylsilane using adamantane as secondary reference at 1.82 ppm. The spectra were fitted using the dmfit software.[8]

XAS ex-situ measurement

Samples for ex-situ measurements were prepared exposing 15 mg of Zr-DMSA-bct and Zr-DMSA-fcu to a 40mL of 100ppm arsenic solution (both As(III) or As(V)). The mixture was kept shaking at 200 rpm for 24 hours, the powder was collected by centrifugation and vacuum

dried. Next, the powders were loaded into 1 mm glass capillaries. As edge of the samples were measured in transmission and fluorescence mode.

Pair distribution function (PDF) measurement

For Pair distribution function analysis, total scattering measurements were conducted at the Swiss-Norwegean Beam Line (SNBL)BM31 at the European Synchrotron Radiation Facility (ESRF) in Grenoble, France. Samples were packed into glass capillaries with inner diameter of 1 mm and wall thickness of 0.01mm. Next, sample were mounted on a capillary spinner. A PILATUS3 X CdTe 2M detector was placed at an angle of 15° 0.1888m downstream of the sample to maximize the accessible Q-range. For each sample, 40 images with an acquisition time of 30 s were collected. 2D diffraction images were averaged and integrated using PyFAI.[9] An empty glass capillary was measured and subtracted from the 1D diffractograms prior to data reduction. Data reduction was done using PDFgetX3.[10] The Fourier transform from F(Q) to G(r) was done using Qmax = 25 ˚A, Qmin = 0.1 ˚A, and rpoly = 1.4, step size 0.01.

Density functional theory (DFT) calculation

We performed Density Functional Theory calculations with the Gaussian16 software, to obtain energy-minimized structure models and compute binding energies. Calculations were performed using the CAM-B3LYP functional, with the D3 version of Grimme's dispersion correction with Becke-Johnson damping. The basis set def2-SVP was used for carbon, hydrogen and oxygen, while the basis set def2-TZVP and the associated Stuttgart/Dresden effective core potential replacing 28 core electrons were used for zirconium and arsenic.[11,12] The geometry of each cluster was optimized with an RMS force cutoff of $1*10^{-5}$, with a pruned grid of 175,974 for first-row atoms and 250,974 for atoms in the second and later rows.

Electron Microscopy

Scanning transmission electron microscopy (STEM) data were acquired under high-angle annular dark-field (HAADF) conditions using a probe-corrected Thermo Fisher Scientific Spectra 200 S/TEM operated at 200 kV with a beam current of 250 pA. The microscope is equipped with an ultra-high brightness cold field emission gun (X-CFEG), a Super-X EDS system comprising four silicon drift detectors, and Velox acquisition software. Energy-dispersive X-ray spectroscopy (EDXS) data were collected as spectrum images, in which a focused electron probe was raster-scanned across a region of interest in STEM mode. Selected area electron diffraction patterns were acquired using the same instrument.

Arsenic adsorption experiment

In general, for arsenic adsorption experiments a stock-solution was prepared by dissolving appropriate arsenic salt (As_2O_5 for As(V) and $NaAsO_2$ for As(III)) in Milli Q water. After adjusting the pH to 7 using HCl and NaOH, the solution was diluted to appropriate concentrations for arsenic adsorption experiment.

Batch adsorption experiment

All batch adsorption experiments were measured in triplicates. For this, 5mg of dry MOF powder was weighted out in a 40 mL glass vial, followed by adding 20 mL of arsenic solution. The vials were closed and kept shaking (200 rpm) at room temperature for 24 hours. The samples were then filtered through 0.22 um pore PTFE syringe filters into a 15 mL centrifuge tube. From this solution, 4 mL were transferred to another 15 mL centrifuge tube and 0.085 ml nitric acid (69 %) were added. The arsenic concentration was then quantified using ICP-OES. Adsorption capacities and arsenic removal were calculated based on the different arsenic concentration before and after adding the MOF powder to the solution. (see equation arsenic adsorption experiment).

Arsenic adsorption isotherm

For isotherm measurements, 5 mg of dry MOF powder was weighted out in a 20 mL glass vial, followed by adding 10 mL arsenic solution with different concentration (1-100ppm). Arsenic isotherm data was fitted using Langmuir and Freundlich equation (see equation arsenic adsorption experiment).

Selectivity experiment

For selectivity experiment, 10ppm arsenic solution containing either no other ions, 10 ppm or 100 ppm of anion/cation were prepared by dissolving appropriate metal salts in arsenic solution. For cations, chloride salts of K^+, Ca^{2+}, Mg^{2+} and Na^+ were used. For anions, sodium salts for Cl^-, SO_4^{2-} and NO_3^-, and potassium salt for PO_4^{3-} were used. Finally, anion concentration in solution before and after solution was measured using Ion chromatography whereas the cation and arsenic concentration was measured using ICP-OES.

Kinetic arsenic experiments

For kinetic experiment two different experiments at high (10 ppm) and low (1ppm) arsenic concentration were conducted. For high concentration 10 mg of dry MOF powder was weighted out in a 250 mL glass jar, followed by adding 100 mL arsenic solution (10 ppm). Aliquots (2 mL) were taken at different time points, followed by analysis via ICP-OES. For low concentration, 30 mg of MOF powder was weighted out in a 40 mL glass vials, followed by adding 30 mL arsenic solution (1 ppm). Aliquots (1 mL) were taken at different time points, followed by analysis via ICP-MS.

Equation arsenic adsorption experiments

Adsorption capacities and arsenic removal

Adsorption capacities and arsenic removal were calculated based on the different arsenic concentration before and after adding the MOF powder to the solution (equation 1 and 2).

Q_e was calculated with the equation given bellow;

$$Qe = \frac{(C0 - Ce) * V}{m} \ (eq\ 1)$$

Q_e = the amount of arsenic adsorbed at equilibrium (mg g^{-1})

C_0 = initial arsenic concentration (mg L^{-1})

C_e = the concentration of arsenic at equilibrium (mg L^{-1})

V = Volume (L)

m = mass of adsorbent (g)

Removal (%) was calculated using the given equation bellow;

$$Removal\ (\%) = \frac{C0 - Ce}{C0}\ x\ 100\ (eq\ 2)$$

Q_e = the amount of arsenic adsorbed at equilibrium (mg g^{-1})

C_0 = initial arsenic concentration (mg L^{-1})

C_e = the concentration of arsenic at equilibrium (mg L^{-1})

Adsorption isotherm fitting

The arsenic isotherm was fitted using two different equations. The Langmuir and the Freundlich equation are given bellow; equation 3 and 4, respectively

$$Qe = \frac{q_m b C_e}{1 + (bC_e)} \ (eq\ 3)$$

Q_e = the amount of arsenic adsorbed at equilibrium (mg g^{-1})

C_e = the concentration of arsenic at equilibrium (mg L^{-1})

q_m = the maximum adsorption capacity (mg g^{-1})

b = Langmuir constant (L mg^{-1})

$$Q_e = K_F C_e^{1/n} \ (eq\ 4)$$

Q_e = the amount of arsenic adsorbed at equilibrium (mg g^{-1})

C_e = the concentration of arsenic at equilibrium (mg L^{-1})

K_F = the affinity constant for adsorption (L g^{-1})

n = the index of heterogeneity

Adsorption kinetic fitting

For kinetic adsorption experiments, the experimental data fitted using a pseudo-second-order kinetic model (PSO). The PSO assumes that the rate-limiting step in the adsorption process involves the chemisorption of the metal ions onto the adsorbent surface. The PSO equation is given in equation 5;

$$\frac{t}{q_t} = \frac{1}{k^2 {Q_e}^2} + \frac{t}{Q_e} \ (eq\ 5)$$

Q_e = the the amount of arsenic adsorbed at equilibrium (mg g^{-1})

k = rate constant for PSO model (g (mg min)$^{-1}$)

t = time (min)

Calculation of distribution coefficient (K_d) and selectivity coefficient (S)

Distribution coefficient (Kd) were calculated using the equation 6 bellow;

$$K_d = \frac{Q_e}{C_e} \ (eq\ 6)$$

Q_e = the amount of arsenic adsorbed at equilibrium (mg g^{-1})

C_e = the concentration of arsenic at equilibrium (mg L^{-1})

Selectivity for arsenic and phosphorous was calculated using equation 7 bellow;

$$S_{As/P} = \frac{K_{d\ (As)}}{K_{d\ (P)}} \text{ and } S_{P/As} = \frac{K_{d\ (P)}}{K_{d\ (As)}} \ (eq\ 7)$$

K_d = distribution coefficient (mL g^{-1})

Thermogravimetric analysis

TGA for Zr-SUC-bct, Zr-SUC-bcu, Zr-DMSA-bct and Zr-DMSA-fcu

Figure S1 shows the TGA data measured under an air atmosphere of the as-synthesized Zr-based materials. For all materials (**Figure S1a-d**), there are two main steps observed. The first step between 25-200 °C can mainly be attributed to the loss of solvent molecules, adsorbed water and the removal/degradation of formic acid. The weight loss between 200-800 °C is due to the dehydration of the Zr-oxo cluster and the decomposition of the organic linkers. The remaining material at 800°C is low-crystalline ZrO_2. To qualitatively assess the presence of linker defects, all TGA data sets were rescaled so that the residue weights (ZrO_2) align at 100 wt.%. Samples with a lower wt.% of organics will have a lower wt. % at T=200 °C. Next, theoretical weight losses of defective-free structures were calculated for each MOF (displayed in red). For charge balance, we consider two cases for the calculation; I) all linker-free coordination sites are capped by H_2O and ^-OH and II) coordination sites are charge balanced by $HCOO^-$, resulting in theoretical formulas of $Zr_6O_4(OH)_4(H_2O)_4(OH)_4(linker)_4$ and $Zr_6O_4(OH)_4(H_2O)_4(HCOO)_4(linker)_4$ for 8 connected bcu structures, $Zr_6O_4(OH)_4(OH)_2(H_2O)_2(linker)_4(HCOO)_2$ and $Zr_6O_4(OH)_4(H_2O)_2(HCOO)_2(linker)_4(HCOO)_2$ for 10 connected bct structures and $Zr_6O_4(OH)_4(linker)_6$ for fcu structure. Next, we assume complete decomposition of organic linkers at 800°C to H_2O and CO_2 forming ZrO_2. Therefore, 1 mole of MOF turns into 6 moles of ZrO_2. The theoretical weight loss can then be calculated using equation 8 and 9, respectively;

$$MW_{MOF} = \frac{W_{200C}}{W_{800C}} * 6M\ ZrO_2 \quad (eq\ 8)$$

When we set W_{800C} to 100 % and rearrange to W_{200C} we get;

$$W_{200C} = \frac{MW_{MOF}}{6M\ ZrO_2} * 100 \quad (eq\ 9)$$

As an example, the theoretical weight percent range at 200 °C for Zr-SUC-bcu can be calculated using its MW of 1283.81 mol/g ($Zr_6O_4(OH)_4(H_2O)_4(OH)_4(linker)_4$) and 1395.91 mol/g ($Zr_6O_4(OH)_4(H_2O)_4(HCOO)_4(linker)_4$). The MW divided by 739.3 mol/g (6 x (MW ZrO_2)) followed by multiplication by 100 gives 173.65 % and 188.81 %. All other theoretical wt. % were then calculated using the same procedure. According to **Figure S1 a** and **c**, we can see that both Zr-SUC-bcu and Zr-SUC-bct MOF show experimental weight losses very close to the

theoretical weight loss of a defective free molecular formula, indicating neglectable linker defects. For the Zr-DMSA-bct, the theoretical weight calculation was modified based on the fact that part of the DMSA was converted to fumaric acid making it a mixed linker system. SsNMR showed a ratio of DMSA/fumarate of approx: 60:40 % (**Figure S15, Table S2**), therefore the theoretical formula was assumed to be $Zr_6O_4(OH)_6(H_2O)_2(DMSA)_{2.4}(fumarate)_{1.6}(HCOO)_2$. Comparing the experimental weight of Zr-DMSA-bct to the theoretical weight, we note that the weight is slightly lower than theoretically expected which means less organics which in turn indicates the presence of missing linkers. This is also in agreement with the low sulfur content found in Elemental analysis (**Table S1**). Finally, we compare the experimental data for Zr-DMSA-fcu and note that we find a much lower weight as the calculated theoretical weight percent, indicating a significant amount of linker defects.

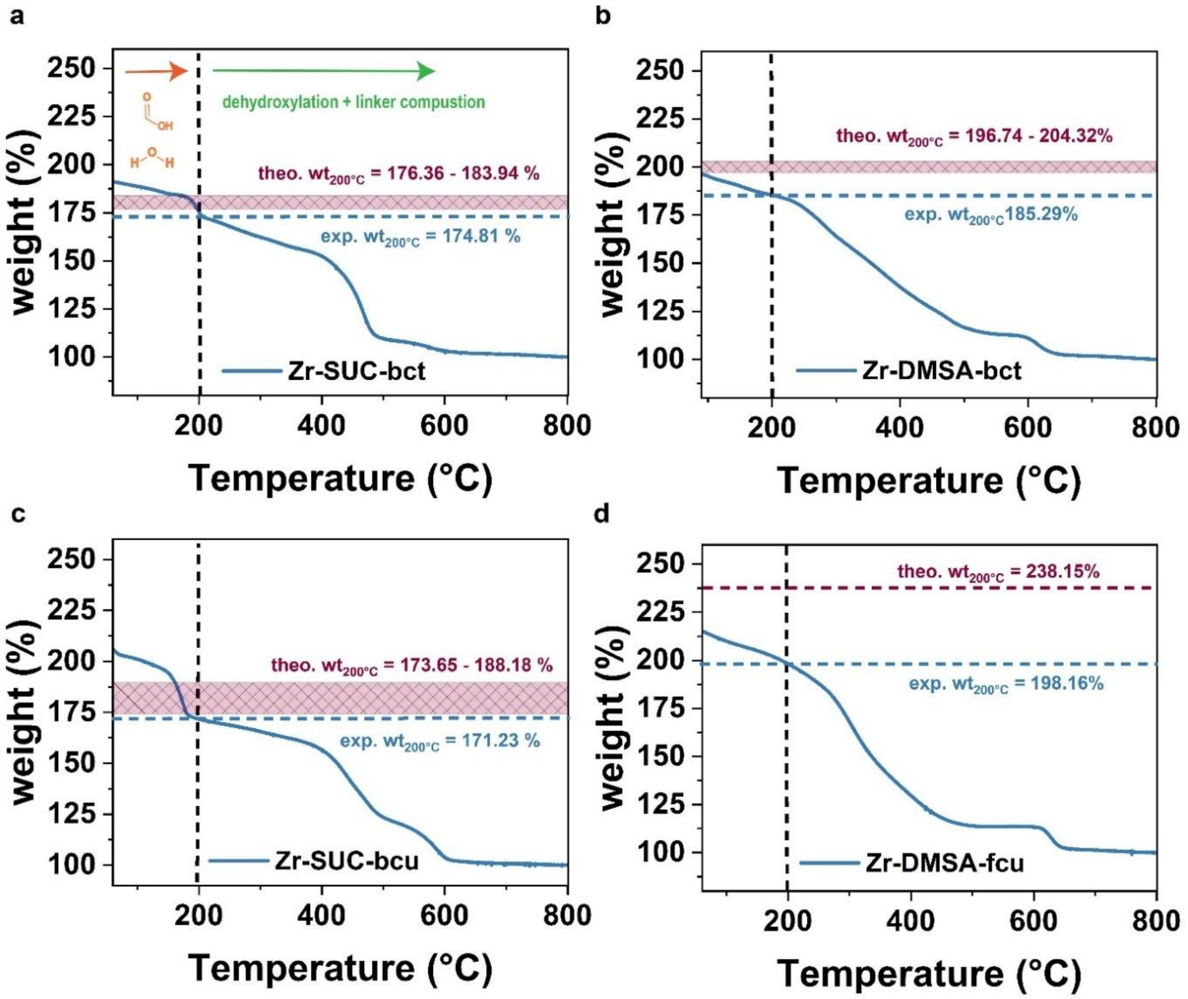

Figure S1 Thermogravimetric analysis profile of a) Zr-SUC-bct, b) Zr-DMSA-bct, Zr-SUC-bcu and d) Zr-DMSA-fcu. Theoretical weight for defective free MOFs at 200°C is calculated for each structure (red) and compared to experimental weight (blue).

Linker calculation for Zr-DMSA-fcu

For Zr-DMSA, the amount of DMSA linker in the structure was determined using quantitative NMR adapted from recently published quantitative protocol.[13] For this we assume that missing linkers are charge compensated by capping formates ($HCOO^-$), hydroxides (^-OH) or chlorides (^-Cl), leading to a formula:

$Zr_6O_4(OH)_4(DMSA)_{6-x}$(charge compensating species)$_{2x}$

Step 1: digestion of Zr-DMSA-fcu

In the first step, a precise amount of Zr-DMSA-fcu was weighed and digested in 1 mL of 1 M K_2CO_3 solution in D_2O to obtain a MOF concentration of 1.53 mmol/L. The amount of Zr-DMSA-fcu used was calculated based on the room temperature molecular weight determined by TGA (**Figure S2a**), which was found to be 1627.56 g/mol. Accordingly, 2.49 mg of Zr-DMSA-fcu was weighed and dissolved in 1 mL of the carbonate solution. Under basic conditions, DMSA oxidizes to fumaric acid. To ensure complete oxidation, the samples were sonicated at 60 °C for 3 days. Every 8 hours, the solutions were saturated with oxygen by bubbling pure O_2 (10 mL/min for 3 minutes) through the sample. After oxidation for 3 days, there is no signal corresponding to DMSA (3-4 ppm) in the ^1H NMR (**Figure S3**), therefore the concentration of fumarate (FUM) in solution corresponds to the original DMSA content.

Step 2: Quantitative NMR of FUM and $HCOO^-$

To determine the concentrations of fumarate (FUM) and formate ($HCOO^-$) in solution, disodium terephthalate (BDC) was used as an internal standard. A volume of 490 µL of the MOF digestion solution was mixed with 10 µL of a 0.1 M solution of the sodium salt of BDC, resulting in final concentrations of 1.5 mM for Zr-DMSA-fcu and 2 mM for BDC. This solution was analyzed using a Bruker Avance III HD 400 NMR spectrometer at 198 K. To ensure a quantitative NMR measurement, the delay time (D1) for a 90° pulse must be at least five times longer than the longitudinal relaxation time (T_1) of the slowest-relaxing species. Therefore, T_1 values for FUM, $HCOO^-$, and BDC were determined via inversion recovery experiments (180° pulse followed by a 90° pulse and a variable delay; see **Figure S2b**), using each analyte dissolved in 1 M carbonate solution. T_1 values obtained using the TopSpin relaxation module were: 19 s for FUM, 23.3 s for $HCOO^-$, and 3.54 s for BDC. Consequently, D1 was set to 120 s to ensure accurate quantification.

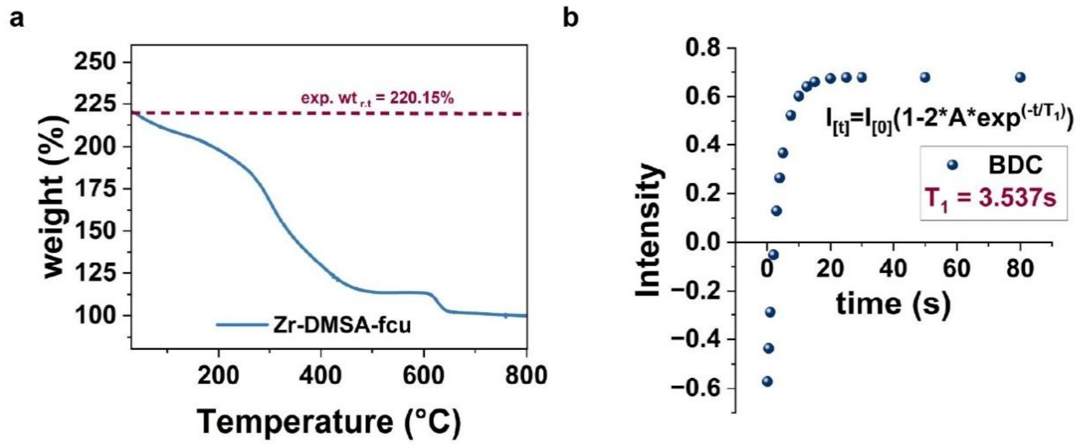

Figure S2 a) TGA curve of Zr-DMSA highlighting the wt.% of Zr-DMSA-fcu at r.t., b) inversion recovery ^1H NMR experiment to determine T_1 for Na_2BDC.

Step 3: Determination of concentration of FUM and $HCOO^-$

The measured quantitative NMR for a solution containing 1.5mM Zr-DMSA-fcu (based on molecular weight at room temperature) and 2 mM BDC is presented in **Figure S3**.

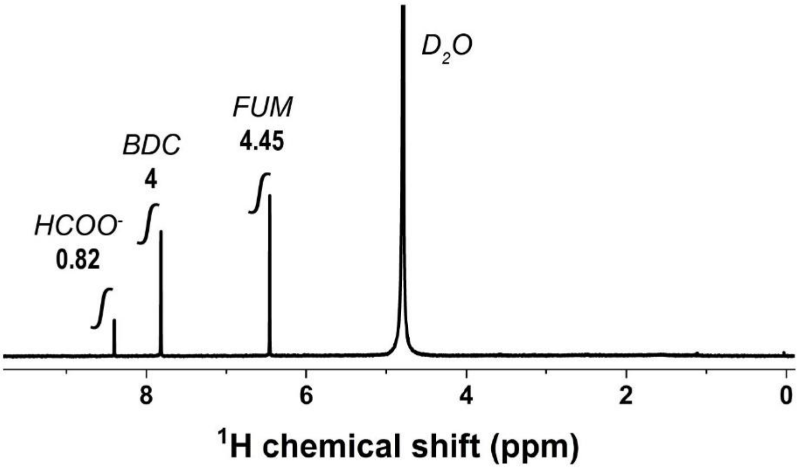

Figure S3 ^{1}H NMR spectrum in D_2O of 1.5 mM Zr-DMSA digested using 1M K_2CO_3, with 2 mM Na_2BDC as internal standard.

Since our internal standard, BDC (2 mM), exhibits a single peak corresponding to four equivalent protons, its integral is set to 4. This allows the relative concentrations of the other analytes to be determined by comparing integrals. Using Equation 10, the concentrations of FUM and $HCOO^-$ in solution can be calculated:

$$[\boldsymbol{analyte}] = \frac{\frac{\boldsymbol{I_{analyte}}}{\boldsymbol{n_{analyte}}}}{\frac{\boldsymbol{I_{BDC}}}{\mathbf{4}}} \times [\boldsymbol{BDC}] \ \ (eq\ 10)$$

where $I_{analyte}$ is the integral of the analyte signal (FUM or $HCOO^-$), and $n_{analyte}$ is the number of protons contributing to that signal.

Using this equation 10, we calculated the concentrations of the analytes in solution. Given that the MOF concentration is 1.5 mM, we can determine the molar ratios of DMSA (x) and $HCOO^-$ (y) per formula unit of the MOF:

$$x = \frac{[FUM]}{[Zr - DMSA - fcu]} = \frac{4.45}{1.5} = 2.96\ (eq\ 11)$$

$$y = \frac{[HCOO^-]}{[Zr - DMSA - fcu]} = \frac{1.64}{1.5} = 1.09 \; (eq\ 12)$$

This yields the following empirical formula for the MOF:

$Zr_6O_4(OH)_4(DMSA)_{2.96}(HCOO)_{1.09}$(charge compensating species)$_{2x.}$

Finally, we must ensure charge balance for the six Zr^{4+} cations in our MOF formula, which includes negatively charged hydroxide or chloride ligands. Based on STEM-EDXS analysis of Zr-DMSA-fcu (**Figure S21**), we observe the presence of chloride ions (Cl^-), indicating that some of the charge compensation is achieved via Cl^- in addition to OH^-. We therefore include this mixed contribution as a combined term in the general formula:

$Zr_6O_4(OH)_4(DMSA)_{2.96}(HCOO)_{1.09}(OH/Cl)_z(H_2O)_{z.}$

To determine the value of z, we apply charge balance as follows:

$n_{Zr} \times Q_{Zr} + n_O \times Q_O + n_{OH} \times Q_{OH} + n_{DMSA} \times Q_{DMSA} + n_{HCOO} \times Q_{HCOO} + z_{OH/Cl} \times Q_{OH/Cl} = 0$

where n is the number of atoms, and Q is the corresponding charge;

$6 \times 4 + (4 \times -2) + (4 \times -1) + (2.96 \times -2) + (1.09 \times -1) + (z \times -1) = 0$

$24 - 8 - 4 - 5.92 - 1.09 – (z \times -1) = 0$

$z \times (– 1) = 4.99$

Thus, the final empirical formula becomes:

$Zr_6O_4(OH)_4(DMSA)_{2.96}(HCOO^-)_{1.09}(OH/Cl)_{4.99}(H_2O)_{4.99.}$

Crystal Fraction Calculations

We determined the crystalline fractions of our samples from synchrotron powder X-ray diffraction (SPXRD) data using a background subtraction procedure. The crystalline fraction is calculated according to equation 13:

$$F_{cryst.}(\%) = \frac{I_m - I_{poly}}{I_m - I_{bkg}} \times 100\ (eq\ 13)$$

where I_m is the measured signal of the sample, I_{poly} is the fitted Chebyshev polynomial, and I_{bkg} is the fitted background signal from a Si standard measurement (**Figure S4**). For a fully amorphous sample, the fitted polynomial would coincide with the measured data, yielding $I_{poly} = I_m$. The plotted fitted signals for Zr-DMSA-fcu and Zr-DMSA-bct are shown in **Figure S5**.

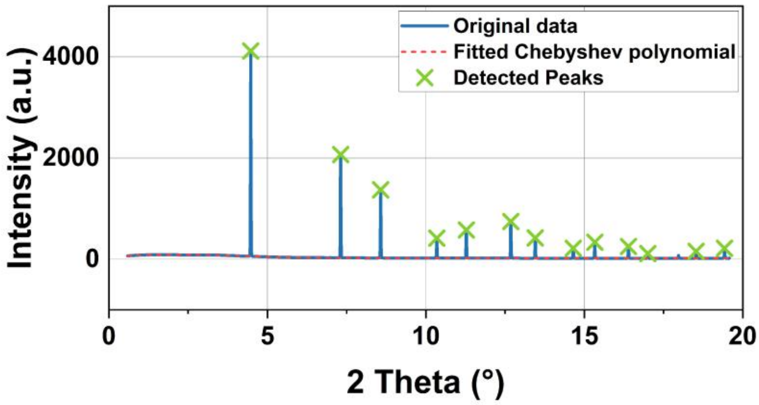

Figure S4 Determination of background intensity from a Si standard measurement using a Chebyshev polynomial fit.

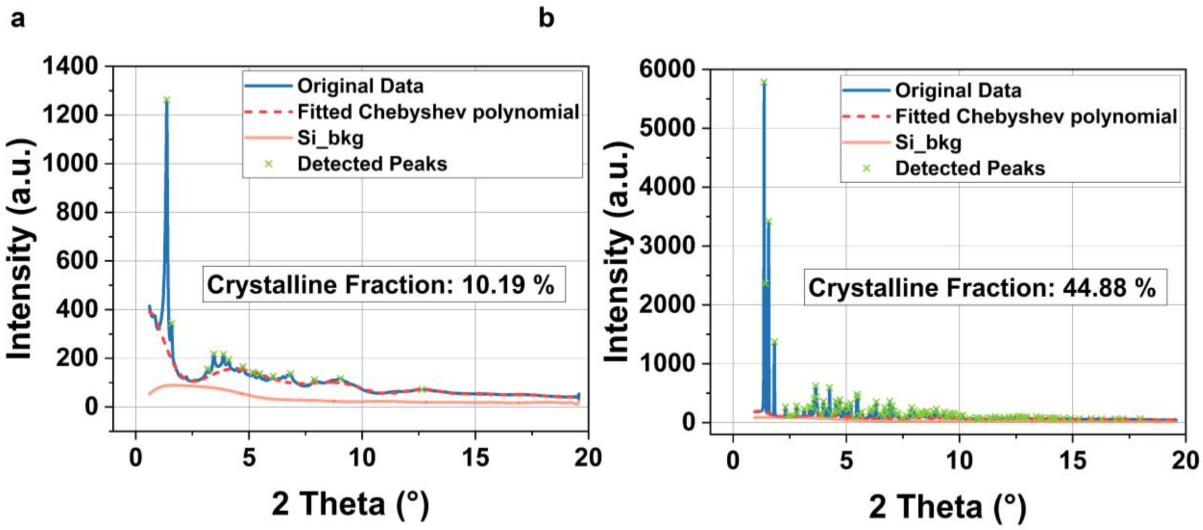

Figure S5 Calculation of crystalline fraction: Determination of the crystalline and amorphous fraction in a) Zr-DMSA-fcu and b) Zr-DMSA-bct

IR analysis

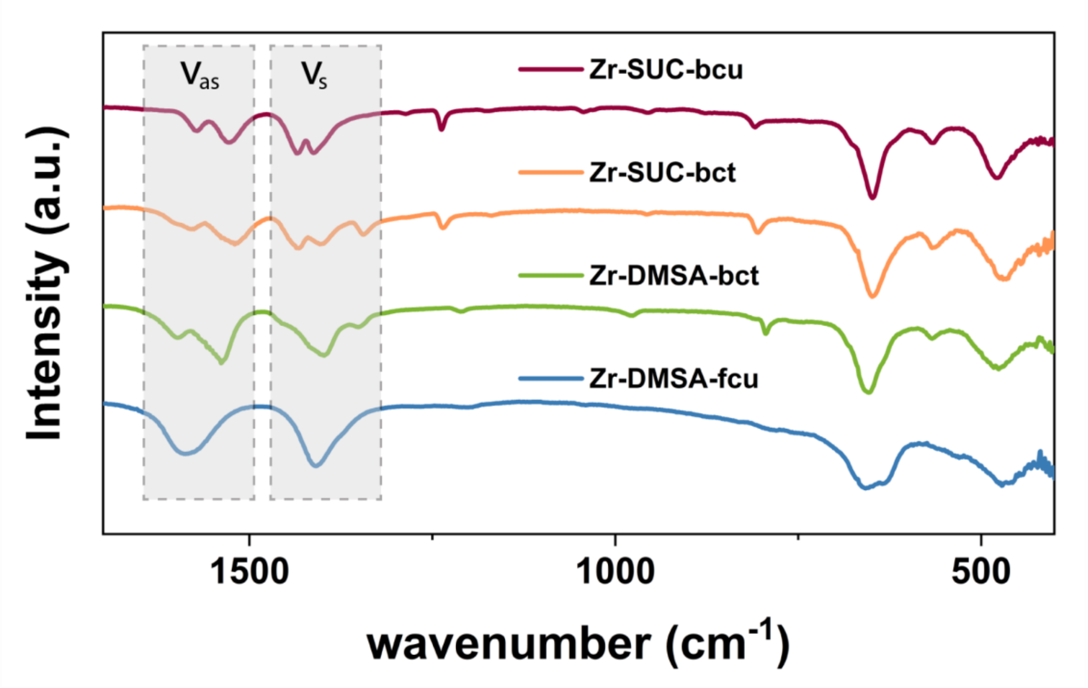

Figure S6 IR of Zr-SUC-bcu (red), Zr-SUC-bct (orange), Zr-DMSA-bct (green) and Zr-DMSA-fcu (blue). Highlighted grey area shows asymmetric and symmetric $v(COO^{-})$ of carboxylate ligands coordinated to the cluster.

N_2 adsorption isotherms and BET calculation for Zr-DMSA MOFs using BETSI[1]

Brunner-Emmett-Teller (BET) was calculated using BETSI software.[6] For this, the BET area criteria were selected as follows: Minimum number of points in the linear region: 7, Rouquerol criterion 1: Monotonic, Rouquerol criterion 2: Positive C: Rouquerol criterion 3: Pressure in linear range, Rouquerol criterion 4: Error in %: 10, Rouquerol criterion 5: End at the knee.

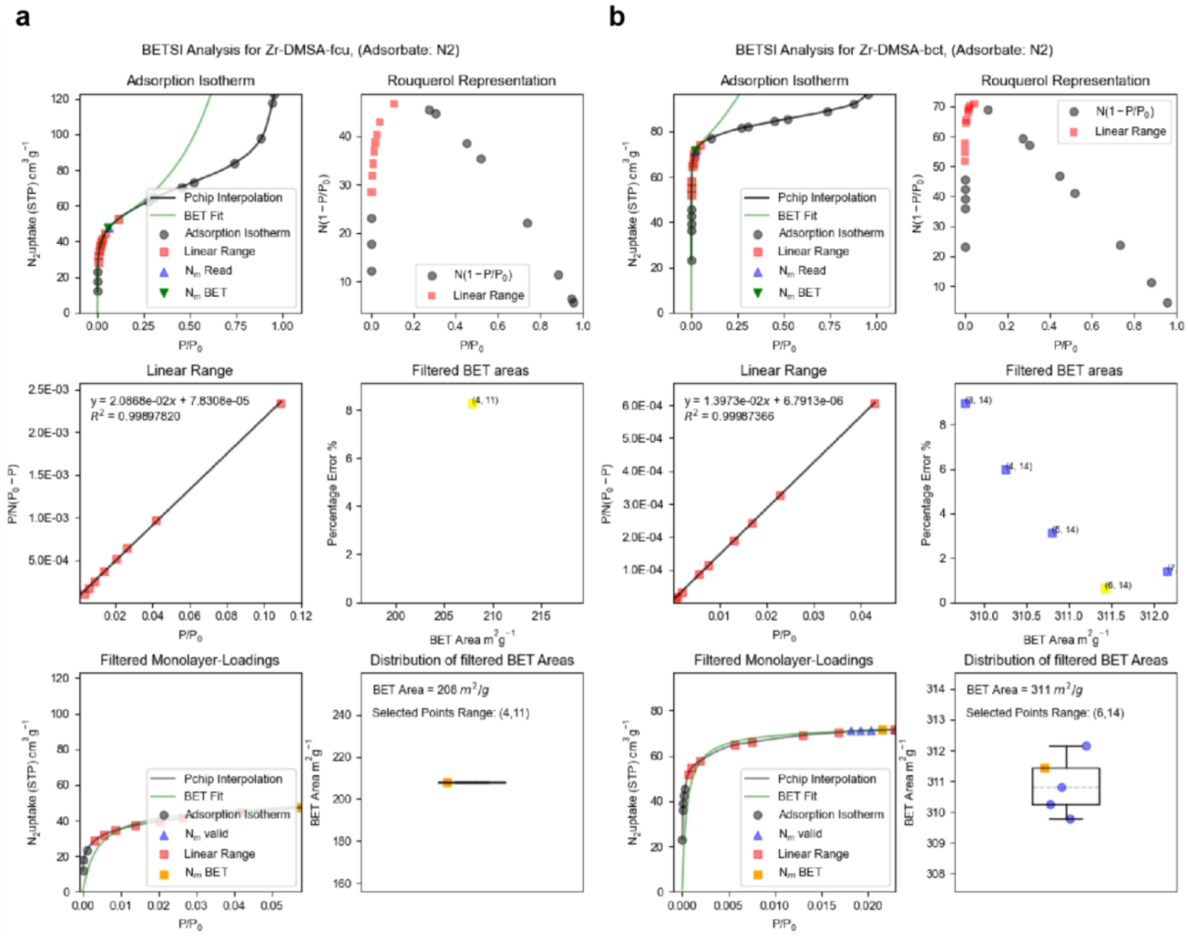

Figure S7 BETSI analysis output of a) Zr-DMSA-fcu b) Zr-DMSA-bct.

PDF analysis of Zr-DMSA-fcu

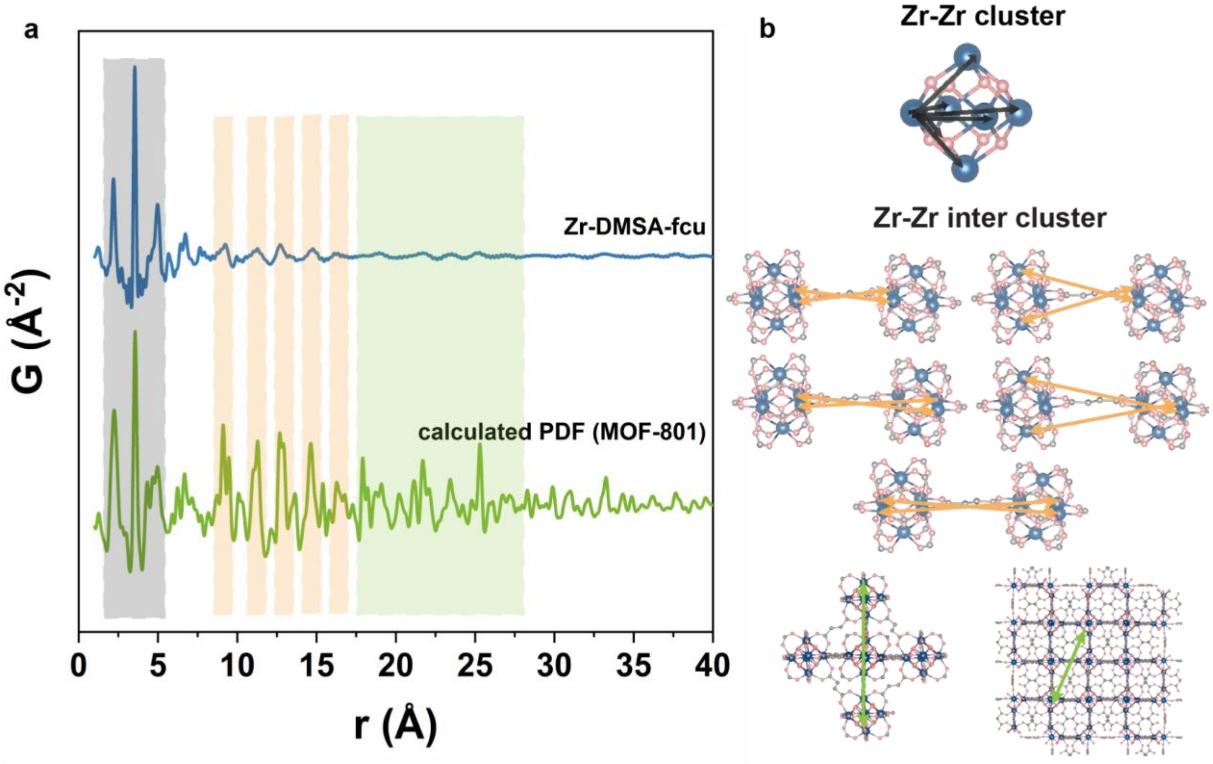

Figure S8 Overview of PDF analysis of Zr-DMSA-fcu. a) Shows the comparison of the calculated PDF of MOF-801 (green, COD: 4121459) and experiential PDF of Zr-DMSA-fcu (blue). Peaks from the Zr-oxo cluster are colored in black whereas higher r peaks pairs from inter cluster Zr-Zr pairs are colored in orange and green, respectively. b) Visualization of Zr–Zr distances in the structure of MOF-801.

CO_2 adsorption isotherm of all Zr-based MOFs

Figure S9 and **Figure S10** show the CO_2 adsorption isotherm for all Zr-based MOF in this study. Importantly, the Zr-SUC MOFs which are not accessible using N_2 both show similar adsorption of CO_2 as previously published.[1] Furthermore, Zr-SUC-bct showed a higher adsorption capacity for CO_2 which is in agreement with previous literature.[1] For DMSA based MOFs the Zr-DMSA-bct showed the highest CO_2 adsorption capacity. Langmuir fits for all materials are presented in **Figure S11**.

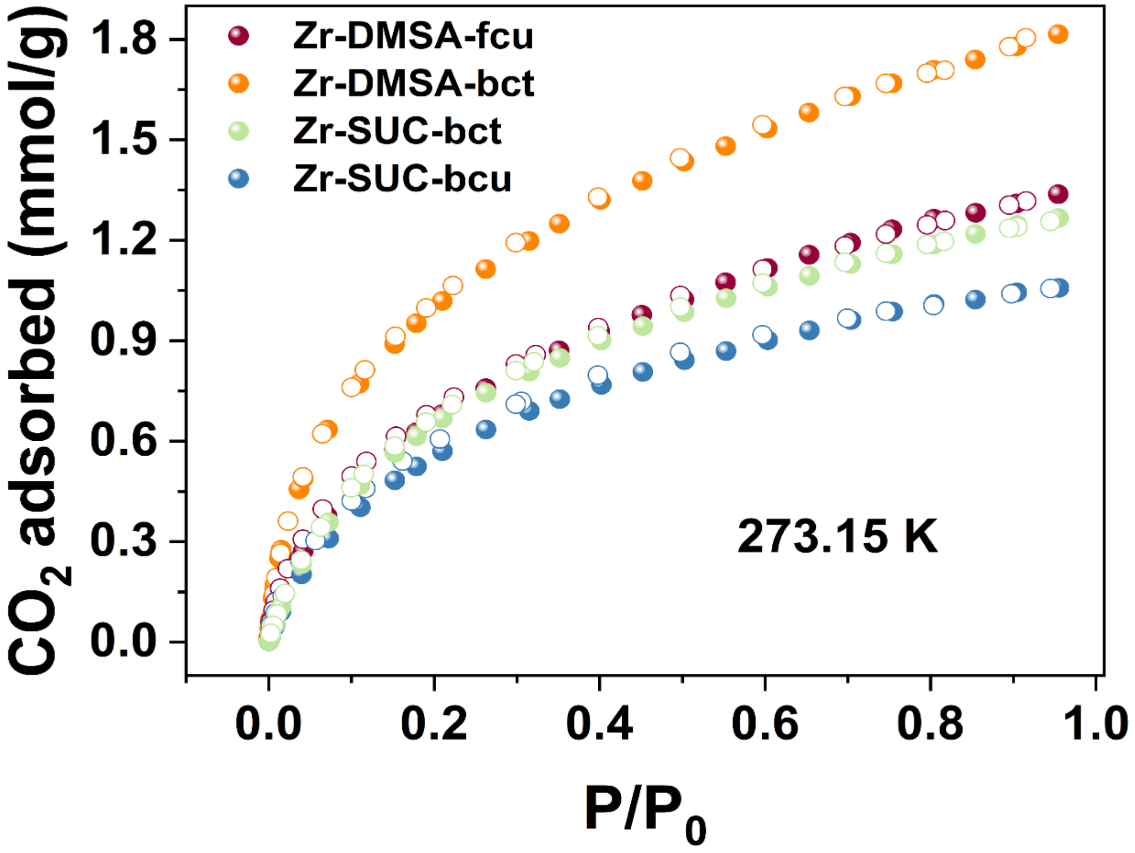

Figure S9 CO_2 adsorption isotherms for all studied MOFs measured 273.15 K.

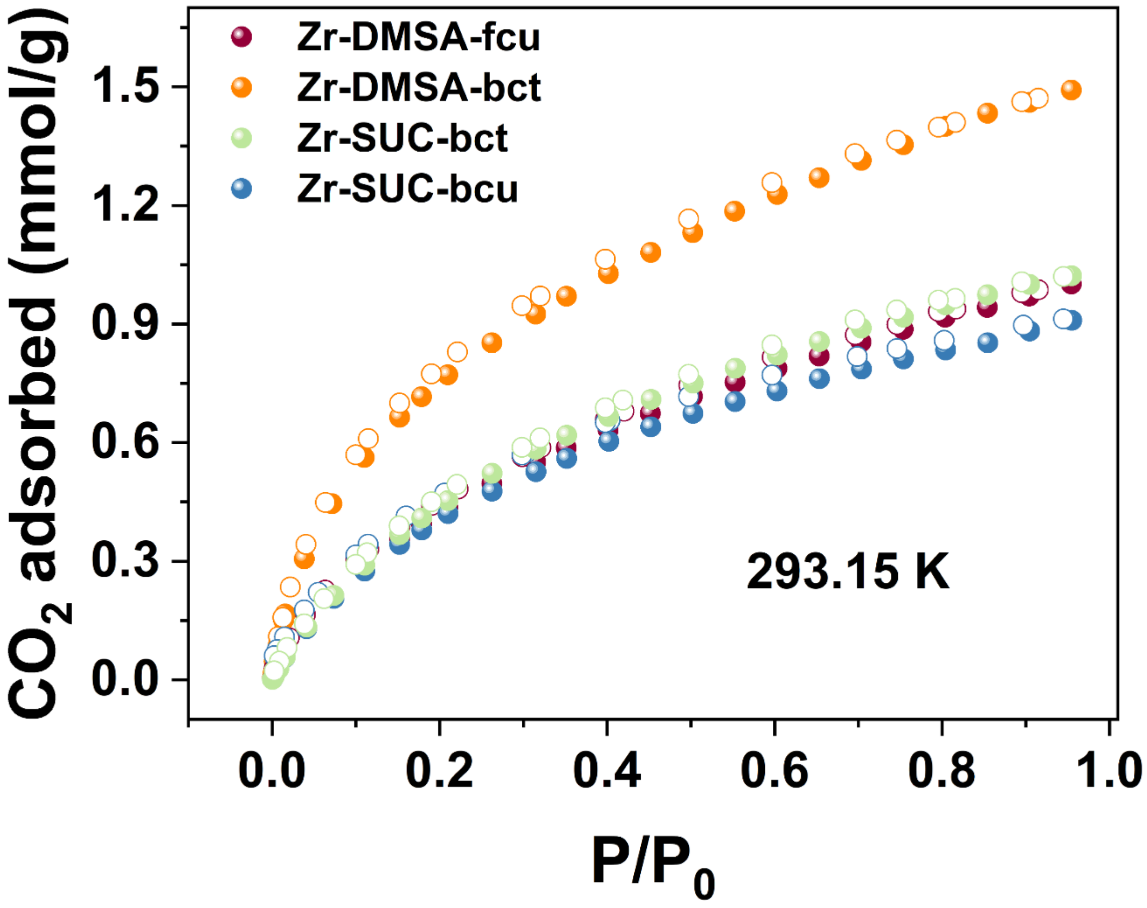

Figure S10 CO_2 adsorption isotherms for all studied MOFs measured 293.15 K.

Langmuir fits for all Zr-based MOFs at 273.15 K

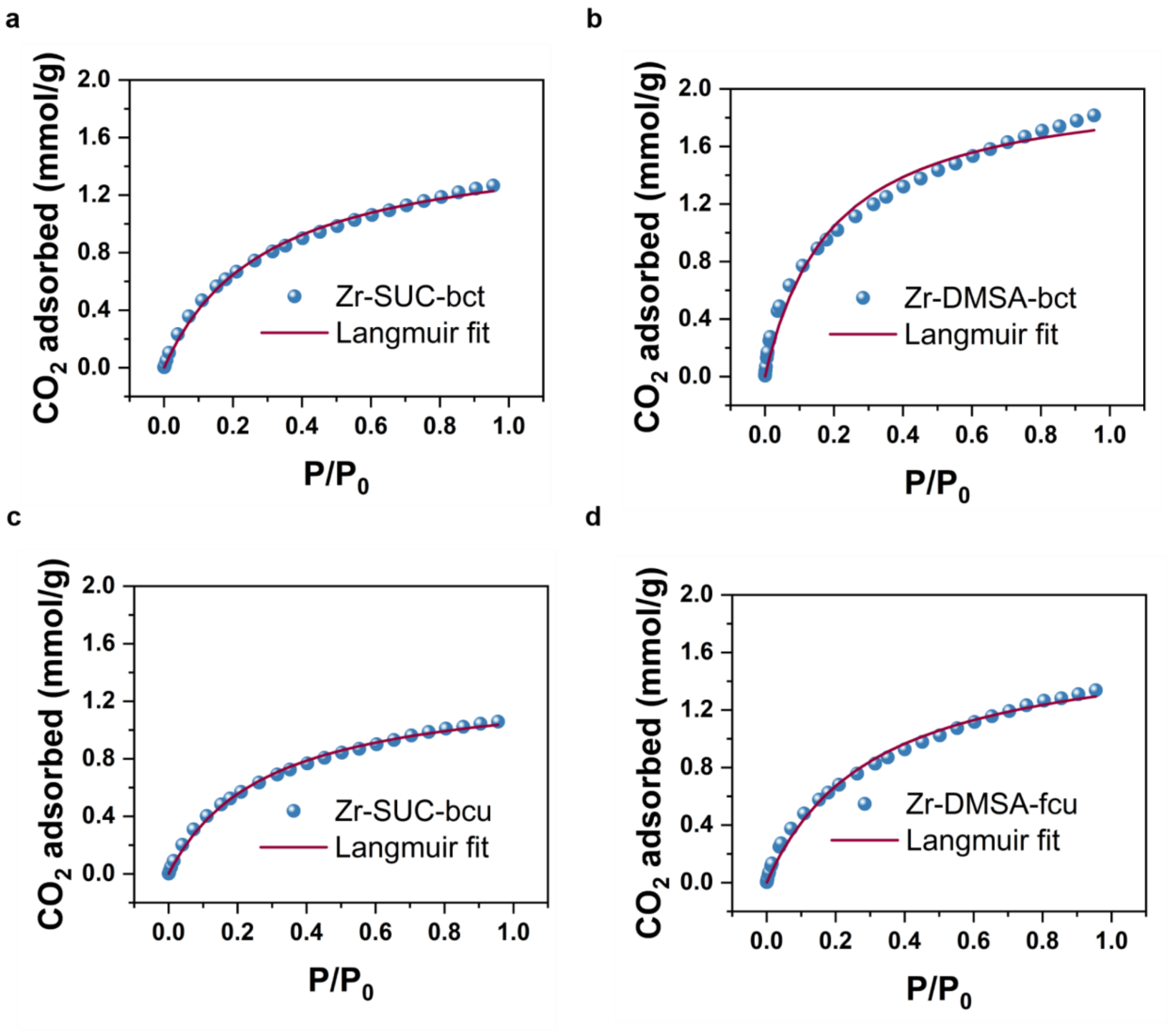

Figure S11 Langmuir fits of CO_2 adsorption isotherm at 273.15 K for all Zr-based MOFs; a) Zr-SUC-bct, b) Zr-DMSA-bct, c) Zr-SUC-bcu and d) Zr-DMSA-fcu.

TEM analysis of Zr-DMSA-fcu and Zr-DMSA-bct

Figure S12 and **S13** show TEM images and selected area electron diffraction patterns (SAEDP) recorded for Zr-DMSA-bct and Zr-DMSA-fcu, respectively.

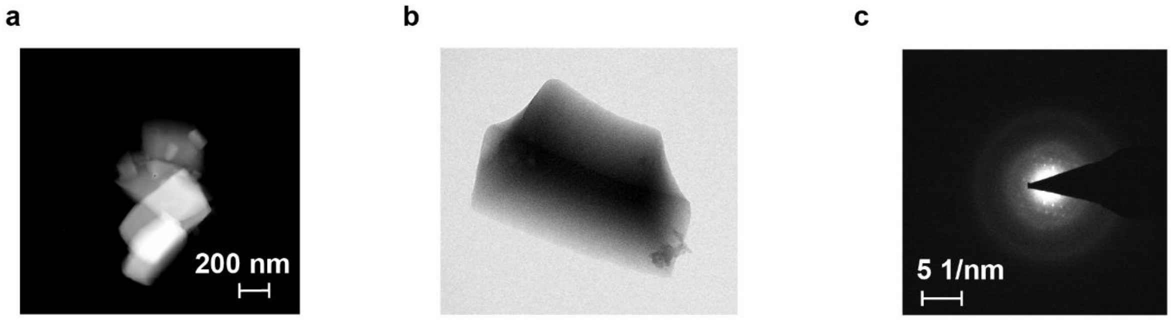

Figure S12 a-b) HAADF-STEM and TEM images of Zr-DMSA-bct. c) SAED pattern from one particle, showing long range ordering in the crystal.

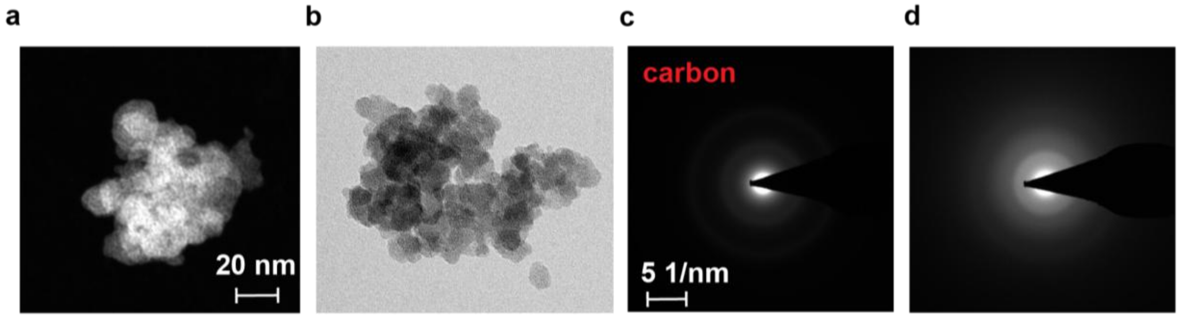

Figure S13 a-b) HAADF-STEM and TEM images of Zr-DMSA-fcu. SAED patterns acquired from c) carbon grid and d) agglomerated particles in b.

Elemental Analysis

Table S1 Elemental analysis results of the different Zr-based MOFs

sample	Carbon [%]	Hydrogen [%]	Nitrogen [%]	Sulfur [%]
Zr-SUC-bct	14.87 ± 0.05	2.92 ± 0.01	0	0
Zr-SUC-bcu	15.10 ± 0.03	2.34 ± 0.06	0	0
Zr-DMSA-bct	15.40 ± 0.20	2.50 ± 0.12	0	5.75 ± 0.07
Zr-DMSA-fcu	15.44 ± 0.11	2.16 ± 0.08	0	14.77 ± 0.10

Quantitative ssNMR analysis

SsNMR of Linker

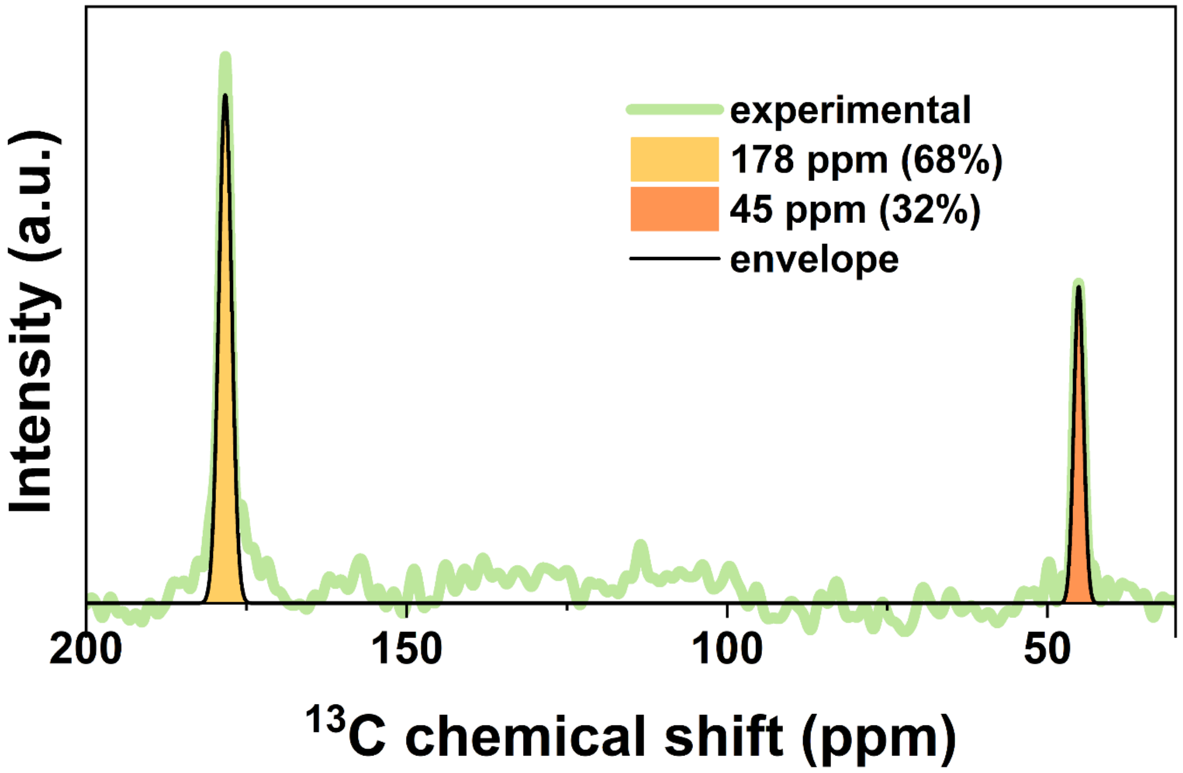

Figure S14 Fitting of ^{13}C multi-CP (multiple cross polarization) NMR spectra of DMSA.

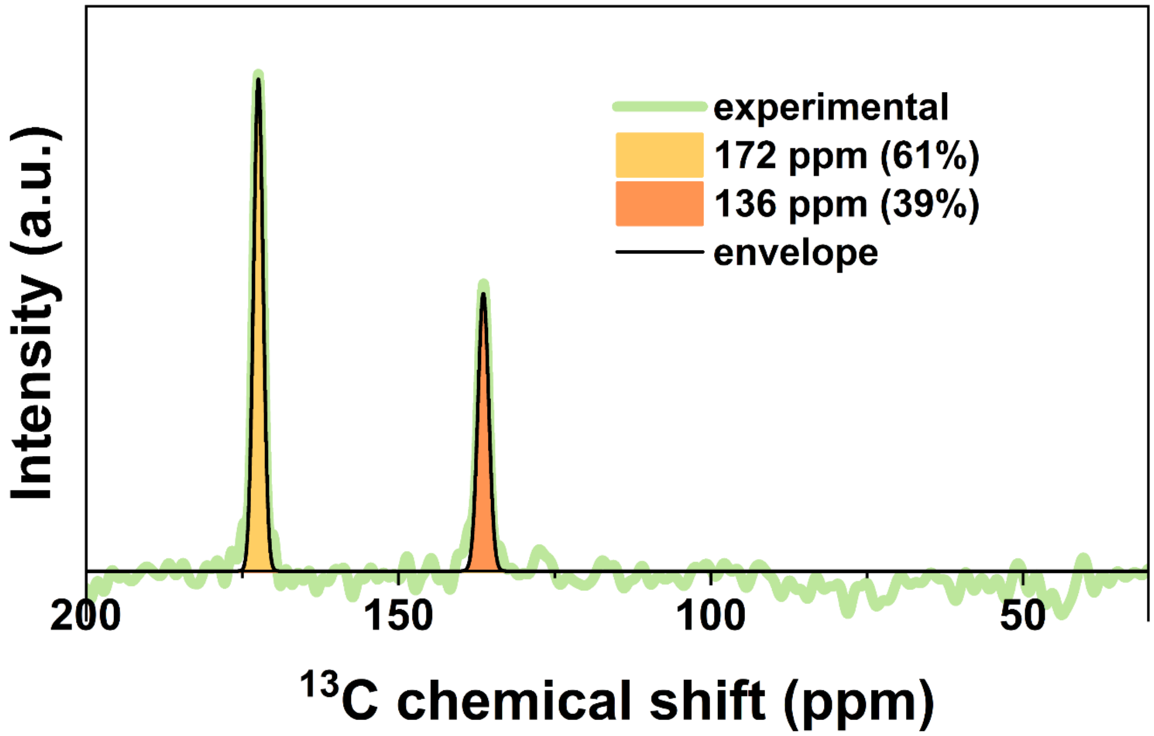

Figure S15 Fitting of ^{13}C multi-CP (multiple cross polarization) NMR spectra of fumaric acid.

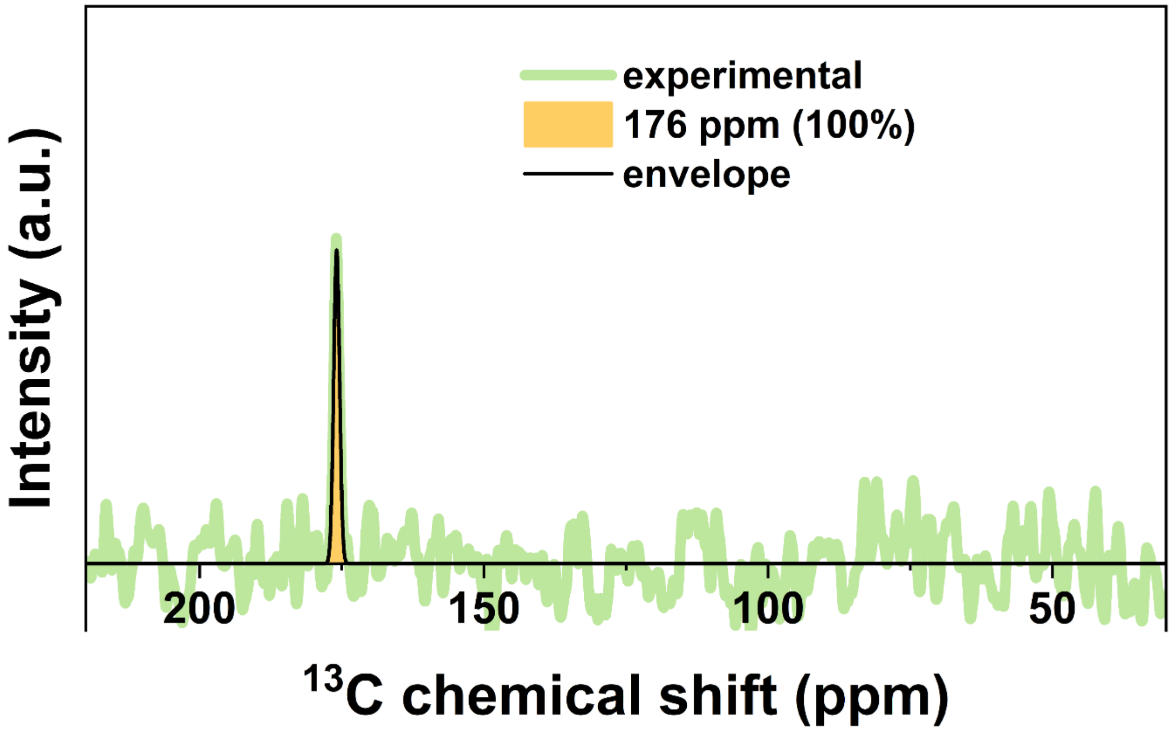

Figure S16 Fitting of ^{13}C multi-CP (multiple cross polarization) NMR spectra of sodium formate.

SsNMR of Zr-DMSA-bct and Zr-DMSA-fcu

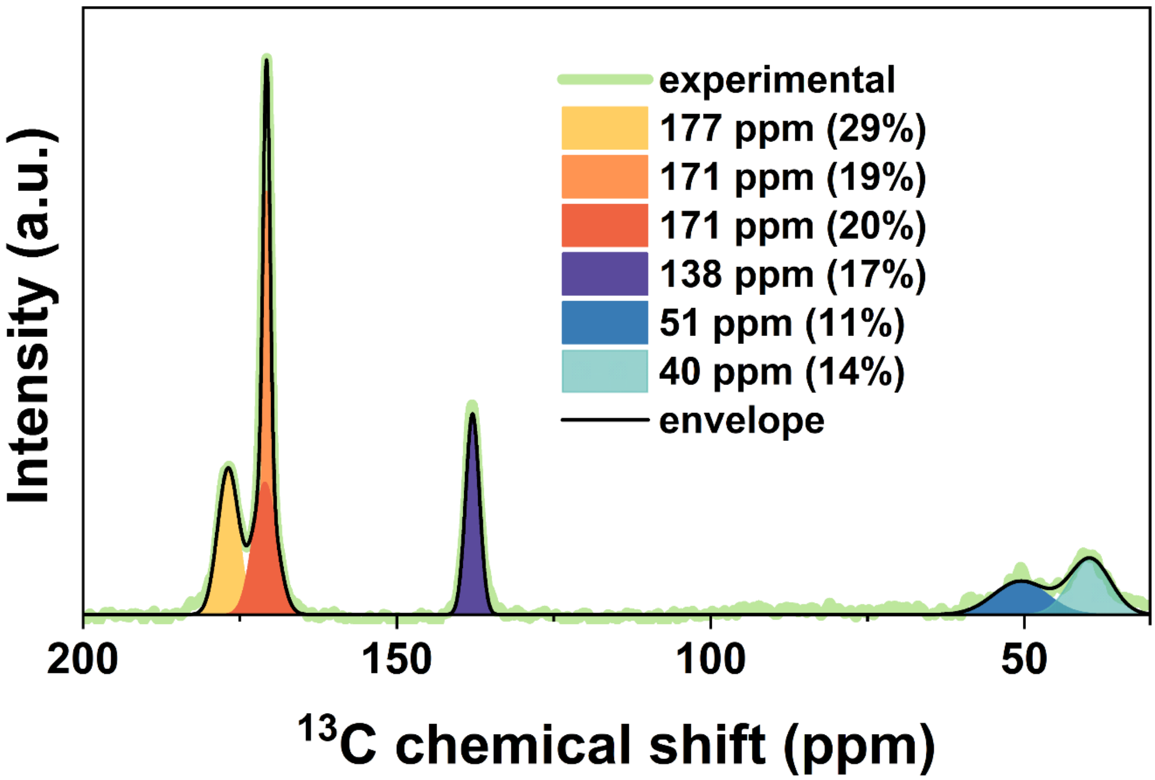

Figure S17 Fitting of ^{13}C multi-CP (multiple cross polarization) NMR spectra of Zr-DMSA-bct.

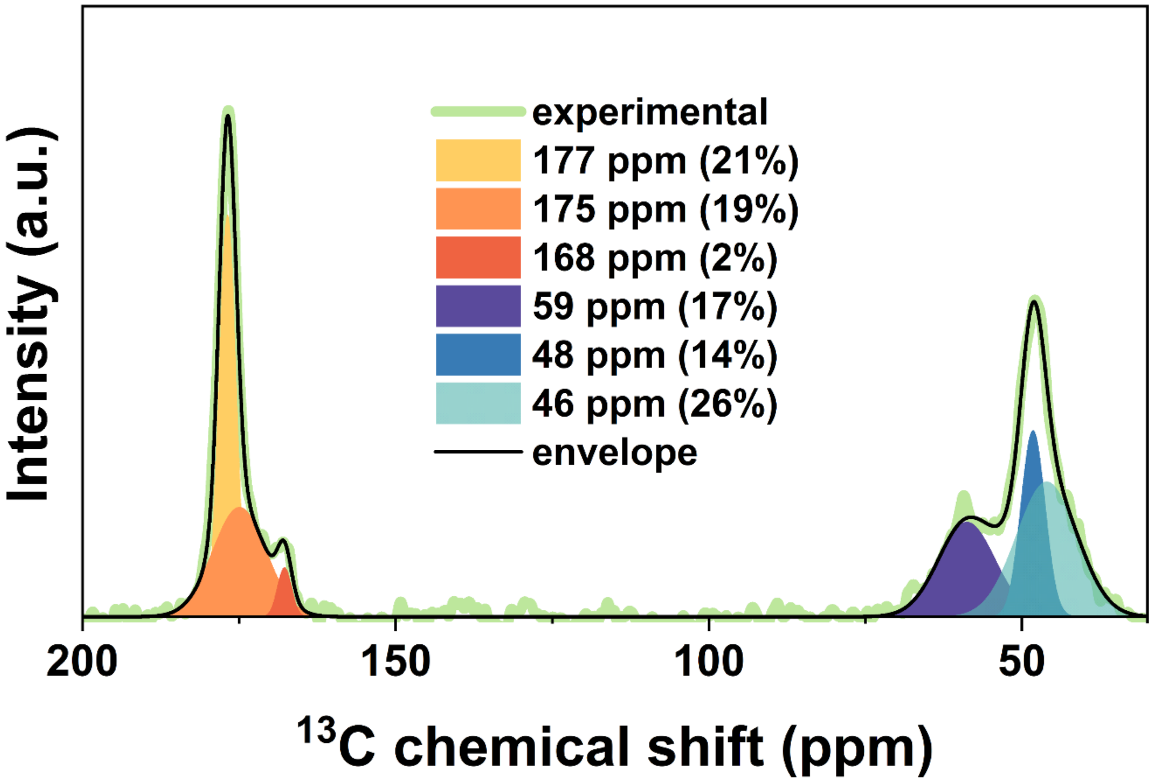

Figure S18 Fitting of ^{13}C multi-CP (multiple cross polarization) NMR spectra of Zr-DMSA-fcu.

Table S2 Chemical shift and relative abundance of carbon species in organic linkers obtained by fitting ^{13}C multiCP spectra of the different samples.

sample	carbonyl	Sp_2 carbons	C-SH	relative C-SH/sp_2 carbons (%)
DMSA	178 ppm (68%)	-	45 ppm (32%)	-
fumaric acid	172 ppm (61%)	136 ppm (39%)	-	-
sodium formate	176 ppm (100%)	-	-	-
Zr-DMSA-fcu	177 ppm (21%)<br>175 ppm (19%)<br>168 ppm (2%)	-	59 ppm (17%)<br>48 ppm (14%)<br>46 ppm (26%)	100:0
Zr-DMSA-bct	177 ppm (19%)<br>171 ppm (19%)<br>171 ppm (20%)	138 ppm (17%)	51 ppm (11%)<br>40 ppm (14%)	approx 60:40

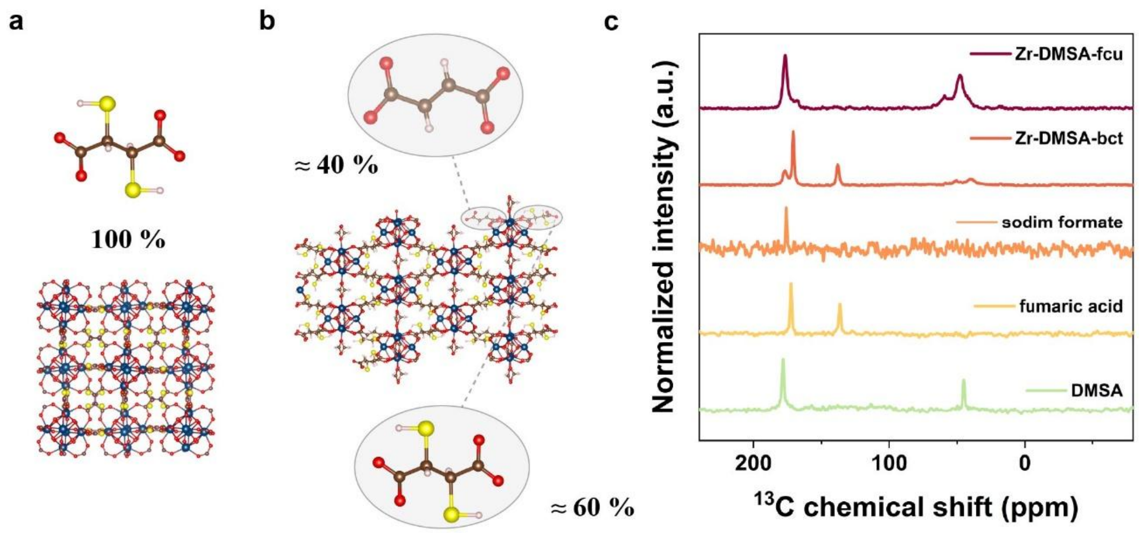

Figure S19 Overview of the ssNMR analysis of thiol-based MOFs, organic linkers and modulator; a) structural representation of Zr-DMSA-fcu, composed entirely of DMSA linkers (100%), b) structural representation of Zr-DMSA-bct, containing a 60:40 ratio of DMSA and fumarate linkers, c) ^{13}C multi-CP (multiple cross polarization) NMR spectra of Zr-DMSA-fcu (red), Zr-DMSA-bct (dark orange), and reference compounds: DMSA (green), fumaric acid (yellow), and sodium formate (orange).

STEM-EDXS analysis

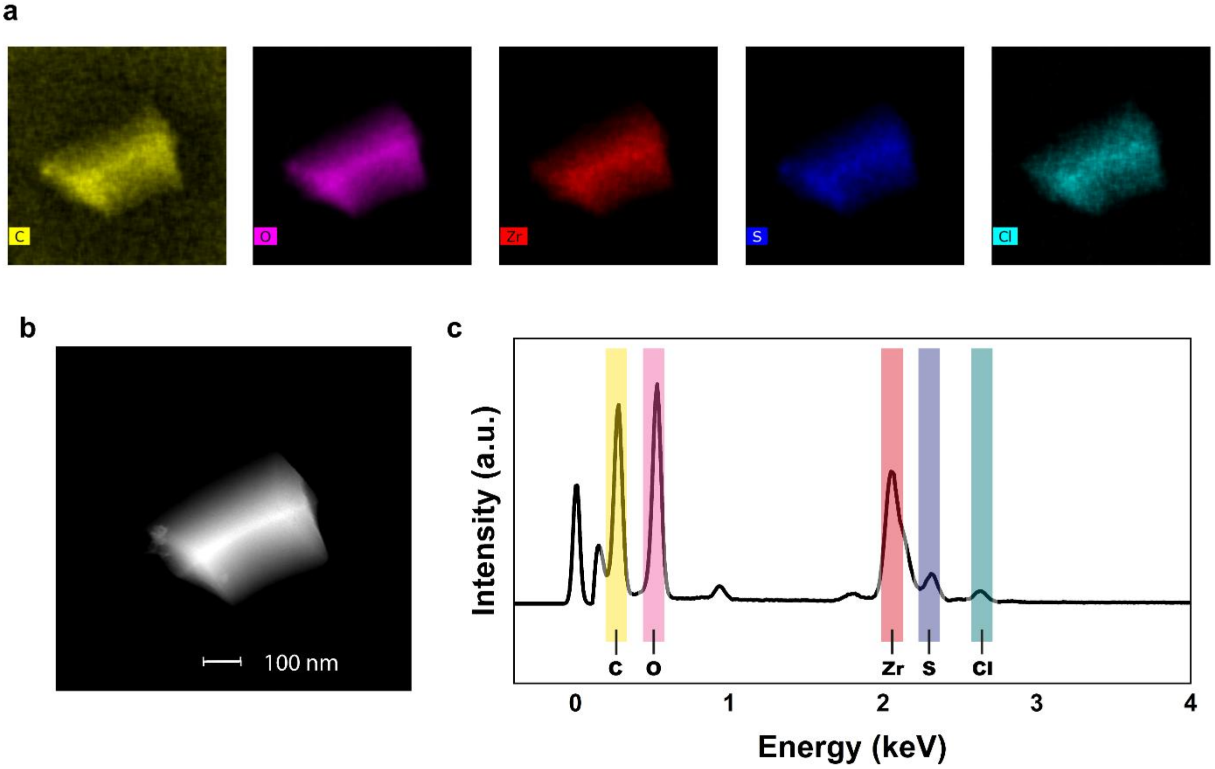

Figure S20 STEM-EDXS analysis of Zr-DMSA-bct particle; a) EDXS elemental maps and b) HAADF-STEM image of particle c) EDX spectrum of the measured sample.

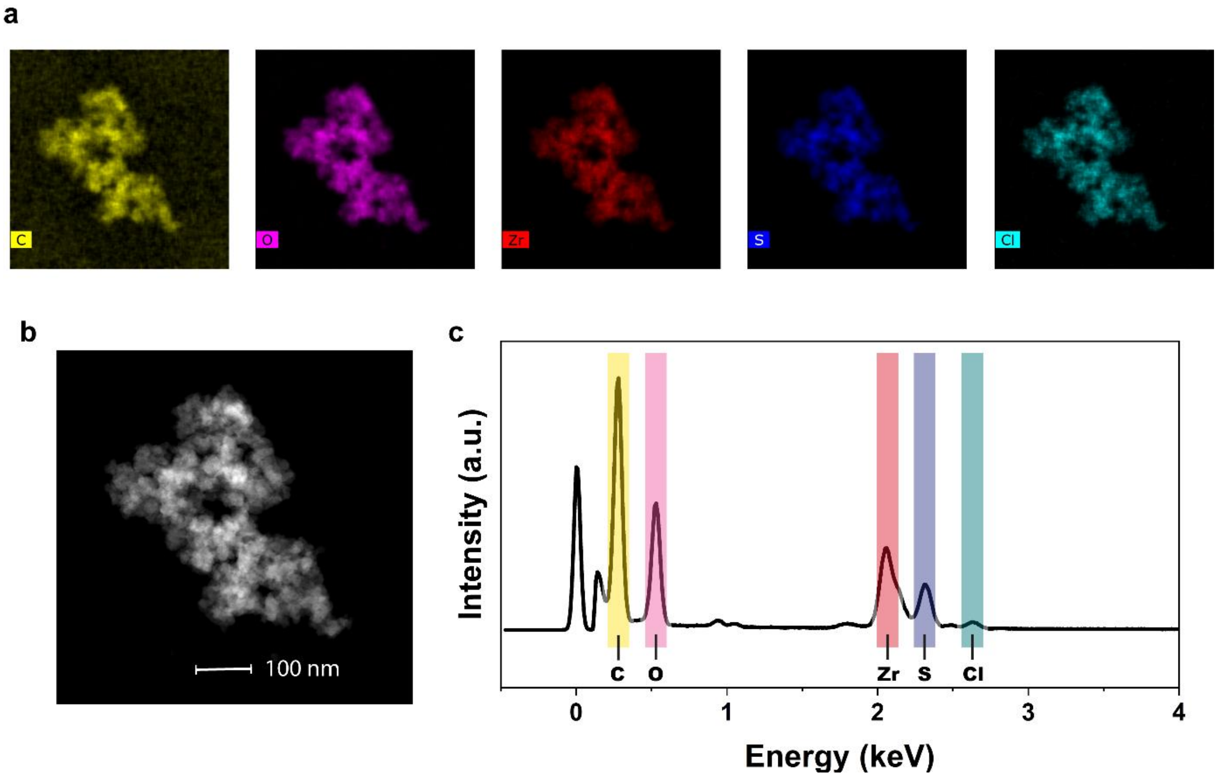

Figure S21 STEM-EDXS analysis of Zr-DMSA-fcu particles; a) EDXS elemental maps and b) HAADF image of particles, c) EDX spectrum of the measured sample.

Arsenic adsorption isotherms

Figure S22 and **S23** present the arsenic adsorption isotherms for both Zr-DMSA and both Zr-SUC MOFs, respectively. The isotherms were fitted using Langmuir and Freundlich model and the fitting parameter are presented in **Table S3** and **S4**, respectively.

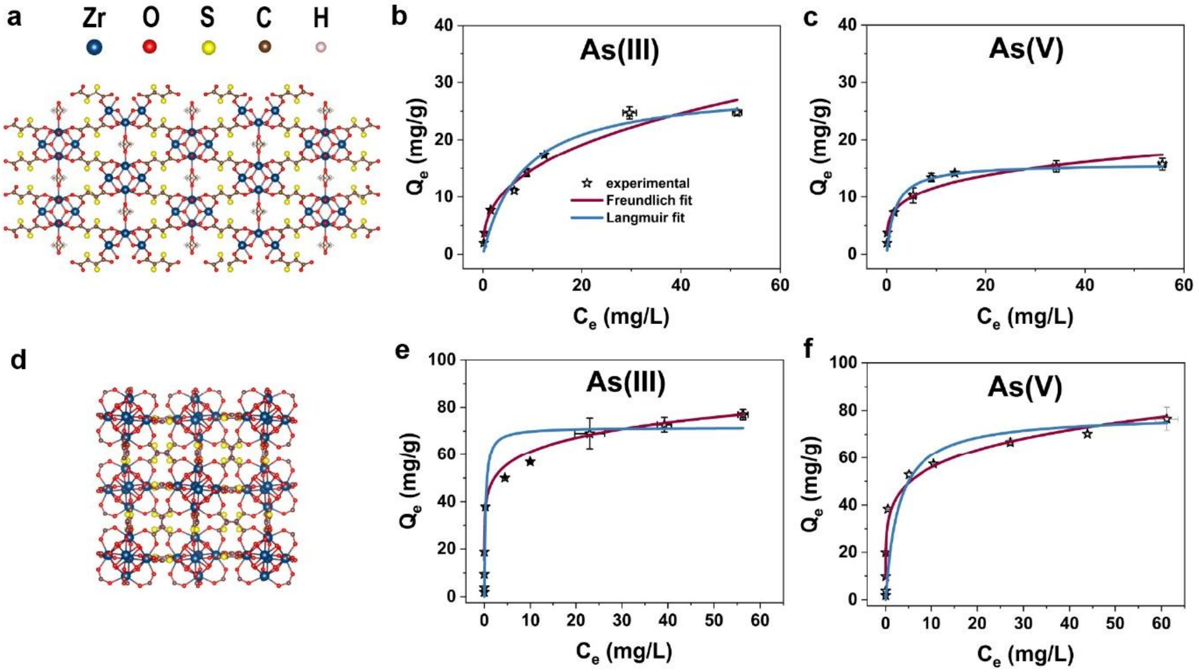

Figure S22 Arsenic isotherms for Zr-DMSA MOFs, a) Schematic representation of Zr-DMSA-bct with b) As(III) and c) As(V) isotherm. d) Schematic representation of Zr-DMSA-fcu with e) As(III) and f) As(V) adsorption isotherm. Conditions: Initial concentration arsenic (1-100 ppm), volume (10 mL), adsorbent dosage (0.5 g/L), adsorption time (24 h) and pH (7).

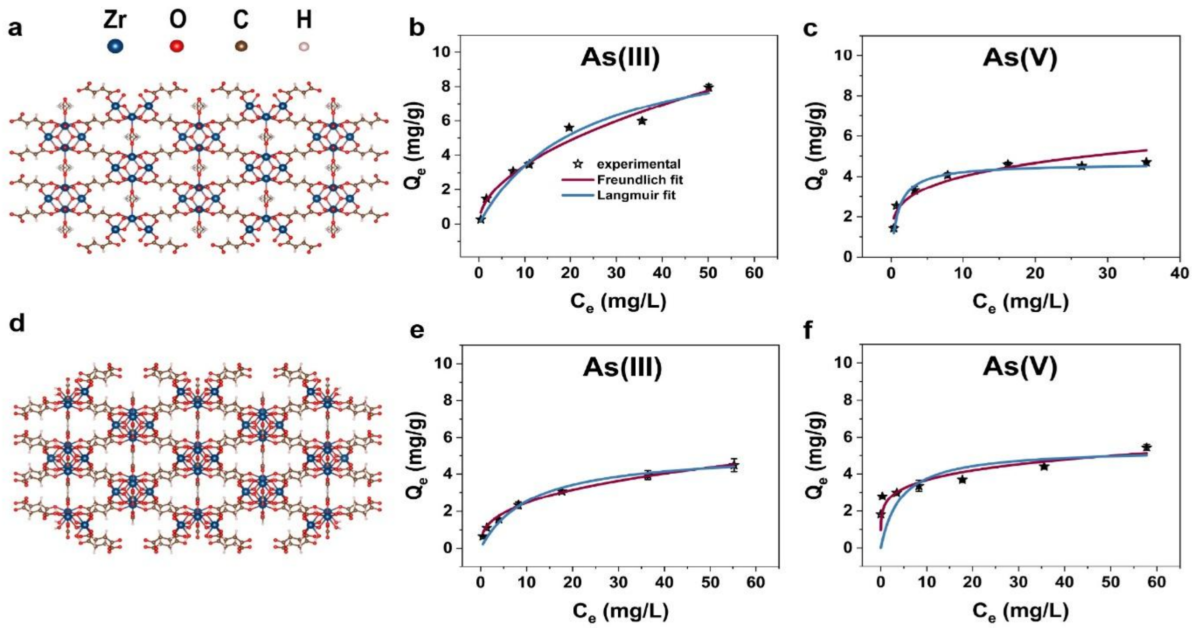

Figure S23 Arsenic isotherms for Zr-SUC MOFs, a) Schematic representation of Zr-SUC-bct with b) As(III) and c) As(V) isotherm. d) Schematic representation of Zr-SUC-bcu with e) As(III) and f) As(V) adsorption isotherm. Conditions: Initial concentration arsenic (1-60 ppm), volume (10 mL), adsorbent dosage (0.5 g/L), adsorption time (24 h) and pH (7).

Table S3 Fitting parameters from Freundlich model

Material	Arsenic speciation	K_F (L g^{-1})	1/n	R^2
Zr-DMSA-fcu	As(III)	45.05 ± 1.39	0.13 ± 0.01	0.97554
Zr-DMSA-fcu	As(V)	36.82 ± 2.13	0.18 ± 0.01	0.97619
Zr-DMSA-bct	As(III)	6.38 ± 0.78	0.36 ± 0.03	0.962068
Zr-DMSA-bct	As(V)	6.85 ± 0.72	0.23 ± 0.03	0.93373
Zr-SUC-bct	As(III)	1.04 ± 0.17	0.51 ± 0.04	0.98015
Zr-SUC-bct	As(V)	2.42 ± 0.21	0.21 ± 0.03	0.91392
Zr-SUC-bcu	As(III)	1.06 ± 0.05	0.36 ± 0.01	0.99637
Zr-SUC-bcu	As(V)	2.38 ± 0.28	0.18 ± 0.36	0.85657

Table S4 Fitting parameters from Langmuir model

Material	Arsenic speciation	q_m (mg/g)	b	R^2
Zr-DMSA-fcu	As(III)	71.62 ± 1.47	3.84 ± 2.00	0.59926
Zr-DMSA-fcu	As(V)	78.30 ± 1.77	0.35 ± 0.067	0.87741
Zr-DMSA-bct	As(III)	29.31 ± 2.82	0.12 ± 0.03	0.95271
Zr-DMSA-bct	As(V)	15.79 ± 0.94	0.57 ± 0.18	0.9474
Zr-SUC-bct	As(III)	11.04 ± 1.27	0.04 ± 0.01	0.96255
Zr-SUC-bct	As(V)	4.65 ± 0.17	0.99 ± 0.20	0.96677
Zr-SUC-bcu	As(III)	5.27 ± 0.21	0.09 ± 0.01	0.98261
Zr-SUC-bcu	As(V)	5.38 ± 0.87	0.23 ± 0.16	0.26683

Calculation Qe at 500ppb

The equilibrium uptake Qe_{500} at C_e = 500 ppb was calculated in order to compare the arsenic uptake at low concentration between the different materials. The presented values in **Table 1** are given with an 80% confidence level. For alpha = 0.2, the critical value was calculated using Python's SciPy library (scipy.stats.t.ppf). The upper and lower bounds are calculated as follows:

$$upper\ /\ lower\ bound = Qe_{500} \pm (critical\ value * SE)$$

Where SE is the standard error using the residual sum of squares (RSS) and the degree of freedom N:

$$RSS = \sum (Qe_{exp} - Qe_{fit})^2$$

$$N = (number\ of\ data\ points) - 2$$

$$SE = \sqrt{\frac{RSS}{N}}$$

Table S5 Data from As(III) adsorption experiment with Zr-DMSA-fcu

Sample	Ce (mg/L)	Ce error	Qe (mg/g)	Qe error
Zr-DMSA-fcu	3.1×10^{-4}	1.0×10^{-4}	1.85805	2.1×10^{-4}
Zr-DMSA-fcu	2.8×10^{-4}	2.6×10^{-4}	3.77805	5.3×10^{-4}
Zr-DMSA-fcu	1.35×10^{-3}	1.02×10^{-3}	9.45408	2.05×10^{-3}
Zr-DMSA-fcu	1.68×10^{-3}	1.2×10^{-4}	18.72637	2.5×10^{-4}
Zr-DMSA-fcu	0.241	0.138	37.915	0.277
Zr-DMSA-fcu	4.466	0.048	50.054	0.096
Zr-DMSA-fcu	9.940	0.167	56.835	0.334
Zr-DMSA-fcu	22.993	3.242	69.005	6.484
Zr-DMSA-fcu	39.216	1.542	72.753	3.085
Zr-DMSA-fcu	56.270	1.067	76.961	2.135

Table S6 Data from As(V) adsorption experiment with Zr-DMSA-fcu

Sample	Ce (mg/L)	Ce error	Qe (mg/g)	Qe error
Zr-DMSA-fcu	4.6×10^{-4}	3.29×10^{-4}	1.83733	6.5×10^{-4}
Zr-DMSA-fcu	1.22×10^{-3}	5.0×10^{-4}	3.7966	1.02×10^{-3}
Zr-DMSA-fcu	1.83×10^{-3}	2.0×10^{-4}	9.80034	4.1×10^{-4}
Zr-DMSA-fcu	0.011	0.003	19.871	0.006
Zr-DMSA-fcu	0.483	0.012	38.351	0.025
Zr-DMSA-fcu	5.082	0.582	52.785	1.165
Zr-DMSA-fcu	10.501	0.017	57.368	0.578
Zr-DMSA-fcu	27.165	0.763	66.667	1.526
Zr-DMSA-fcu	43.866	0.298	70.234	0.597
Zr-DMSA-fcu	61.135	2.404	76.546	4.809

Table S7 Data from As(III) adsorption experiment with Zr-DMSA-bct

Sample	Ce (mg/L)	Ce error	Qe (mg/g)	Qe error
Zr-DMSA-bct	0.1425	0.007	1.965	0.015
Zr-DMSA-bct	0.33	0.005	3.69	0.01
Zr-DMSA-bct	1.525	0.03	7.71	0.06
Zr-DMSA-bct	6.28	0.32	11.064	0.445
Zr-DMSA-bct	8.82	0.345	14.26	0.69
Zr-DMSA-bct	12.405	0.15	17.355	0.214
Zr-DMSA-bct	29.64	1.33	24.708	1.032
Zr-DMSA-bct	51.3	0.925	24.759	0.532

Table S8 Data from As(V) adsorption experiment with Zr-DMSA-bct

Sample	Ce (mg/L)	Ce error	Qe (mg/g)	Qe error
Zr-DMSA-bct	0.0775	0.0125	1.955	0.025
Zr-DMSA-bct	0.16	0.07	3.75	0.14
Zr-DMSA-bct	1.495	0.185	7.3	0.37
Zr-DMSA-bct	5.32	0.655	10.24	1.31
Zr-DMSA-bct	8.985	0.38	13.39	0.76
Zr-DMSA-bct	13.8125	0.0625	14.185	0.125
Zr-DMSA-bct	34.2125	0.7025	15.34617	0.97383
Zr-DMSA-bct	55.6375	0.7325	15.71574	1.02426

Table S9 Data from As(III) adsorption experiment with Zr-SUC-bct

Sample	Ce (mg/L)	Ce error	Qe (mg/g)	Qe error
Zr-SUC-bct	0.413	0.012	0.272	0.025
Zr-SUC-bct	1.623	0.022	1.469	0.044
Zr-SUC-bct	7.444	0.044	3.084	0.088
Zr-SUC-bct	10.937	0.071	3.479	0.143
Zr-SUC-bct	19.720	0.019	5.603	0.038
Zr-SUC-bct	35.600	0.054	5.984	0.109
Zr-SUC-bct	50.099	0.091	7.952	0.182

Table S10 Data from As(V) adsorption experiment with Zr-SUC-bct

Sample	Ce (mg/L)	Ce error	Qe (mg/g)	Qe error
Zr-SUC-bct	0.347	0.060	1.429	0.120
Zr-SUC-bct	0.718	0.004	2.546	0.009
Zr-SUC-bct	3.240	0.100	3.322	0.200
Zr-SUC-bct	7.867	0.062	4.078	0.125
Zr-SUC-bct	16.207	0.051	4.595	0.102
Zr-SUC-bct	26.440	0.030	4.520	0.067
Zr-SUC-bct	35.359	0.077	4.706	0.020

Table S11 Data from As(III) adsorption experiment with Zr-SUC-bcu

Sample	Ce (mg/L)	Ce error	Qe (mg/g)	Qe error
Zr-SUC-bcu	0.485	0.019	0.646	0.038
Zr-SUC-bcu	1.334	0.004	1.109	0.009
Zr-SUC-bcu	3.945	0.0192	1.565	0.038
Zr-SUC-bcu	8.207	0.0792	2.314	0.1585
Zr-SUC-bcu	17.667	0.0382	3.063	0.076
Zr-SUC-bcu	36.387	0.123	3.942	0.246
Zr-SUC-bcu	55.253	0.173	4.486	0.346

Table S12 Data from As(V) adsorption experiment with Zr-SUC-bcu

Sample	Ce (mg/L)	Ce error	Qe (mg/g)	Qe error
Zr-SUC-bcu	0.008	0.001	1.820	0.002
Zr-SUC-bcu	0.343	0.050	2.775	0.064
Zr-SUC-bcu	3.451	0.008	2.980	0.033
Zr-SUC-bcu	8.268	0.149	3.356	0.298
Zr-SUC-bcu	17.814	0.031	3.690	0.063
Zr-SUC-bcu	35.570	0.050	4.400	0.071
Zr-SUC-bcu	57.775	0.085	5.446	0.170

Kinetic parameters

Kinetic parameters were fitted using a pseudo second order model (PSO).

Table S13 Parameters obtained by fitting the pseudo-second-order-model

Material	Arsenic speciation	k_2 (g (mg min)$^{-1}$	q_e (mg g^{-1})	R^2
Zr-DMSA-fcu	As(III)	$3.0*10^{-3}$	43.46	0.998
Zr-DMSA-fcu	As(V)	$3.3*10^{-3}$	41.80	0.997

Pore size of Zr-SUC-bct/bcu

Table S14 Overview pore size materials

material	method	Pore size	Reference
Zr-SUC-bct	PSD from DFT-optimized structure	~ 4.5 Å	[1]
Zr-SUC-bcu	PSD from DFT-optimized structure	~ 4.5 Å	[1]

Thermochemical radii of arsenic and phosphate

Table S15 Overview thermochemical radii of arsenic and phosphate

molecule	method	r (Å)	Reference
$H_2AsO_4^-$	calculation	2.27 ± 0.19	[14]
$H_2PO_4^-$	calculation	2.13 ± 0.19	[14]

Zeta-potential measurement

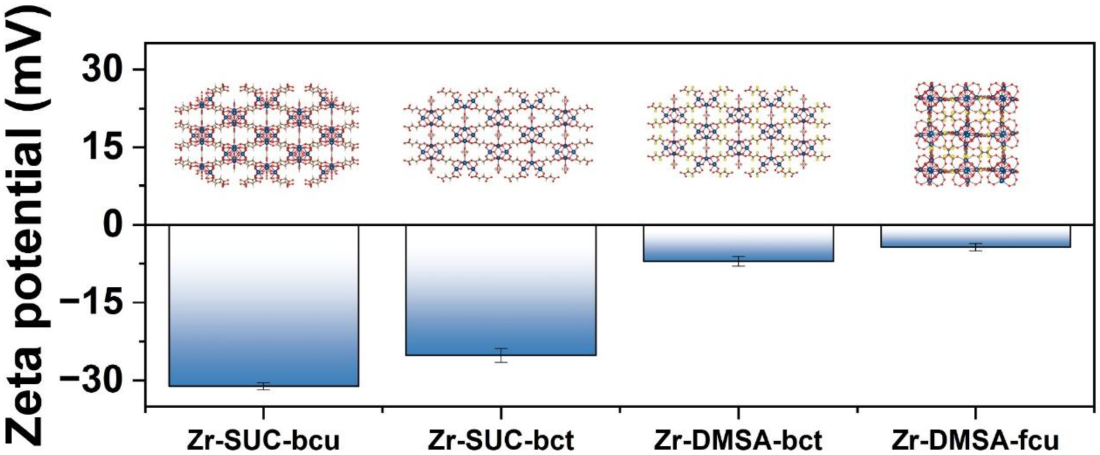

Figure S24 Zeta potential measured in Milli-Q water (pH = 7) at 25°C.

Phosphate selectivity

The selectivity of Zr-DMSA-fcu for phosphate was tested for both As(III) and As(V) and compared Zr-SUC-bct and benchmark material Zr-BDC. The materials were all tested by batch adsorption experiment with 1:1 and 1:10 As:HPO_4^{2-} concentration ratio. Complementary to the **Figure 8** in the manuscript, **Figure S25** shows the calculated Q_e values for selectivity experiments conducted for As(III) and As(V).

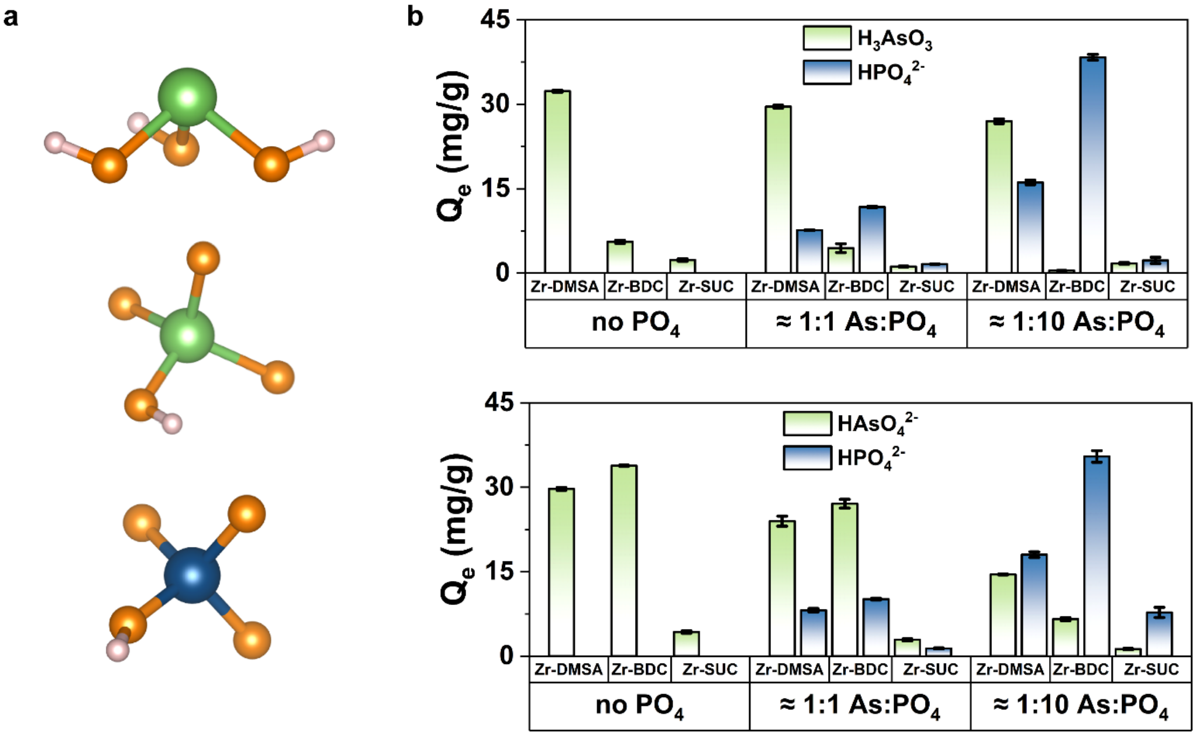

Figure S25 Competitive arsenic/phosphate batch adsorption experiments for Zr-DMSA, Zr-BDC and Zr-SUC-bct in aqueous solution containing 1:1 and 1:10 arsenic:phosphate concentration; a) Structural representation for arsenic and phosphate species, b) As(III) (top) and As(V) (bottom) competitive batch adsorption experiments. Conditions: initial arsenic concentration 10 ppm, pH = 7, adsorption time 24h, adsorption dosage 0.25 g/L. c) calculated selectivity coefficient for presented batch adsorption experiments for As(III) and d) for As(V).

K_d values arsenic/phosphate

Table S16 calculated distribution coefficient K_d values for As(III), P and HPO_4^{2-} selectivity

material	Initial As conc. (ppm)	Initial P / HPO_4^{2-} conc. (ppm)	K_d As (x10^3) (mL g^{-1})	K_d P (x10^3) (mL g^{-1})	$S_{As/P}$	$S_{As/HPO42-}$
Zr-DMSA-fcu	9	3 / 9	21.71 ± 1.11	6.90 ± 0.11	3.14 ± 0.12	3.14 ± 0.12
Zr-DMSA-fcu	9	30 / 90	12.49 ± 0.77	0.59 ± 0.01	20.96 ± 0.76	20.96 ± 0.76
Zr-BDC	9	3 / 9	0.44 ± 0.00	192.45 ± 93.97	0.002 ± 0.001	0.002 ± 0.001
Zr-BDC	9	30 / 90	0.051 ± 0.00	1.78 ± 0.03	0.028 ± 0.001	0.028 ± 0.001
Zr-SUC-bct	9	3 / 9	0.13 ± 0.00	0.61 ± 0.01	0.223 ± 0.007	0.223 ± 0.007
Zr-SUC-bct	9	30 / 90	0.10 ± 0.01	0.073 ± 0.01	1.51 ± 0.28	1.51 ± 0.28

Table S17 calculated distribution coefficient (K_d) values for As(V), P and HPO_4^{2-} selectivity

material	Initial As conc. (ppm)	Initial P / HPO_4^{2-} conc. (ppm)	K_d As (x10^3) (mL g^{-1})	K_d P (x10^3) (mL g^{-1})	$S_{As/P}$	$S_{As/ HPO42-}$
Zr-DMSA-fcu	9	3 / 9	22.54 ± 2.57	9.25 ± 1.21	2.43 ± 0.06	2.43 ± 0.06
Zr-DMSA-fcu	9	30 / 90	7.53 ± 0.08	0.70 ± 0.02	10.76 ± 0.45	10.76 ± 0.45
Zr-BDC	9	3 / 9	34.50 ± 4.09	25.72 ± 2.91	1.34 ± 0.02	1.34 ± 0.02
Zr-BDC	9	30 / 90	2.49 ± 0.12	1.65 ± 0.06	1.50 ± 0.01	1.50 ± 0.01
Zr-SUC-bct	9	3 / 9	0.97 ± 0.06	0.53 ± 0.03	1.83 ± 0.14	1.83 ± 0.14
Zr-SUC-bct	9	30 / 90	0.40 ± 0.03	0.27 ± 0.03	1.48 ± 0.07	1.48 ± 0.07

Le Bail fitting for MOFs

Table S18 Lattice parameters of all Zr-MOFs obtained by Le Bail profile fitting

Compound	Emission profile (Å)	a [Å]	b [Å]	c [Å]	space group	Rwp	GoF
Zr-SUC-bct	0.24486[a]	10.0454(6)	11.8458(7)	19.9164(3)	Imm2 (44)	8.45	1.40
Zr-SUC-bcu	1.540596[b]	11.1396(4)	11.1396(4)	19.7593(8)	P-4n2 (118)	6.58	0.11
Zr-DMSA-bct	0.24486[a]	10.0275(2)	12.0363(4)	19.7274(5)	Imm2 (44)	5.71	0.75
Zr-DMSA-fcu	1.540596[b]	17.756(1)	17.756(1)	17.756(1)	Pn-3:2 (201)	2.89	1.18

a) synchrotron radiation b) lab based Cu-source

Le bail fit Zr-DMSA-bct

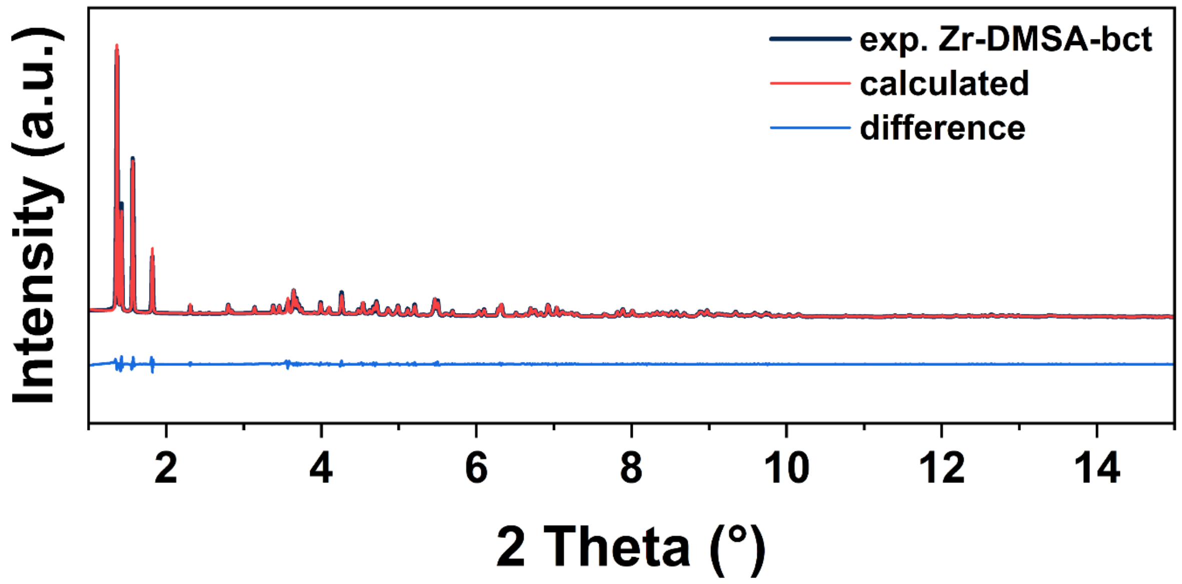

Figure S26 le bail fit for Zr-DMSA-bct.

Le bail fit Zr-SUC-bct

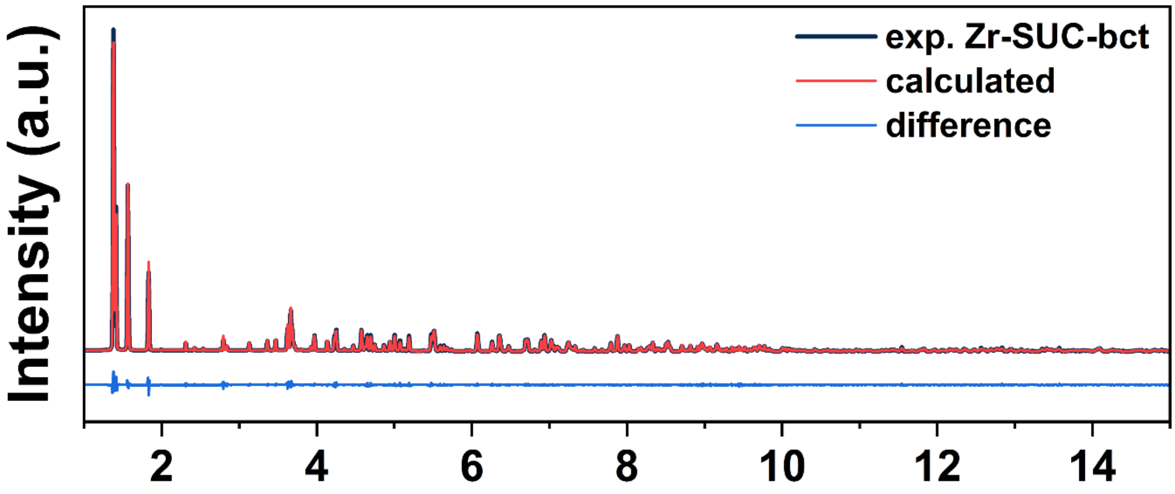

Figure S27 le bail fit for Zr-SUC-bct.

Le Bail fit Zr-DMSA-fcu

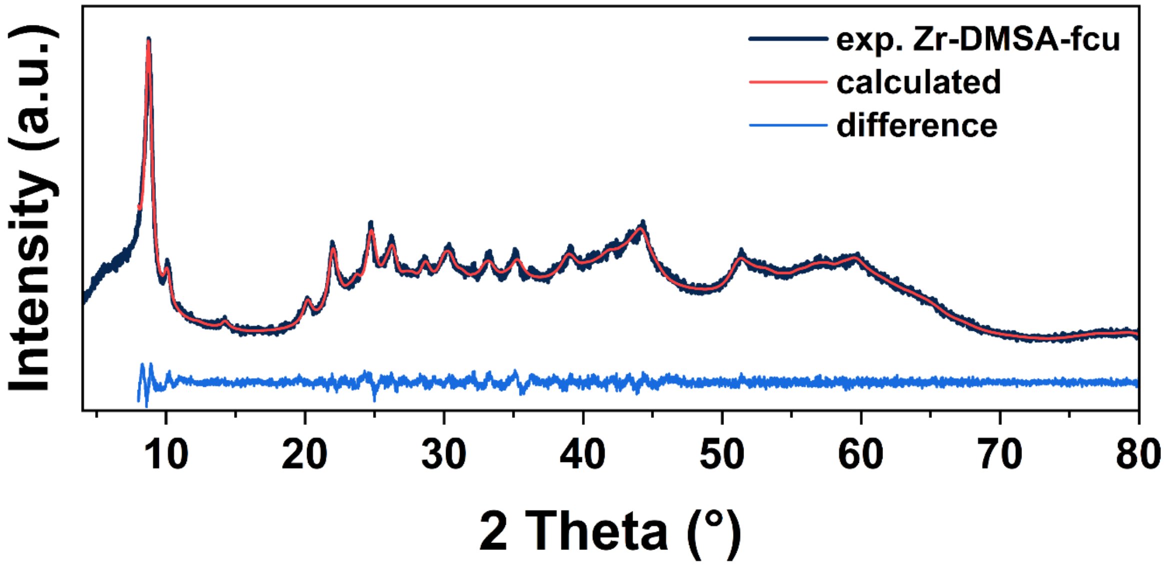

Figure S28 le bail fit for Zr-DMSA-fcu.

Le bail fit Zr-SUC-bcu

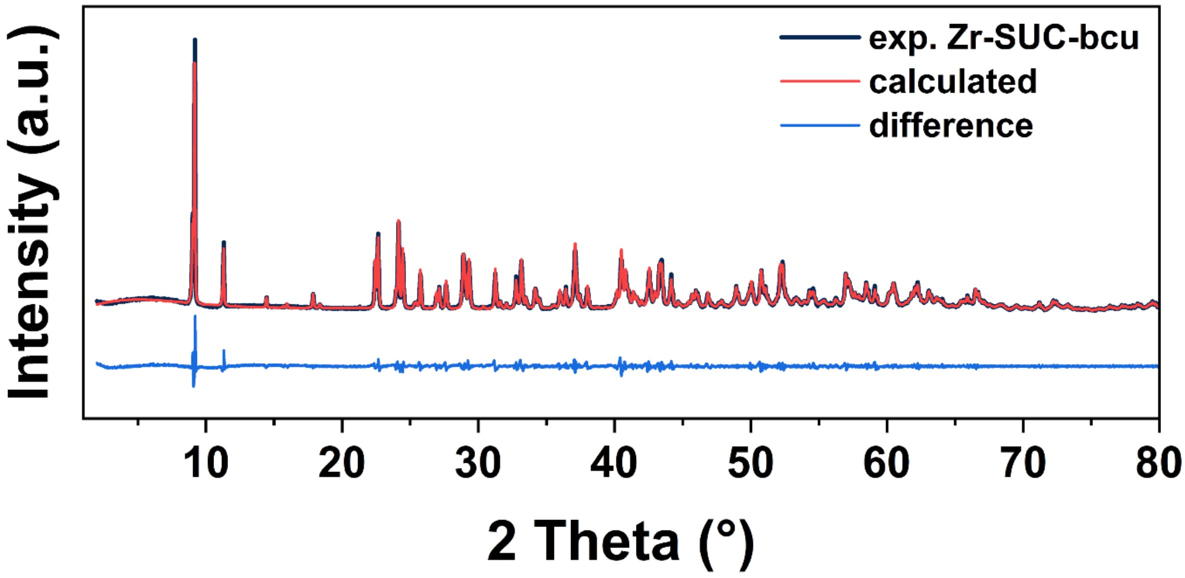

Figure S29 le Bail fit for Zr-SUC-bcu.

Table S19 Redox potential arsenate and phosphate

Compound	chemical reaction	Standard Reduction potential E° (V)	Reference
H_3AsO_4	$H_3AsO_4 + 2H^+ + 2\ e^- \rightarrow HAsO_2 + 2H_2O$	0.560	[15]
H_3PO_4	$H_3PO_4 + 2H^+ + 2\ e^- \rightarrow H_3PO_3 + 2H_2O$	- 0.267	[15]

First-Principles Density Functional Theory (DFT) Calculations

Arsenic adsorption model

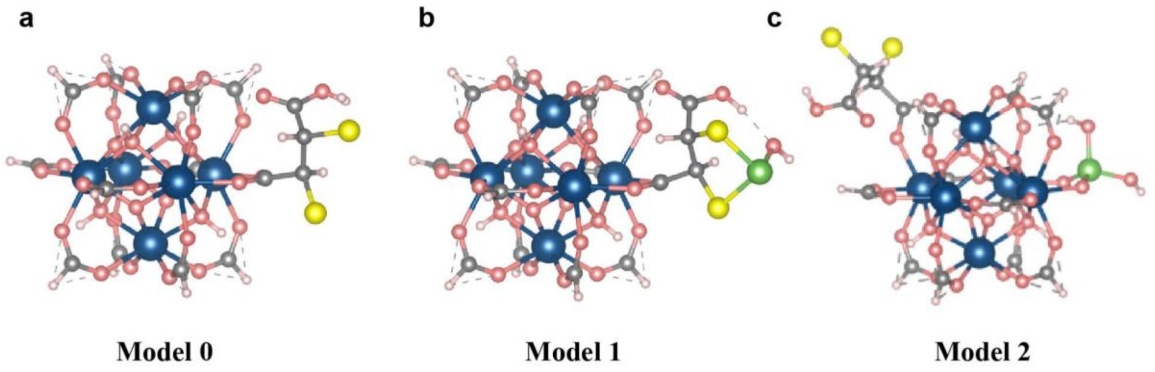

Figure S30 Overview DFT optimized structural models for arsenic adsorption.

Arsenic chelation in rigid structure

Meso-dimercapto succinic acid is a succimer with two stereocenters at carbon position 2(R) and 3(S). If we consider a linear arrangement of the DMSA backbone (with the two carboxylic acid groups extended), the chiral centers enforce a conformation where the two thiol (–SH) groups are oriented on opposite sides of the molecule (**Figure S31 a**). To visualize, that DMSA is not in an optimal configuration for chelating arsenic when incorporated into a rigid structure we performed DFT structure optimization for a structure based on a MIP-203-F (fumaric acid) CIF file in which we replaced - based on our ssNMR results (**Figure S17**) - 50% of the fumaric acid linkers with DMSA (**Figure S31**). We note, that in the resulting optimized structure from DFT all thiol groups face opposite sites which is likely due to the constraint imposed by the coordination of the linker in neighboring metal nodes. Based on the fact, that we did not observe signs of sulfur-arsenic interaction in crystalline Zr-DMSA-bct, unlike in highly amorphous Zr-DMSA-fcu, together with the fact that in this orientation, arsenic chelation within the same molecule is unlikely without rotational freedom of the carboxylic acid groups to bring the thiols onto the same side, we propose an adsorption model in which the DMSA linker is coordinated to the Zr-oxo cluster through only one carboxylic acid group (**Figure S31 c, model 1**) . This binding mode permits rotation of the DMSA molecule, enabling the thiol groups to chelate arsenic.

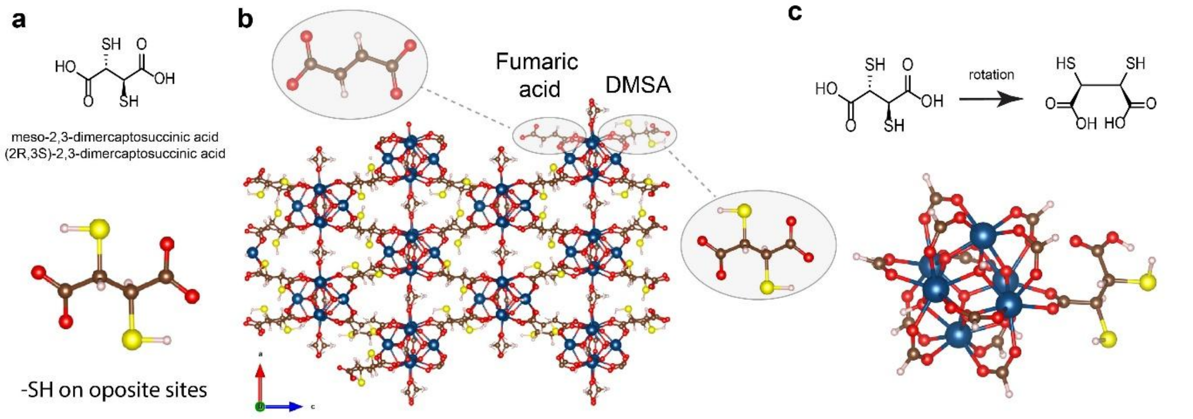

Figure S31 Structural representation of a) DMSA molecule b) mixed linker MOF from DFT optimized CIF file, c) DFT optimized structure where the DMSA linker is attached trough one carboxylate to the cluster allowing it to rotate for chelatation.

Arsenic adsorption - additional considerations

calculated PDF and differential PDF of adsorption model 1

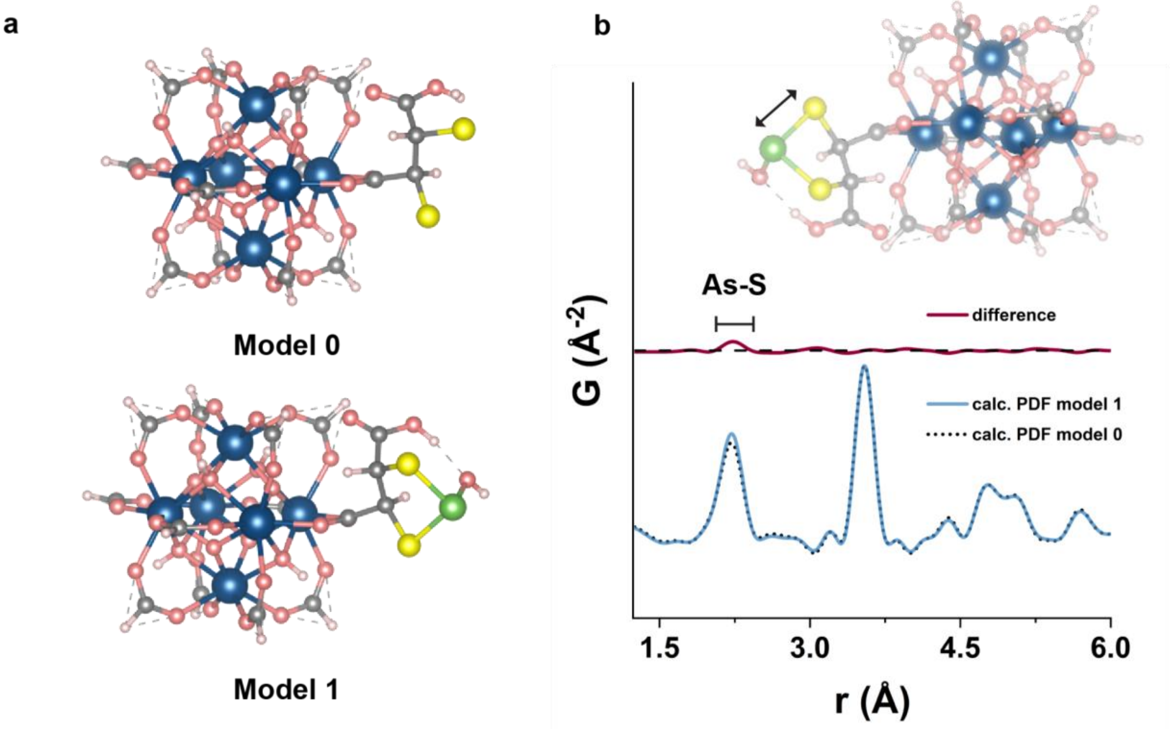

Figure S32 Differential PDF of model 1; a) DFT optimized structural model of Zr_6-cluster including one DMSA ligand with (model 1) and without As(III) (model 0) coordinated via DMSA linker, b) calculated pair distribution functions (PDFs) for both model 0 and model 1, along with the differential PDF highlighting the differences between model 0 and model 1.

calculated PDF and differential PDF of adsorption model 2

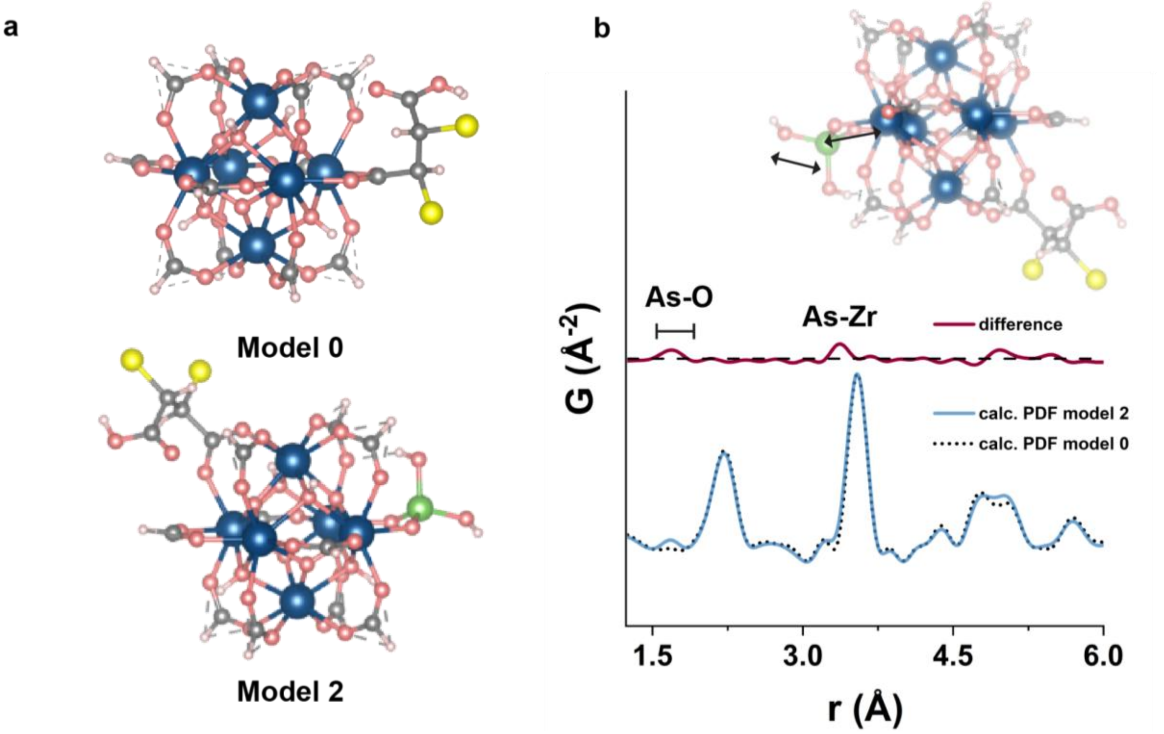

Figure S 33 Differential PDF of model 2; a) DFT optimized structural model of Zr_6-cluster including one DMSA ligand with (model 2) and without As(V) (model 0) coordinated via cluster, b) calculated pair distribution functions (PDFs) for both model 0 and model 2, along with the differential PDF highlighting the differences between model 0 and model 2.

Differential PDF of As(III) Zr-DMSA-fcu and UiO-66

Complementary to the XAS results, we further conducted PDF analysis of As(III) loaded Zr-DMSA-fcu and thiol free Zr-BDC. The differential PDF (dPDF) of As(III) loaded and pristine Zr-DMSA-fcu reveals a new signal around 2.27 Å, which overlaps with Zr-O peaks in the Zr-DMSA-fcu sample (**Figure S34 c**). This bond distance matches very well our bond distance of As-S in adsorption model 1 (2.24 Å, As-S) as well as our experimentally found As-S signal in the EXAFs (2.28 Å). Importantly, we clearly do not observe this signal in the dPDF of thiol free Zr-BDC sample.

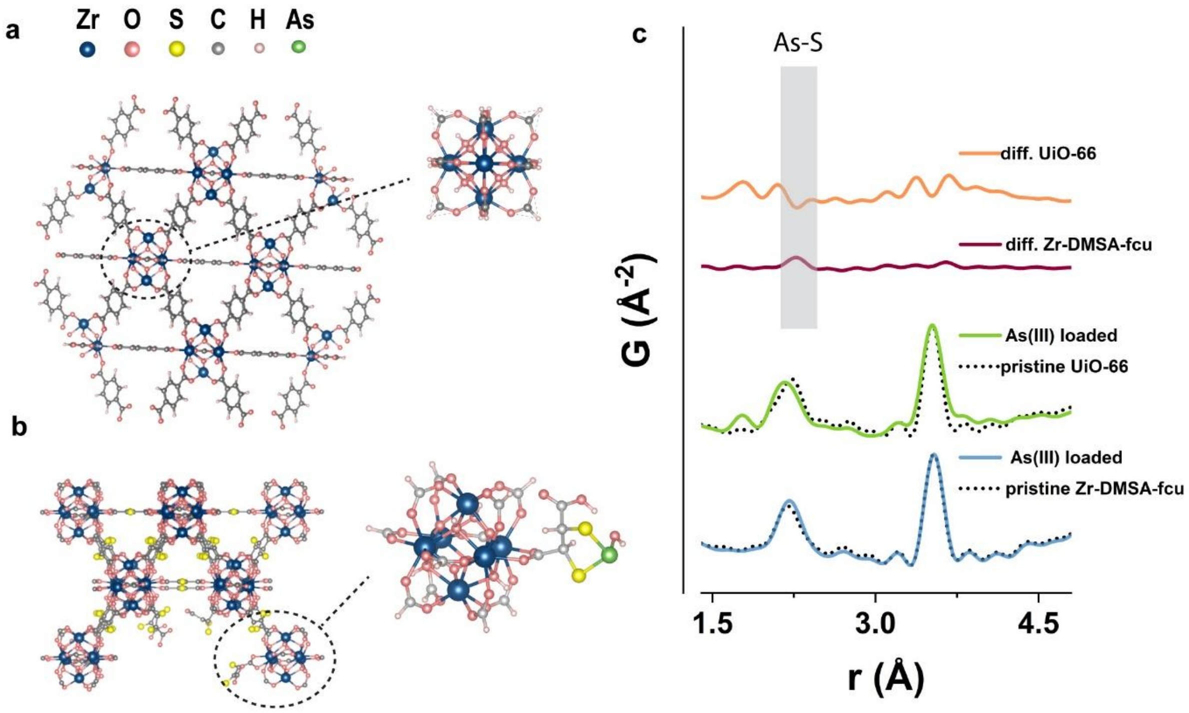

Figure S34 Analysis of differential PDF of As(III) loaded Zr-DMSA-fcu and Zr-BDC; Structural representation of a) Zr-BDC, and b) Zr-DMSA-fcu including adsorption model 1 to visualize As-S interaction, c) experimental PDFs of pristine material and material after As(III) adsorption along with the calculated differential PDF for Zr-DMSA-fcu (blue and red) and Zr-BDC (green and orange).

X-ray absorption spectroscopy (XAS) analysis

XANES fitting for Zr-DMSA-fcu

XANES and EXAFS for both Zr-DMSA-fcu exposed to As(V) as well as As(III) both show clear mixed valence state as well as As-S bond formation (**Figure S35**). In an attempt to quantify the As-S and As-O interaction, XANES data for arsenic exposed Zr-DMSA-fcu was fitted using standards representing As(III)-O ($NaAsO_2$), As(V)-O (As_2O_5) and As(III)-S (As_2S_3). Peak fitting was performed on the three standard compounds within a fitting range of -20 to 25 eV using normalized XAS data. The background was accounted for by a step-function arctangent, where the height, center, and width were fixed, while the main peak was fitted using a Lorentzian function with the area and position as free parameters (**Table S21**). We note that the absorption edge for As(III)-O ($NaAsO_2$) is shifted to higher energy (~1.5 eV) relative to As(III)-S (As_2S_3) in orpiment.[16] Next, for the experimental spectra, deconvolution was again performed using an arctangent function to account for absorption step, with peak positions for arsenic oxidation states were fitted with a Lorentzian curve constrained to the peak position of those determined from the standards. The oxidation state was quantified based on the area ratios of the corresponding Lorentzian peaks, providing a direct measure of the relative proportions of arsenic species.[17,18] **Figure S35 a** shows that the data for As(V) exposed sample is dominated by As(V)-O interaction (approx. 75 %). However, we also find that part of the As(V) is reduced to As(III). For As(III), the As(III)-S standard was included in the fit making up 25%. Overall, this indicates that As(V) is adsorbed through As-O bonds, whereas As(V) which is reduced to As(III) is mainly absorbed through As-S interaction. On the other hand, to fit the As(III) exposed sample only As(III)-S (As_2S_3) and As(III)-O ($NaAsO_2$) standards were fitted revealing a ratio of approx. 2:1 As-S to As-O. This again indicates that As(III) is adsorbed mainly through As-S interaction and matches well for our assumed adsorption model 1.

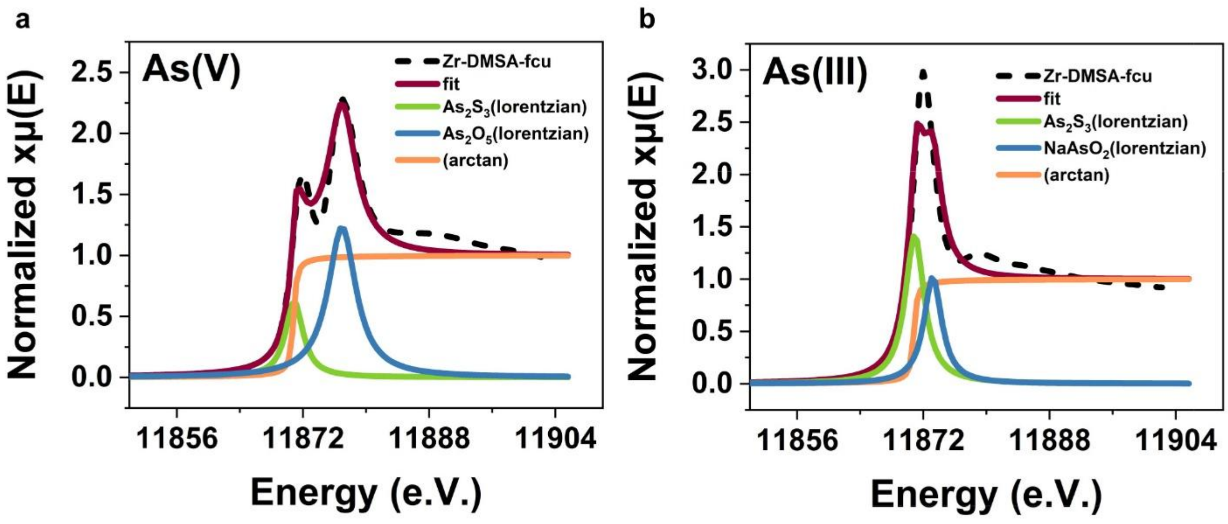

Figure S35 Fitting of XANES for Zr-DMSA-fcu exposed to As(V) and As(III) solution.

Table S20 Overview XANES standard fits

Sample	peak area	peak center	R factor	χ^2	red χ^2
As_2S_3	9.243	11870.9 eV.	0.0035	0.49	0.0040
$NaAsO_2$	11.996	11873.2 eV.	0.0093	1.35	0.0109
As_2O_5	14.425	11876.95 eV.	0.0075	1.23	0.0100

Table S21 Overview XANES oxidation state fits for Zr-DMSA-fcu

Sample	As_2S_3 peak area	As_2O_5 peak area	$NaAsO_2$ peak area	As_2S_3 peak center	As_2O_5 peak center	$NaAsO_2$ peak center	R factor	χ^2	red χ^2
Zr-DMSA-fcu As(III)	6.504	-	4.373	11870.9 eV.	-	11873.2 eV.	0.012	1.53	0.012
Zr-DMSA-fcu As(V)	2.655	8,363	-	11870.9 eV.	11876.9 eV.	-	0.005	0.72	0.005

Extended X-ray absorption fine structure (EXAFS) fitting for Zr-DMSA-fcu

To complement the results from differential PDF and XANES data, EXAFs of the As edge for As(III) exposed samples was also fitted. The interaction was assumed to proceed purely through ligand arsenic interaction, hence the model 1 was employed for the fitting and the coordination number for oxygen was fixed to 1. Next, the k-space was used from 3-12.6 A^{-1} whereas the R-space was fitted from 1 to 2.5 Å with an amplitude reduction factor S_0^2 was fixed to 0.7 (obtained from fitting a standard - As_2O_5- during beam time). The fitting result presented in **Figure 5c** shows a coordination number (C.N) of oxygen of 1, whereas the sulfur was 2.59 ± 0.42. The R-factor of 0.012 indicates a good fit which again supports the hypothesis that the arsenite adsorption mainly proceeds through covalent sulfur-arsenic interactions through the linker. Furthermore, the calculated bond length for As-O was 1.78 Å, and 2.28 Å for As-S which are close to bond length found in the model (As-O, 1.82 Å and As-S 2.24 Å).

Table S22 Overview EXAFS fits

Sample	As-O C.N.	As-S C.N.	As-S bond length (Å)	As-O bond length (Å)	As-O σ^2 / $Å^2$	As-S σ^2 / $Å^2$	ΔE_0/eV	R-factor
Zr-DMSA-fcu As(III)	1	2.59 ± 0.42	2.28	1.78	0.00436	0.00295	8.26 ± 1.5	0.012
model 1	1	2	2.24 A	1.82	-	-	-	-

River water experiment

Figure S36 and **Figure S37** show batch adsorption experiment where river water was spiked with 1 ppm As(III) and As(V), respectively. Note, that the concentration of arsenic was measured using IC-MS, while the concentration of metals was measured using ICP-OES. Anions (SO_4^{2-}, NO_3^- and NO_2^-, Cl^-, and F^-) were measured using Ion chromatography. To measure the carbonate concentration, we measured the total inorganic carbon (IOC). Before the adsorption experiment, the carbonate concentration for both As(III) and As(V) solution was 9 [mgC/L], which was reduced to below 3 [mgC/L] (which was the detectable limit of quantification for this measurement) after the experiment.

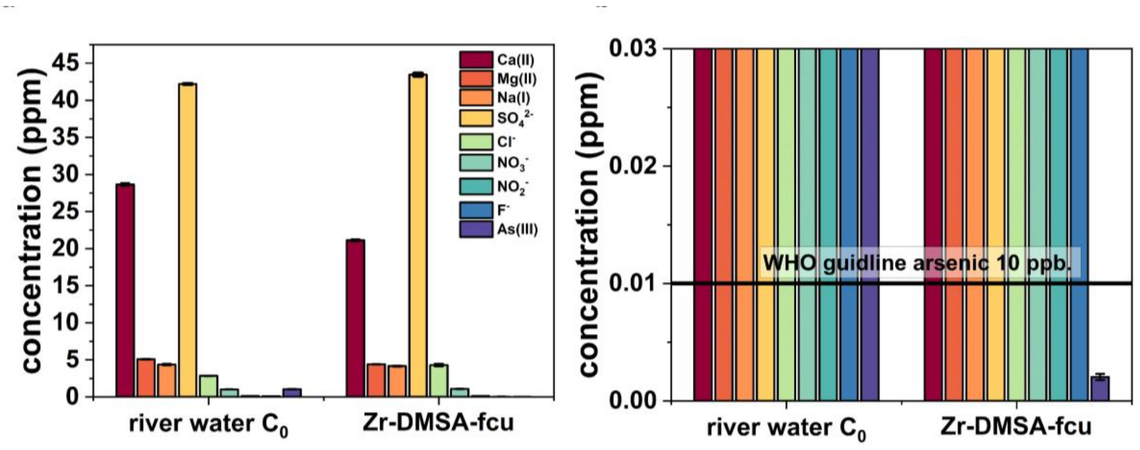

Figure S36 Batch adsorption experiments of As(III) spiked river water. a) shows the concentration of As(III) and various other ions before (C_0) and after exposure to Zr-DMSA-fcu, and b) shows zoom in to low concentration of complex water mixture before (C_0) and after exposure to Zr-DMSA. Conditions: Initial concentration arsenic (1 ppm (concentration of other ions was measured), volume (10 mL), adsorbent dosage (0.5 g/L), adsorption time (24 h).

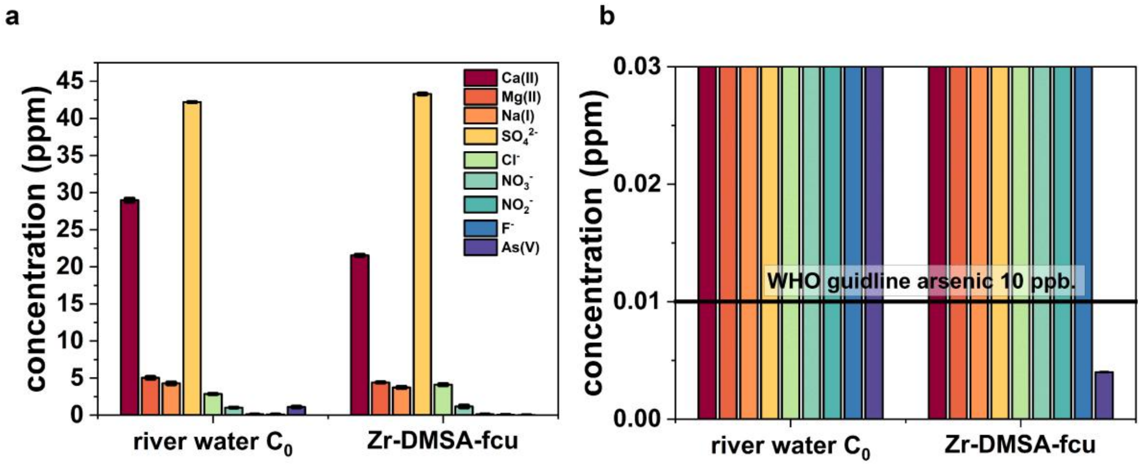

Figure S37 Batch adsorption experiments of As(V) spiked river water. a) shows the concentration of As(V) and various other ions before (C_0) and after exposure to Zr-DMSA-fcu, and b) shows zoom in to low concentration of complex water mixture before (C_0) and after exposure to Zr-DMSA. Conditions: Initial concentration arsenic (1 ppm (concentration of other ions was measured), volume (10 mL), adsorbent dosage (0.5 g/L), adsorption time (24 h).

Reproducibility of Zr-DMSA

Figure S38 shows the characterization of three independently synthesized Zr-DMSA-fcu batches all showing similar PXRD pattern, N_2 adsorption isotherm, TGA and a consistent arsenic adsorption performance. Additionally, combustion elemental analysis shows a similar sulfur content for all batches (**Table S23**).

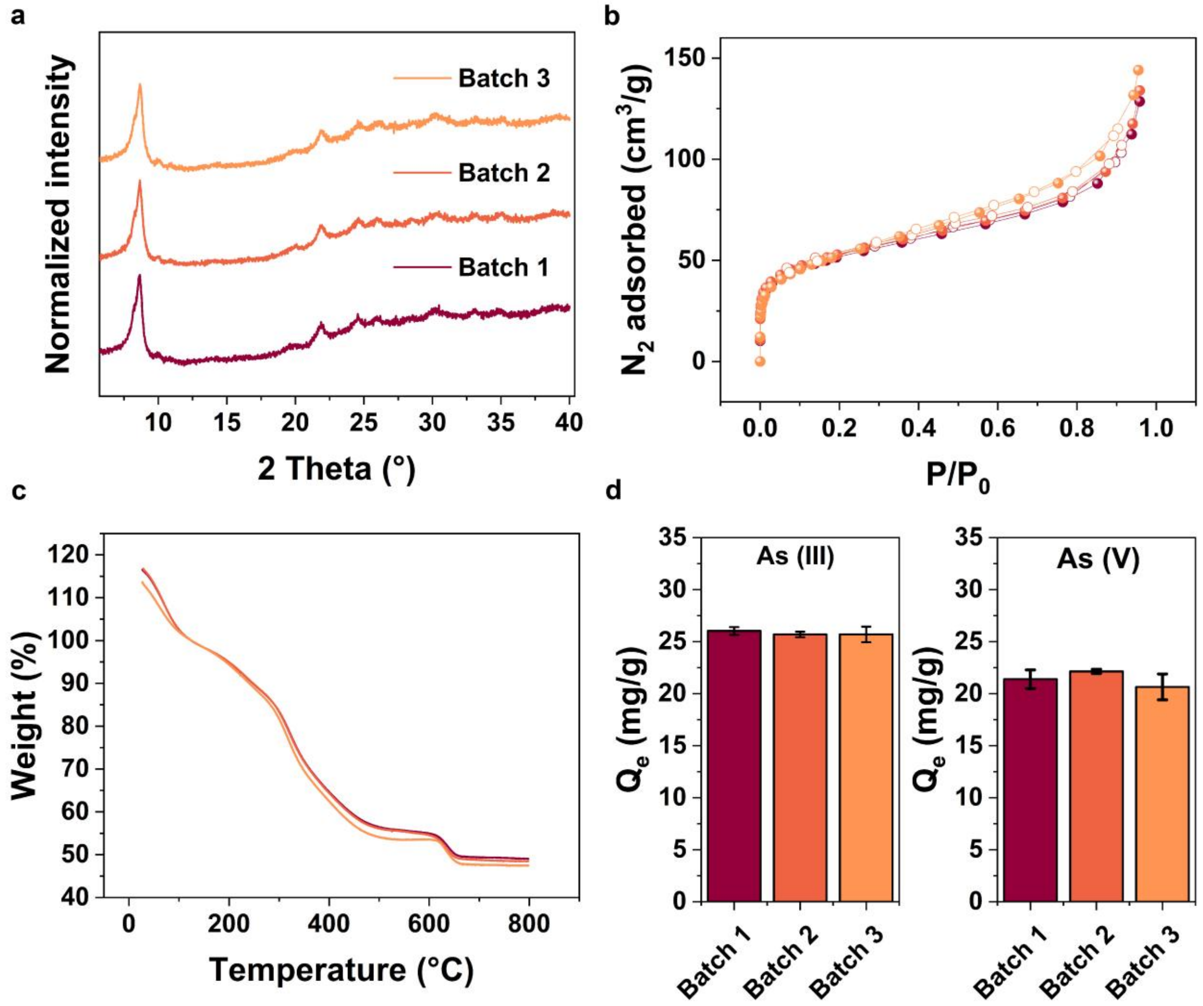

Figure S38 Characterization of three independently synthesized batch of Zr-DMSA-fcu: a) PXRD pattern, b) nitrogen adsorption isotherm at 77K, c) TGA normalized at 100 °C and d) As(III) and As(V) adsorption experiments; Conditions: Initial concentration arsenic (15 ppm), volume (20 mL), adsorbent dosage (0.5 g/L), adsorption time (24 h) and pH (7).

Table S23 Combustion elemental analysis of three independently synthesized Zr-DMSA-fcu batches

Sample	Sulfur (%)	Carbon (%)	Hydrogen (%)
Batch 1	14.03 ± 0.01	14.53 ± 0.04	2.15 ± 0.01
Batch 2	14.56 ± 0.10	15.26 ± 0.10	2.01 ± 0.02
Batch 3	14.81 ± 0.10	15.12 ± 0.05	2.31 ± 0.02
Average	14.46 ± 0.31	14.97 ± 0.31	2.15 ± 0.12

References

[1] M. Wahiduzzaman, S. Wang, B. J. Sikora, C. Serre, G. Maurin, *Chem. Commun.* **2018**, *54*, 10812–10815.

[2] P. Yang, Y. Shu, Q. Zhuang, Y. Li, J. Gu, *Langmuir* **2019**, *35*, 16226–16233.

[3] K. Momma, F. Izumi, *J. Appl. Crystallogr.* **2011**, *44*, 1272–1276.

[4] S. Wang, N. Xhaferaj, M. Wahiduzzaman, K. Oyekan, X. Li, K. Wei, B. Zheng, A. Tissot, J. Marrot, W. Shepard, C. Martineau-Corcos, Y. Filinchuk, K. Tan, G. Maurin, C. Serre, *J. Am. Chem. Soc.* **2019**, *141*, 17207–17216.

[5] H. Furukawa, F. Gándara, Y.-B. Zhang, J. Jiang, W. L. Queen, M. R. Hudson, O. M. Yaghi, *J. Am. Chem. Soc.* **2014**, *136*, 4369–4381.

[6] J. W. M. Osterrieth, J. Rampersad, D. Madden, N. Rampal, L. Skoric, B. Connolly, M. D. Allendorf, V. Stavila, J. L. Snider, R. Ameloot, J. Marreiros, C. Ania, D. Azevedo, E. Vilarrasa-Garcia, B. F. Santos, X. Bu, Z. Chang, H. Bunzen, N. R. Champness, S. L. Griffin, B. Chen, R. Lin, B. Coasne, S. Cohen, J. C. Moreton, Y. J. Colón, L. Chen, R. Clowes, F. Coudert, Y. Cui, B. Hou, D. M. D'Alessandro, P. W. Doheny, M. Dincă, C. Sun, C. Doonan, M. T. Huxley, J. D. Evans, P. Falcaro, R. Ricco, O. Farha, K. B. Idrees, T. Islamoglu, P. Feng, H. Yang, R. S. Forgan, D. Bara, S. Furukawa, E. Sanchez, J. Gascon, S. Telalović, S. K. Ghosh, S. Mukherjee, M. R. Hill, M. M. Sadiq, P. Horcajada, P. Salcedo-Abraira, K. Kaneko, R. Kukobat, J. Kenvin, S. Keskin, S. Kitagawa, K. Otake, R. P. Lively, S. J. A. DeWitt, P. Llewellyn, B. V. Lotsch, S. T. Emmerling, A. M. Pütz, C. Martí-Gastaldo, N. M. Padial, J. García-Martínez, N. Linares, D. Maspoch, J. A. Suárez Del Pino, P. Moghadam, R. Oktavian, R. E. Morris, P. S. Wheatley, J. Navarro, C. Petit, D. Danaci, M. J. Rosseinsky, A. P. Katsoulidis, M. Schröder, X. Han, S. Yang, C. Serre, G. Mouchaham, D. S. Sholl, R. Thyagarajan, D. Siderius, R. Q. Snurr, R. B. Goncalves, S. Telfer, S. J. Lee, V. P. Ting, J. L. Rowlandson, T. Uemura, T. Iiyuka, M. A. Van Der Veen, D. Rega, V. Van Speybroeck, S. M. J. Rogge, A. Lamaire, K. S. Walton, L. W. Bingel, S. Wuttke, J. Andreo, O. Yaghi, B. Zhang, C. T. Yavuz, T. S. Nguyen, F. Zamora, C. Montoro, H. Zhou, A. Kirchon, D. Fairen-Jimenez, *Adv. Mater.* **2022**, *34*, 2201502.

[7] R. L. Johnson, K. Schmidt-Rohr, *J. Magn. Reson.* **2014**, *239*, 44–49.

[8] D. Massiot, F. Fayon, M. Capron, I. King, S. Le Calvé, B. Alonso, J. Durand, B. Bujoli, Z. Gan, G. Hoatson, *Magn. Reson. Chem.* **2002**, *40*, 70–76.

[9] G. Ashiotis, A. Deschildre, Z. Nawaz, J. P. Wright, D. Karkoulis, F. E. Picca, J. Kieffer, *J. Appl. Crystallogr.* **2015**, *48*, 510–519.

[10] P. Juhás, T. Davis, C. L. Farrow, S. J. L. Billinge, *J. Appl. Crystallogr.* **2013**, *46*, 560–566.
[11] G. Igel-Mann, H. Stoll, H. Preuss, *Mol. Phys.* **1988**, *65*, 1321–1328.
[12] D. Andrae, U. Häußermann, M. Dolg, H. Stoll, H. Preuß, *Theor. Chim. Acta* **1990**, *77*, 123–141.
[13] J. Pulparayil Mathew, C. Simms, D. E. Salazar Marcano, E. Dhaene, T. N. Parac-Vogt, J. De Roo, *Adv. Sci.* **2025**, DOI 10.1002/advs.202504713.
[14] M. C. Simoes, K. J. Hughes, D. B. Ingham, L. Ma, M. Pourkashanian, *Inorg. Chem.* **2017**, *56*, 7566–7573.
[15] A. F. Holleman, E. Wiberg, N. Wiberg, *Lehrbuch Der Anorganischen Chemie*, De Gruyter, Berlin, **2007**.
[16] M. J. Katz, Z. J. Brown, Y. J. Colón, P. W. Siu, K. A. Scheidt, R. Q. Snurr, J. T. Hupp, O. K. Farha, *Chem. Commun.* **2013**, *49*, 9449.
[17] Y. Takahashi, N. Ohtaku, S. Mitsunobu, K. Yuita, M. Nomura, *Anal. Sci.* **2003**, *19*, 891–896.
[18] M. Chen, Y. Yang, W. Liu, C. Wang, B. Johannessen, *Hydrometallurgy* **2018**, *175*, 11–19.
